# Mapping dysfunctional circuits in the frontal cortex using deep brain stimulation

**DOI:** 10.1038/s41593-024-01570-1

**Published:** 2024-02-22

**Authors:** Barbara Hollunder, Jill L. Ostrem, Ilkem Aysu Sahin, Nanditha Rajamani, Simón Oxenford, Konstantin Butenko, Clemens Neudorfer, Pablo Reinhardt, Patricia Zvarova, Mircea Polosan, Harith Akram, Matteo Vissani, Chencheng Zhang, Bomin Sun, Pavel Navratil, Martin M. Reich, Jens Volkmann, Fang-Cheng Yeh, Juan Carlos Baldermann, Till A. Dembek, Veerle Visser-Vandewalle, Eduardo Joaquim Lopes Alho, Paulo Roberto Franceschini, Pranav Nanda, Carsten Finke, Andrea A. Kühn, Darin D. Dougherty, R. Mark Richardson, Hagai Bergman, Mahlon R. DeLong, Alberto Mazzoni, Luigi M. Romito, Himanshu Tyagi, Ludvic Zrinzo, Eileen M. Joyce, Stephan Chabardes, Philip A. Starr, Ningfei Li, Andreas Horn

**Affiliations:** 1 Movement Disorders and Neuromodulation Unit, Department of Neurology, Charité – Universitätsmedizin Berlin, Berlin, Germany; 2 Einstein Center for Neurosciences Berlin, Charité – Universitätsmedizin Berlin, Berlin, Germany; 3 Berlin School of Mind and Brain, Humboldt-Universität zu Berlin, Berlin, Germany; 4 Movement Disorders and Neuromodulation Centre, Department of Neurology, University of California, San Francisco, San Francisco, CA USA; 5 Center for Brain Circuit Therapeutics, Department of Neurology, Brigham and Women’s Hospital, Harvard Medical School, Boston, MA USA; 6 Department of Neurosurgery, Massachusetts General Hospital, Harvard Medical School, Boston, MA USA; 7 Department of Psychiatry and Psychotherapy, Charité – Universitätsmedizin Berlin, Berlin, Germany; 8 Université Grenoble Alpes, Grenoble, France; 9 Inserm, U1216, Grenoble Institut des Neurosciences, Grenoble, France; 10 Department of Psychiatry, Centre Hospitalier Universitaire Grenoble Alpes, Grenoble, France; 11 Unit of Functional Neurosurgery, UCL Queen Square Institute of Neurology, London, UK; 12 Victor Horsley Department of Neurosurgery, The National Hospital for Neurology and Neurosurgery, London, UK; 13 The BioRobotics Institute, Scuola Superiore Sant’Anna, Pisa, Italy; 14 Department of Excellence in Robotics and AI, Scuola Superiore Sant’Anna, Pisa, Italy; 15 Department of Neurosurgery, Rujin Hospital, Shanghai Jiao Tong University School of Medicine, Shanghai, China; 16 Department of Neurology, University Hospital Würzburg, Würzburg, Germany; 17 Department of Neurological Surgery, University of Pittsburgh, Pittsburgh, PA USA; 18 Department of Psychiatry and Psychotherapy, Faculty of Medicine and University Hospital Cologne, University of Cologne, Cologne, Germany; 19 Department of Neurology, Faculty of Medicine and University Hospital Cologne, University of Cologne, Cologne, Germany; 20 Department of Stereotactic and Functional Neurosurgery, Faculty of Medicine and University Hospital Cologne, University of Cologne, Cologne, Germany; 21 Clinic of Pain and Functional Neurosurgery, São Paulo, Brazil; 22 Department of Neurology and Neurosurgery, University of Caxias do Sul, Rio Grande do Sul, Brazil; 23 Department of Neurology, Charité – Universitätsmedizin Berlin, Berlin, Germany; 24 NeuroCure Cluster of Excellence, Charité – Universitätsmedizin Berlin, Berlin, Germany; 25 Department of Psychiatry, Massachusetts General Hospital, Harvard Medical School, Boston, MA USA; 26 Edmond and Lily Safra Center for Brain Sciences, The Hebrew University, Jerusalem, Israel; 27 Department of Medical Neurobiology, Institute of Medical Research Israel-Canada, The Hebrew University, Hadassah Medical School, Jerusalem, Israel; 28 Department of Neurosurgery, Hadassah Medical Center, Jerusalem, Israel; 29 Department of Neurology, Emory University School of Medicine, Atlanta, GA USA; 30 Parkinson and Movement Disorders Unit, Fondazione IRCCS Istituto Neurologico Carlo Besta, Milan, Italy; 31 Department of Neuropsychiatry, The National Hospital for Neurology and Neurosurgery, London, UK; 32 Department of Neurosurgery, Centre Hospitalier Universitaire Grenoble Alpes, Grenoble, France; 33 Department of Neurological Surgery, University of California, San Francisco, San Francisco, CA USA

**Keywords:** Cognitive neuroscience, Brain, Movement disorders, Psychiatric disorders, Neural circuits

## Abstract

Frontal circuits play a critical role in motor, cognitive and affective processing, and their dysfunction may result in a variety of brain disorders. However, exactly which frontal domains mediate which (dys)functions remains largely elusive. We studied 534 deep brain stimulation electrodes implanted to treat four different brain disorders. By analyzing which connections were modulated for optimal therapeutic response across these disorders, we segregated the frontal cortex into circuits that had become dysfunctional in each of them. Dysfunctional circuits were topographically arranged from occipital to frontal, ranging from interconnections with sensorimotor cortices in dystonia, the primary motor cortex in Tourette’s syndrome, the supplementary motor area in Parkinson’s disease, to ventromedial prefrontal and anterior cingulate cortices in obsessive-compulsive disorder. Our findings highlight the integration of deep brain stimulation with brain connectomics as a powerful tool to explore couplings between brain structure and functional impairments in the human brain.

## Abstract (web summary)

Hollunder et al. identify networks where deep brain stimulation reduces symptoms for Parkinson’s disease, Tourette’s syndrome, dystonia and obsessive-compulsive disorder. This revealed a fronto-rostral topography that segregates the frontal cortex.

## Main

Studying brain connectomics with deep brain stimulation (DBS) represents a compelling framework for identifying circuits that associate with successful neuromodulation therapy^1,2^. This potential arises from modeling structural connections activated by variably placed electrodes across patients and relating modulated connections to symptom improvements^1,3^. As a first-order approximation, effective DBS is seen to act akin to a functional lesion^4^, achieving downregulation of dysfunctional networks that were involved in neurological or neuropsychiatric symptoms in the first place. By isolating circuits that exhibit the most favorable response to DBS interventions, we, hence, advance understanding of the precise brain circuits associated with dysfunctions in particular disorders^1,3^. As such, this methodology can be used to outline the human ‘dysfunctome’—that is, the set of connections that are disrupted in given brain disorders—and may be tuned down by successful neuromodulation.

Disrupted interactions between the frontal cortex and basal ganglia lie at the root of numerous brain disorders. These interconnections govern motor, cognitive and affective functions^5^ and are implemented as fronto-subcortical circuits that cross-communicate^6,7^ but retain a certain degree of segregation at cortical, striatal, pallidal/nigral and thalamic levels^6,8^. Although the striatum has often been described as the primary input structure within the basal ganglia, the subthalamic nucleus (STN) has recently been recognized as a second direct input nucleus^9^. The STN is much smaller than the striatum (~240 mm^3^)^10^ but similarly receives efference copies of projections from the entire frontal cortex^6^. This property renders the STN an ideal gateway for modulating large-scale brain networks through direct electrical stimulation delivered by invasive electrodes.

Indeed, targeting the same nucleus has proven an effective therapy for a heterogeneous spectrum of disorders that includes Parkinson’s disease (PD)^11^, dystonia (DYT)^12,13^, obsessive-compulsive disorder (OCD)^14,15^ and Tourette’s syndrome (TS)^16,17^. At first glance, it may appear paradoxical that applying electrical stimulation to a subcortical structure of such constrained extent could alleviate symptoms in four disorders that manifest as differently from one another at a phenotypical level. However, this seeming paradox may open a unique opportunity. Because the same compact nucleus is used as a DBS target for different disorders, it acts as a network node that provides therapeutic access to malfunctioning circuits in each of these conditions. By isolating circuits whose modulation entails the most substantial treatment benefit, we may be able to disentangle whether one and the same—or, rather, multiple different—dysfunctional networks are implicated in these multiform phenotypic presentations.

Here we apply this concept by integrating 534 DBS electrodes—each implanted for treatment of DYT, PD, TS or OCD symptoms—and their corresponding clinical outcomes with detailed structural connectomes of the human brain. We analyze the dataset on both local and global network levels by applying DBS Sweet Spot Mapping^18^ and DBS Fiber Filtering^19^. The resulting circuits segregate the frontal cortex and its hyperdirect and indirect pathway connections with the STN into distinct dysfunctional territories. We base this work on broad definitions of cardinal symptoms present in each of these disorders (as measured by established rating scales used in clinical practice).

## Results

### Patient demographics and clinical results

#### Discovery cohorts

**Fig. 1:**
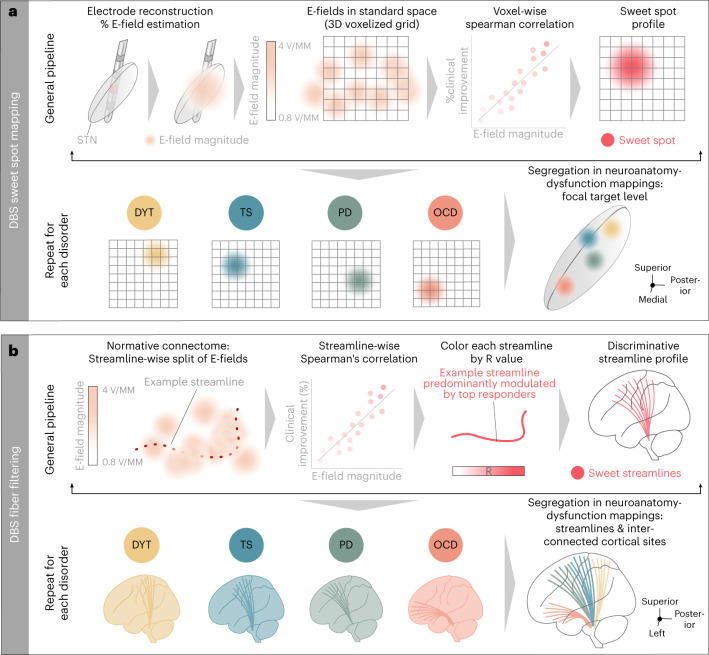
Overview of the twofold group-level approach of (sub)cortical dysfunction mapping. **a**, DBS Sweet Spot Mapping^18^. Patient-specific electrode reconstructions were first derived relative to their precise position within the STN region and integrated with individual stimulation parameters to estimate E-field magnitudes. Subsequently, Spearman’s rank correlations between E-field magnitudes and clinical improvements were performed (separately for each disease). Applying this procedure across voxels resulted in a detailed grid of positively (sweet spot) and negatively (sour spot, not shown here) associated stimulation sites. **b**, DBS Fiber Filtering^19^. Each streamline within a predefined normative connectome was weighted by its ability to discern good from poor responders in each respective cohort. To do so, the peak E-field magnitudes among samples drawn along the course of each streamline were Spearman’s rank correlated with clinical outcomes. Streamlines predominantly modulated by high E-field magnitudes of good responders received high positive weights (sweet streamlines), whereas those associated with high E-field magnitudes of poor responders were attributed high negative weights (sour streamlines, not represented here).

**Fig. 2:**
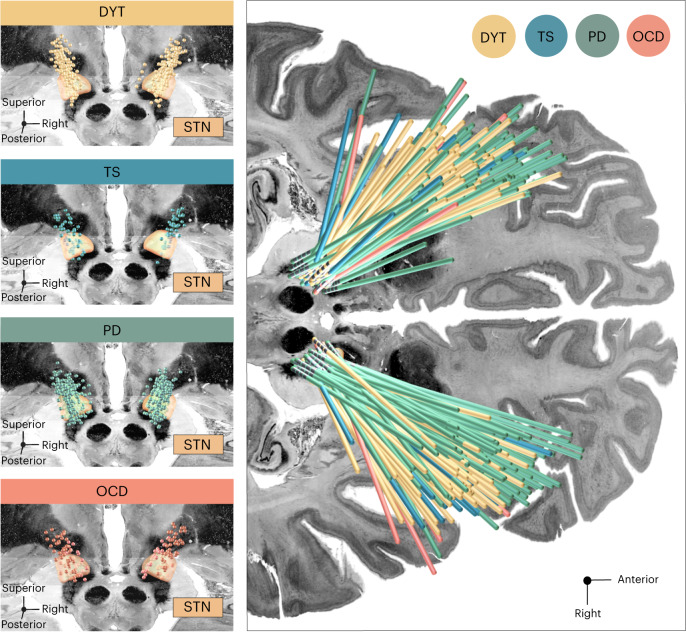
Overview of electrode placements relative to the STN across discovery cohorts. Left panels, DBS electrode placement is shown in relation to a posterior view of the STN in DYT (*n* = 56), PD (*n* = 94), TS (*n* = 14) and OCD (*n* = 19) cohorts, respectively. Electrode contacts are visualized as point clouds. Right panel, visualization of DBS leads of the four discovery cohorts investigated in the present study are featured in the axial plane and colored according to indication. The STN is defined by the DISTAL atlas, version 1.1 (ref. ^28^), with an axial plane of the BigBrain template in 100-µm resolution^66^ displayed as a backdrop (*y* = −5 mm, *z* = −10 mm).

Each of the four disorders was represented by two cohorts of bilaterally implanted STN-DBS patients (*n* = 197, 80 females), amounting to a total of *n* = 394 analyzed DBS electrodes: DYT (*n* = 70, 38 females), PD (*n* = 94, 29 females), OCD (*n* = 19, 10 females) and TS (*n* = 14, three females). Average improvements from DBS ON to baseline were similar between cohorts and centers. In DYT, the San Francisco cohort presented with an average improvement of 52 ± 42% and the Shanghai cohort of 65 ± 29% on the motor subscale of the Burke–Fahn–Marsden Dystonia Rating Scale (BFMDRS). Patients in the TS cohort from Pisa/Milan benefitted by 62 ± 18% and those from Shanghai by 59 ± 24% on the Yale Global Tic Severity Scale (YGTSS). In PD, Berlin patients improved by 45 ± 23% and the Würzburg cohort by 49 ± 24% on the Unified Parkinson’s Disease Rating Scale–Part III (UPDRS-III). DBS entailed a 45 ± 29% reduction within the Yale-Brown Obsessive-Compulsive Scale (Y-BOCS) for the OCD cohort from London after ‘STN-DBS-only’ stimulation, and Grenoble patients improved by 44 ± 32%. A comprehensive summary of demographic and clinical patient characteristics along with DBS and imaging specifications is provided in Supplementary Table 1. Supplementary Tables 2–5 provide detailed patient-specific information. Figure 1 recapitulates applied methodological concepts in graphical form. Electrode localization confirmed electrode placement within the subthalamic region in all patients (Fig. 2).

#### Validation cohorts

Validation of the PD streamline model was based on an additional STN-DBS cohort (*n* = 32, 10 females) from Würzburg, characterized by a mean reduction of 47 ± 21% on the UPDRS-III. In OCD, an additional patient cohort (*n* = 35, 18 females) receiving DBS to the ventral capsule/ventral striatum (VC/VS) region was pooled across Cologne, Boston and London centers. Critically, electrodes of these novel cohorts were entirely independent from those used to create the streamline models (see Methods for the special case of London patients). On average, Cologne patients benefitted from DBS by 31 ± 21%, those from London after ‘VC/VS-DBS only’ by 53 ± 26% and those from Boston by 40 ± 30% on the Y-BOCS. Additional cohort-averaged specifics are featured in Supplementary Table 6, with patient-wise information in Supplementary Table 7 (PD validation cohort) and Supplementary Table 8 (OCD validation cohorts).

Finally, two DBS patients (one with PD and OCD each) were prospectively reprogrammed, and one OCD patient was prospectively implanted and programmed as informed on the streamline models established here (see below for more detailed case vignettes).

### Dysfunction mappings at the subthalamic level

#### Model definition

**Fig. 3:**
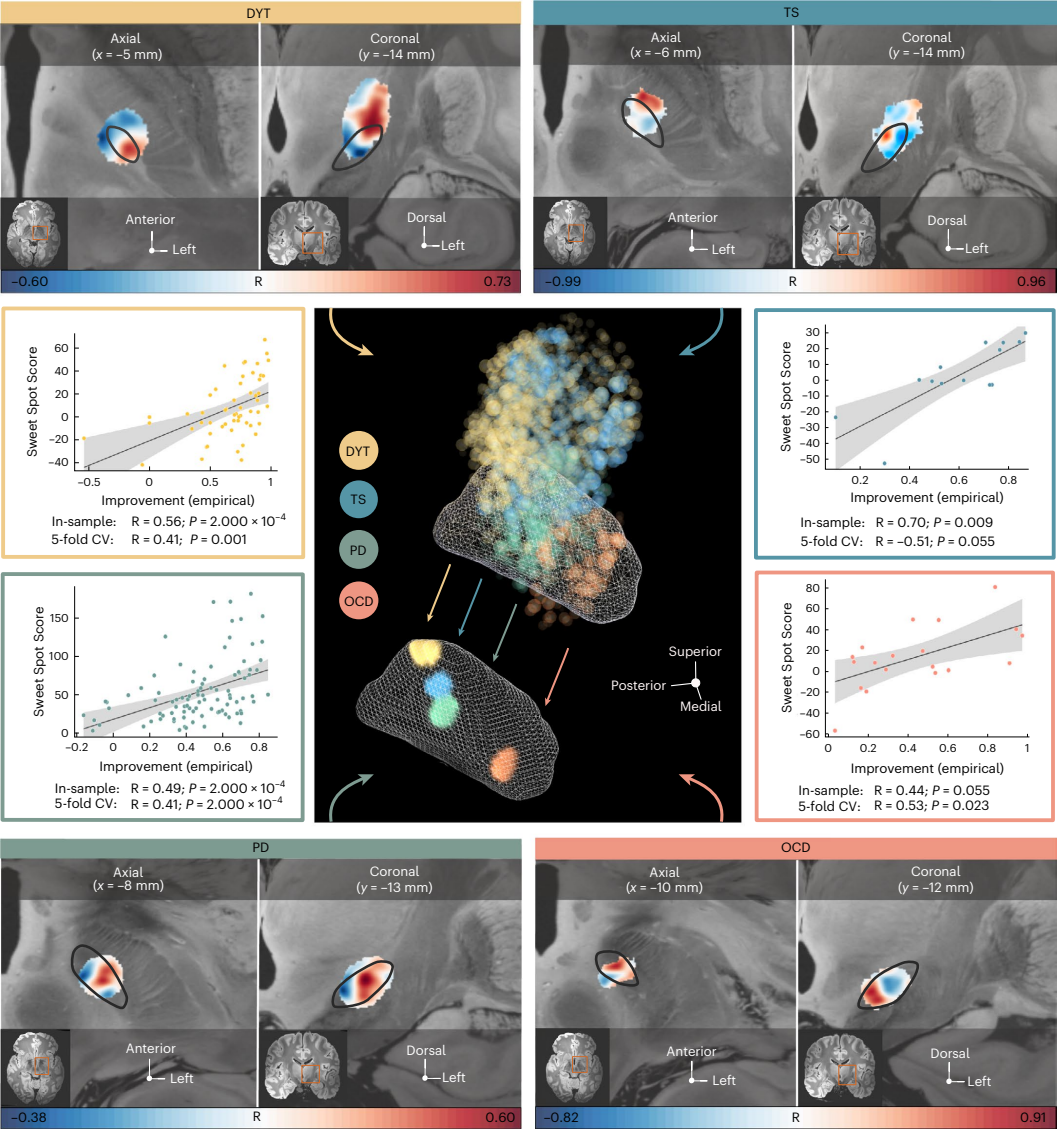
Segregation of dysfunction mappings at the subthalamic level by disease-specific stimulation effects. Middle panel (center), the topographical organization of disorder-specific DBS sweet spots in DYT (*n* = 56), TS (*n* = 14), PD (*n* = 94) and OCD (*n* = 19) is shown as a density cloud plot relative to a three-dimensional model of the left STN in template space derived from the DISTAL atlas, version 1.1 (ref. ^28^). Sphere size and transparency indicate Spearman’s rank correlation strength between stimulation impact and clinical improvements at a given coordinate, with bigger and less transparent spheres coding for higher correlations. Below, binarized and thresholded sweet spot peaks are projected onto the STN surface. Top and bottom panels, axial and coronal views of sweet and sour spots are displayed relative to the left STN (black outlines), independently for each disorder, superimposed onto a 100-µm ex vivo brain template^67^. Voxels are color-coded by degree of Spearman’s rank correlation (warm colors for positive associations and cool colors for negative associations) between E-field magnitudes and clinical improvements. Middle panel (left and right), Spearman’s correlation plots show amounts of clinical outcome variance explained by spatial similarity of E-field peaks with disease-wise sweet spot models (expressed as Sweet Spot Score under each E-field, averaged for bilateral scores per patient) across the cohort (two-sided tests). Gray shaded areas are representative of 95% confidence intervals.

Disease-wise stimulation effects were mapped into anatomical space at the subthalamic level. A caudo-rostral latero-medial organization emerged for peak voxels associated with beneficial stimulation ranging from DYT to TS, PD and OCD (Fig. 3, center). This result was consistent with functional zones commonly associated with anatomical portions of the nucleus (DYT in the sensorimotor, TS in the motor, PD in the motor-premotor and OCD in the associative-limbic domain). A detailed overview of the anatomical localization of sweet and sour spots for each disease is provided in Fig. 3 (top and bottom panels). Peak voxel coordinates are reported in Supplementary Table 9.

#### Estimation of outcomes based on the model

Spatial correlations between electric fields (E-fields) and the optimal pattern were performed to confirm that the sweet spot model could explain variance in clinical outcomes (Fig. 3, middle panel, left and right). This analysis was carried out (1) to compare results between disorders and (2) to compare amounts of variance accounted for by sweet spots versus sweet streamlines. Critically, these in-sample analyses were circular in nature and should, thus, not be overinterpreted. To account for this limitation, analyses were subjected to fivefold cross-validation (CV) (Fig. 3, middle panel, left and right) to investigate generalizability of findings, which yielded significant results in all disorders but TS (with the lowest *n*). To demonstrate stability of sweet spot configurations and fivefold CVs across different E-field thresholds, we repeated the procedure with varying thresholds, which led to consistent results (Supplementary Fig. 1).

### Dysfunction mappings at streamline and cortical levels

#### Model definition

**Fig. 4:**
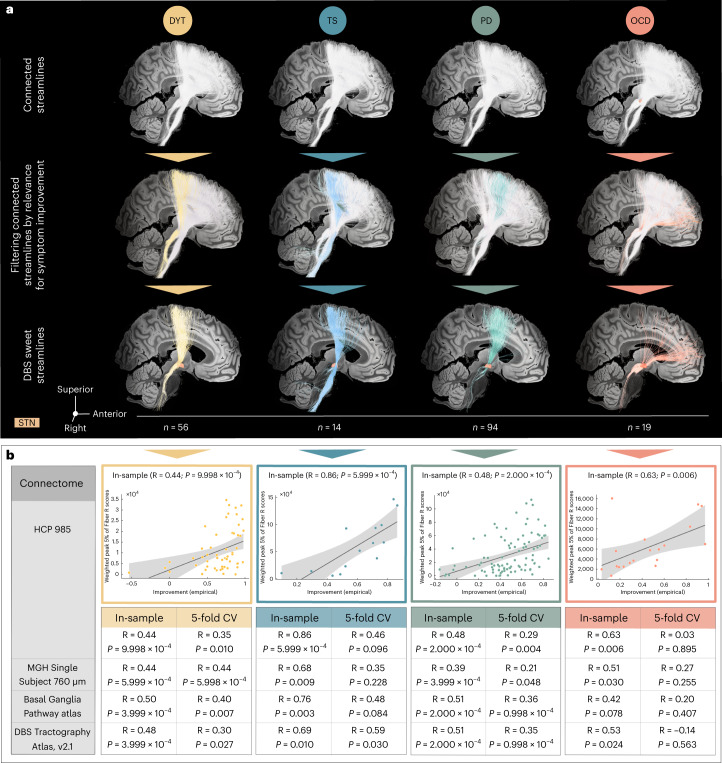
Disease-specific sweet streamline models in each discovery cohort. **a**, Sweet streamlines in DYT (*n* = 56; peak R = 0.36), PD (*n* = 94; peak R = 0.37), TS (*n* = 14; peak R = 0.73) and OCD (*n* = 19; peak R = 0.49) associated with beneficial stimulation outcomes were filtered from a population-based group connectome^21^. The top row demonstrates the set of connections (in white) seeding from stimulation volumes across patients in each of the four disorders. Among these plain connections, only those were isolated via DBS Fiber Filtering (middle row) whose modulation was Spearman’s rank correlated with clinical outcomes (bottom row). Sweet streamlines are highlighted in disease-specific color and displayed in thresholded and binarized fashion. Results are shown against a sagittal slice (*x* = −5 mm) of the 7T MRI ex vivo 100-µm human brain template^67^, in conjunction with a three-dimensional model of the right STN in template space from the DISTAL atlas, version 1.1 (ref. ^28^). **b**, In-sample correlations and fivefold CVs are reported for models informed on four different normative connectomes. Plots in the top row represent the fitting of a linear model to determine the degree to which the overlap of E-field magnitudes with selected HCP 985 Connectome^21^ sweet streamlines explains variance in empirical clinical outcome across the cohort, as calculated using Spearman’s correlation (two-sided tests). The magnitude of E-field overlap with sweet streamline models in this analysis is expressed as weighted peak 5% of Fiber R scores under each E-field, averaged across bilateral scores per patient. Gray shaded areas indicate 95% confidence intervals.

**Fig. 5:**
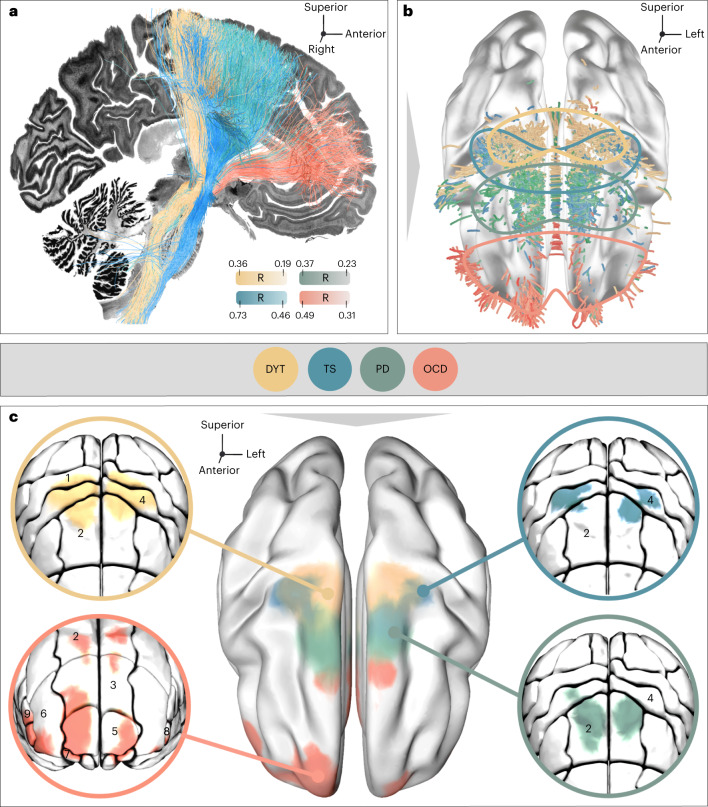
Topography of streamlines and interconnected cortical sites associated with therapeutic stimulation effects. **a**, Segregation into therapeutic networks is achieved by means of DBS Fiber Filtering in DYT (*n* = 56), PD (*n* = 94), TS (*n* = 14) and OCD (*n* = 19). Disease-specific optimal streamlines were isolated from a high-resolution normative group connectome^21^ through association with clinical effects in each disorder. This was achieved using Spearman’s rank correlation between peak E-field magnitudes by which each streamline was modulated and clinical improvements across the cohort. Mappings are displayed in thresholded form, against a sagittal slice (*x* = −5 mm) of a brain cytoarchitecture atlas in ICBM 2009b Nonlinear Asymmetric (‘MNI’) space^66^. Streamline color intensity is representative of R value magnitude, with darker colors corresponding to higher correlations. **b**, The same streamlines are shown in conjunction with a transparent brain in template space along with delineations that are color-coded by disease. **c**, To derive the cortical topography of dysfunction mappings, smoothed, thresholded and binarized density maps of sweet streamlines were projected onto a brain template in MNI space. Circles show close-up views of disease-wise interconnected cortical sites, anatomically characterized based on the JHU atlas parcellation^68^. Legend of relevant regions, with corresponding JHU atlas denominators in brackets: 1 (JHU: 23 and 24), postcentral gyrus; 2 (1 and 2), superior frontal gyrus (posterior segment); 3 (3 and 4), superior frontal gyrus (prefrontal cortex); 4 (25 and 26), precentral gyrus; 5 (5 and 6), superior frontal gyrus (frontal pole); 6 (9 and 10), middle frontal gyrus (dorsal prefrontal cortex); 7 (17 and 18), lateral fronto-orbital gyrus; 8 (13 and 14), inferior frontal gyrus pars orbitalis; 9 (15 and 16), inferior frontal gyrus pars triangularis.

Second, we mapped optimal stimulation effects to the fronto-subcortical circuitry. Disease-specific data associated different sets of streamlines with optimal symptom improvements (Figs. 4a and 5). Peaks of beneficial DBS networks for DYT primarily interconnected with somatosensory (S1) and primary motor (M1) cortices. Electrode connectivity with M1 and supplementary motor areas (SMAs) emerged as most critical for high stimulation benefit in TS, with premotor regions and SMAs in PD, and with ventromedial prefrontal, dorsal anterior cingulate, dorsolateral prefrontal and orbitofrontal cortices in OCD. Supplementary Fig. 2 displays rotated views of these streamline segregations. Peak voxel coordinates of interconnected cortical sites are summarized in Supplementary Table 9.

Although we consider the visualization of thresholded peaks as most meaningful, thresholding may also mask weaker local optima. Supplementary Fig. 3 provides more comprehensive, unthresholded landscapes of streamlines. Moreover, we quantified statistical certainty per streamline (defined by the −log(*P*) value) to visualize the influence of smaller sample sizes on mappings (Supplementary Fig. 4). As expected, this revealed the lowest certainty for the TS cohort (which had the smallest sample size).

#### Influence of electrode placement

Notably, DBS Fiber Filtering results describe streamlines associated with clinical improvements, which should not be confused with mere electrode connectivity. Indeed, mean implantation sites of the standard (second-to-lowest) DBS contact in DYT, PD and TS resided at negligible distance from each other along the *y* axis of the STN (*P* of all independent two-sample *t*-test comparisons > 0.27, two-sided tests), whereas a significantly different subthalamic aspect was targeted in OCD (all *P* < 0.01, two-sided tests). Hence, we hypothesized the organization of dysfunction mappings to predominantly reflect the stimulation impact on different symptoms rather than mere differences in electrode placement (at least in all disorders but OCD).

To test this assumption further in a data-driven fashion, we implemented a total of three control analyses. First, from the entirety of plain electrode connections per disorder (Fig. 4a, top row), we isolated the subset common to all four disorders and compared it to disease-specific streamline models. Both four-sample and pairwise tests of equality of proportions (two-sided tests) suggested significant differences between proportions of overlap between shared and disease-specific streamlines (Supplementary Table 10).

Second, we mapped sweet spots as three-dimensional Gaussian distributions that were fit to the standard electrode contacts (second-to-lowest) across patients in each disorder. When seeding streamlines from these Gaussians, the resulting streamline profiles expectedly looked much less segregated than the dysfunction mappings achieved by DBS Fiber Filtering. Although, as anticipated, a slight segregation of the OCD bundle emerged given significantly different placement, streamlines seeding from the Gaussians of other disorders were indistinguishable. Furthermore, the OCD streamline bundle was by far not as anteriorly located as in the model that was driven by clinical improvements (Supplementary Fig. 5).

In a final control analysis, we color-coded therapeutic streamlines by a dedicated specificity value. This value was calculated by dividing each streamline’s R value by the average of the R values it had been tagged by within the remaining disorder-specific models. The resulting partitioning among disease-specific streamline bundles highly resembled the one achieved by our ‘conventional’ mapping approach (Supplementary Fig. 6), underlining specificity of dysfunction attributions.

#### Estimation of outcomes based on the model

Using E-field overlap with optimal streamline profiles to estimate clinical improvements of individual patients based on a (circular) in-sample design resulted in significant correlations for all disorders (Fig. 4b). When subjected to fivefold CVs, DYT and PD models were robust across all connectomes but less so for disorders comprising smaller sample sizes (TS and OCD) (Fig. 4b). This is not surprising, because fivefold CVs for models calculated on 14 (TS) or 19 (OCD) patients are prone to failure, by design. Robustness of findings from the larger cohorts, however, make us confident about the general validity of methodological choices. Furthermore, the OCD response bundle identified here has been widely reproduced based on OCD cohorts stimulated to the STN and other subcortical targets (see ref. ^20^ for a review).

#### Model specificity

Despite discernible segregation in dysfunction mappings, disease-wise streamline models, expectedly, also showed considerable overlaps, most visibly among DYT and TS. To quantify the degree of specificity, each profile was, hence, used to cross-estimate outcomes in all remaining disorders. At large, streamline models explained significant amounts of variance uniquely in the disease for which they had initially been calculated (Supplementary Fig. 7).

#### Influence of connectome

Main analyses were informed on a group connectome^21^ calculated on diffusion-weighted magnetic resonance imaging (dMRI)-based tractography of 985 healthy participants of the Human Connectome Project (HCP)^22^. Although representative of average/population brain connectivity, the choice of this particular connectome may bias results. We, thus, repeated our DBS Fiber Filtering analyses using five additional normative connectomes. First, we implemented a connectome of unprecedented spatial (760 µm) and angular resolution based on a single healthy human brain^23^, which optimally lends itself for the detailed visualization of dysfunction mappings (Supplementary Fig. 8b).

A key problem of data-driven whole-brain connectomes (as the two mentioned above), however, is their proneness to false-positive streamlines^24^ and low accuracy in representing small subcortical tracts^25^. To account for this, we repeated our analysis based on a pathway atlas manually curated by expert anatomists^26^ (Supplementary Fig. 8c). Although this dataset is likely the most accurate atlas of subcortical streamlines that currently exists, a potential drawback lies in its proneness to false negatives (because not all fibers of the brain were delineated in this resource). Second, as the atlas is not based on empirical dMRI, small details of streamline trajectories in template space may be misaligned. Thus, we additionally replicated analyses using a pathway atlas informed on population-based fiber tracking combined with expert-defined pathways^26,27^, which we amended with interconnections between the STN and the entire frontal cortex (DBS Tractography Atlas, version 2.1; Supplementary Fig. 8d).

Finally, the generalizability of dysfunction mappings based on normative connectomes to disease-specific alterations remains uncertain. In view of potential therapeutic implications of the identified mappings, we, thus, repeated our DBS Fiber Filtering approach in two exemplary disease-matched group connectomes—one informed on diffusion scans of six patients with OCD (Supplementary Fig. 9a) and one on those of 85 patients with PD^28^ (Supplementary Fig. 9b). A similar rostro-occipital organization emerged across connectomes, with the same order of motor disorders in sensorimotor and premotor cortices toward associative-limbic OCD connections. In-sample correlations and fivefold CVs based on normative and disease-matched connectome models are reported in Fig. 4b and Supplementary Fig. 9c, respectively.

### Retrospective and prospective streamline model validations

**Fig. 6:**
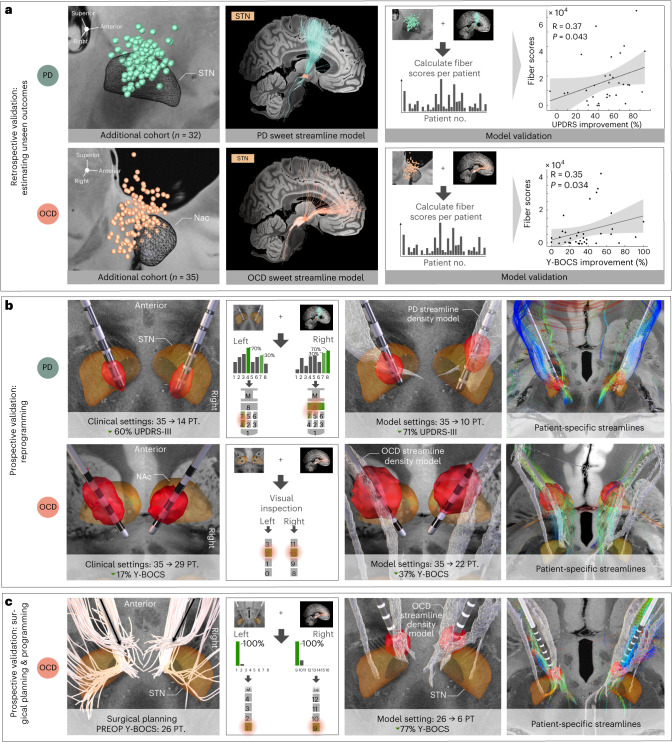
Retrospective and prospective validations of therapeutic streamline targets. To probe the validity of PD and OCD streamline models, five validation experiments were carried out. **a**, First and second, empirical outcomes of two additional independent datasets (PD: *n* = 32 and OCD: *n* = 35) could significantly be estimated based on the degree of overlap of their stimulation volumes with the streamline models. Sweet streamline models calculated on discovery cohorts are represented in disease-specific color, in thresholded and binarized fashion. Model validity is expressed in the form of Spearman’s correlations between the stimulation magnitude by which positive streamlines in the model were respectively modulated (weighted peak 5% of Fiber R scores attributed to each E-field, averaged for bilateral scores per patient) and empirical clinical improvements across the cohort (two-sided tests). Gray shaded areas represent 95% confidence intervals. **b**, Third and fourth, prospective reprogramming was undertaken in two patients. In the patient with PD, directional electrodes had been implanted, so the current was divided using a 70/30% rule based on the contacts with the strongest and second-to-strongest streamline overlaps. This led to an improvement of 71% on the UPDRS-III compared to 60% using clinical settings. In the OCD case, the contact was selected based on visual inspection with the streamline model by the clinical team. This led to a reduction of 37% on the Y-BOCS compared to 17% under clinician-selected parameters. **c**, Fifth, a prospective OCD case underwent streamline-guided DBS surgery. Electrodes were activated at the contact with the highest streamline overlaps (most ventral contacts bilaterally), leading to a rapid Y-BOCS reduction of 77% already 1 month after surgery. Depending on the respective target, reconstructed electrodes and stimulation volumes are featured relative to three-dimensional models of the STN from the DISTAL atlas, version 1.1 (ref. ^28^), or of the nucleus accumbens (Nac) from the California Institute of Technology reinforcement learning (CIT168) atlas, version 1.1 (ref. ^69^), and against anatomical slices of a 100-µm ex vivo brain template^67^. PREOP, pre-operative; PT., points.

Given the potential clinical-translational relevance of identified streamlines in guiding treatment for optimized benefit, we carried out a total of five validation experiments in independent data. We focused on PD and OCD as two substantially distinct brain circuit disorders, because additional retrospective cohorts were unavailable in the remaining two disorders, and no prospective enrollments of patients with these conditions took place at centers associated with this study. First and second, overlaps of stimulation volumes with the respective streamline model (PD or OCD) were used to estimate outcomes in two additional retrospective cohorts. In both the STN-DBS validation patients with PD (R = 0.37, *P* = 0.043) and the VC/VS-DBS validation cohort with OCD (R = 0.35, *P* = 0.034), this procedure corroborated a good fit between estimates and empirical outcomes (Fig. 6a).

Third and fourth, we reprogrammed two individual patients at Würzburg (PD) and Boston (OCD) centers with the intention of maximizing engagement of stimulation volumes with the respective streamline model (Fig. 6b). The first case was a 67-year-old male patient with 9 years into an akinetic-rigid type PD diagnosis who had been implanted to the STN with directional leads. Three months post-operatively, his score of 35 on the UPDRS-III under DBS OFF improved to 14 points (60% reduction) under clinical DBS ON (with medication OFF in both cases). Under streamline-based parameters, symptoms further reduced to 10 points (71% reduction).

The second reprogramming patient from Boston was a 21-year-old female with severe, treatment-resistant OCD characterized by obsessions about food and water intake along with compulsions involving ingestion events and skin picking. Implantation of a conventional omnidirectional lead targeting the VC/VS region led to an improvement of six points (17%) on the Y-BOCS, from 35 points (pre-surgical baseline) to 29 points post-operatively, under clinical stimulation parameters. One month after streamline-based reprogramming, this score further reduced to 22 points (37% reduction).

Fifth, we surgically implanted a pair of subthalamic electrodes for treatment of a 32-year-old male, who had suffered from refractory OCD since the age of 18 years, at the São Paulo center (Fig. 6c). After a depressive phase, the patient had developed a compulsion of noting every word novel to him and transcribing its meaning from dictionaries, by which he filled numerous notebooks. Later, he began experiencing intrusive death-related thoughts that eventually provoked compulsive religious rituals along with concomitant apathy and depression. Since according to the surgical plan, electrode localization had revealed by far the highest overlaps of the ventral-most contacts with the streamline density image; these were activated unipolarly at 3 mA per hemisphere. Only 4 weeks after surgery, the patient, as well as his caregivers, reported a marked improvement of symptoms that had been notable within 1 d after switching on the DBS system. The Y-BOCS score had improved to six points, from a pre-surgical baseline of 26 points (77% reduction).

In all three prospective cases, patient-specific tractography confirmed agreement between individual streamlines and normative models (right-most panels in Fig. 6b,c). Supplementary Table 11 summarizes these prospective patient cases.

### Dysfunction mappings at the level of indirect pathways

Although hyperdirect cortico-subthalamic interconnections are best suited to segregate the frontal cortex, the cortico-basal ganglia-thalamocortical system forms loops that include indirect projections from the striatopallidofugal system (and particularly the external pallidum (GPe)) to the STN^29^. Structural representations of the indirect pathway connecting GPe and STN are organized within Edinger’s comb system^18,30^ and difficult, if not impossible, to reconstruct from diffusion imaging given their orthogonal course to the highly anisotropic internal capsule^26^.

**Fig. 7:**
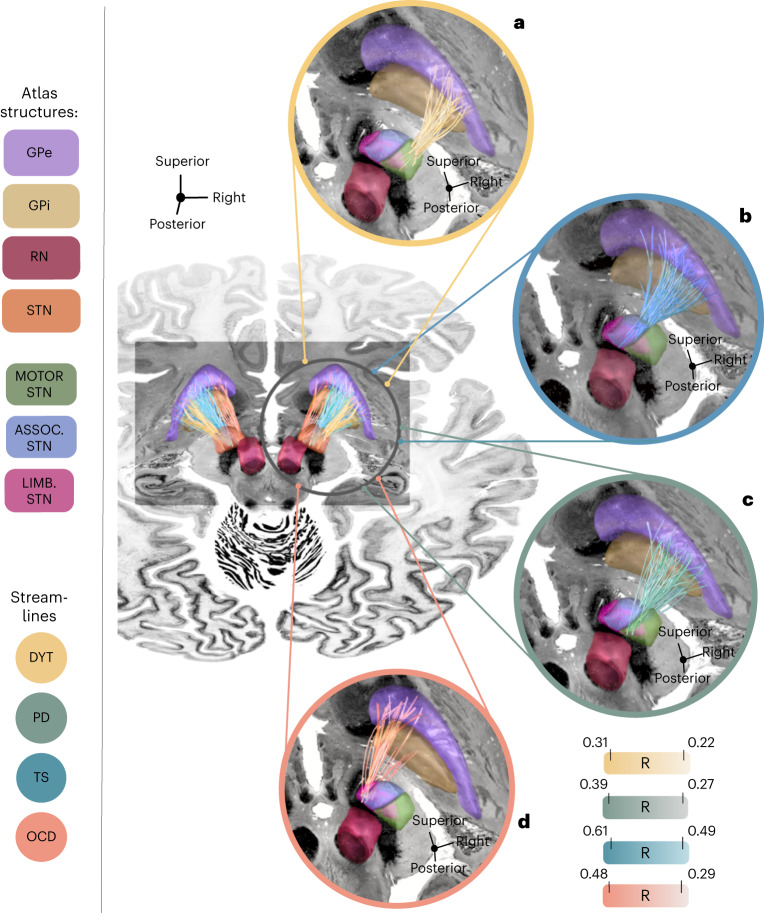
Conserved segregation of dysfunction mappings among indirect pallido-subthalamic connections. Disease-wise sweet streamlines retain a high degree of specificity along their indirect pathway trajectory connecting the STN with the GPi and GPe. Connectivity is modeled based on the Basal Ganglia Pathway Atlas^26^. Sweet streamlines associated with optimal DBS outcomes in DYT (*n* = 56) are interconnected with sensorimotor (**a**), in TS (*n* = 14) with associative (**b**), in PD (*n* = 94) with premotor (**c**) and in OCD (*n* = 19) with limbic (**d**) STN territories. Streamlines are thresholded and represented in disease-specific color. Color intensities attributed to each streamline code for the degree of Spearman’s rank correlation between streamline modulation (peak E-field magnitudes) and clinical outcomes across the disease cohort, with darker colors indicative of higher correlations. Results are displayed relative to several anatomical structures from the DISTAL atlas, version 1.1 (ref. ^28^), and in conjunction with an axial slice (*z* = −10 mm) of the BigBrain template^66^. ASSOC. STN, associative territory of the STN; LIMB. STN, limbic territory of the STN; MOTOR STN, motor territory of the STN; RN, red nucleus.

To interrogate the topography of dysfunction attributions at the level of indirect connections, we, thus, repeated our DBS Fiber Filtering analysis based on pallido-subthalamic streamlines provided by the Basal Ganglia Pathway Atlas^26^. Again, segregation was evident between disease-wise interconnected sites, and their anatomical organization was largely consistent with the one of hyperdirect pathway and sweet spot mappings (Fig. 7). Therapeutic indirect connections in DYT were interconnected with sensory/sensorimotor regions of the STN, whereas those in PD connected to territories within the premotor zone of the nucleus. Interconnected sites in TS predominantly resided within associative aspects and those of OCD within limbic subthalamic areas (insets in Fig. 7).

## Discussion

Derived from 534 invasive brain stimulation sites spanning 11 patient cohorts and three prospective patient cases treated for DYT, TS, PD or OCD across 10 international institutions, we draw three key conclusions. First, we showcase the network effects of intracranial brain stimulation as a viable tool for systematically investigating the coupling between circumscribed cortical circuits and selective clinical dysfunctions. As a method, this approach is capable of mapping what we term the human ‘dysfunctome’—that is, the sets of connections that are disrupted and malfunctioning across different brain disorders. Second, we demonstrate the topographical organization of dysfunction mappings to be mirrored across neuroanatomical levels: (1) among prefronto-subthalamic loops and interconnected cortical sites, (2) within pallido-subthalamic connections and, in miniaturized fashion, (3) within subthalamic subterritories. Dysfunctional networks primarily interconnected the STN with sensorimotor and cerebellar cortices in DYT while involving M1 and SMAs in TS, premotor and supplementary motor areas in PD and ventromedial prefrontal, anterior cingulate, dorsolateral prefrontal and orbitofrontal cortices in OCD. Third, by their association with previous treatment success, these attributions may hold clinical significance as therapeutic targets in stereotactic neurosurgery and non-invasive neuromodulation^1,3^. We present initial retrospective and prospective evidence of applying these results to inform clinical decision-making. Notably, we leveraged the identified circuits to improve treatment benefit in three prospective patient cases.

Methodologically, our study demonstrates the use of subcortical neuromodulation with connectomics as an effective strategy for probing relationships between neuroanatomy and functional impairments. This concept could be perceived as a network-based extension of historical studies that topographically mapped the sites of direct electrical stimulation (often applied cortically during epilepsy surgery) to specific symptoms. Among the most influential authors, Penfield and the scientific team around him assembled an exhaustive functional map of the cortex based on intraoperative mappings of sensorimotor phenomena^31^. The present paper applies a paradigm that may be perceived as a derivative of Penfield’s work, combining brain stimulation administered to a specific small (but widely connected) nucleus deep inside the brain with connectomics. Demonstrating the utility of this approach could pave the way to similar work involving other subcortical and cortical neuromodulation sites. Its application at scale and in an increasingly fine-grained manner (for example, by investigating specific symptoms) may lead to more comprehensive definitions of the human ‘dysfunctome’.

Conceptually, our results identify roles of cortico-basal ganglia pathways in different brain dysfunctions using an invasive method^2^. Attributions were specific to the predominant functional impairment in each disorder. Moreover, they did not reflect merely brain connections of differentially placed electrodes but also their relevance for successful symptom treatment. They were further evident irrespective of which healthy or disease-matched connectomes they had been calculated from. Before the teams at Ann Arbor and Johns Hopkins presented their work on what is now referred to as the Albin–DeLong model^32,33^, the basal ganglia were conceptualized as a funnel that integrates information from different cortical strands to the motor cortex, which then initiates action. In essence, the basal ganglia had primarily been categorized as motor structures. Work by Alexander et al.^8^ challenged this traditional understanding, proposing the idea of parallel circuits involving motor, cognitive and limbic processing. Although there is strong cross-communication at least on cortico-cortical, cortico-striatal, striato-nigral and thalamo-cortical levels, loops associated with different functions retain a degree of segregation throughout their cortico-basal ganglia-thalamo-cortical course^8,34,35^.

The concept of the ‘hyperdirect pathway’ builds on this framework, proposing that certain cortical neurons send direct projections to the STN, which bypass the striatum to create a direct link between the cortex and the structure^6,36^. The functional grouping of subthalamic terminals within these hyperdirect projections can be best understood based on their cortical origins. In this vein, a dorsolateral motor aspect comprising connections to M1 and SMA is defined, along with a ventromedial cognitive territory with origin in superior, middle and inferior prefrontal cortices, and a limbic anteromedial tip connected with orbitofrontal, anterior cingulate and ventromedial prefrontal cortices, hippocampus and amygdala^5,6^. The present results substantiate the general pattern of this distribution based on invasive stimulation sites in four different brain disorders.

Despite confirming a certain amount of segregation between loops, our findings are also compatible with the concept of cross-communication or integration as well as so-called open-loop architectures^6,7^. Indeed, therapeutic targets identified here showed considerable overlaps (most notably between DYT and TS; also see Supplementary Fig. 3). In line with the concept of ‘processing gradients’ (rather than entirely segregated loops), our analysis demonstrates preferential mappings between neuroanatomy and stimulation effects. This notion fits with evidence on partial convergence between terminals from different cortical projection sites and interaction between functional subthalamic subterritories^6^.

Clinically, the identified circuits directly represent therapeutic targets that could inform stereotactic targeting in neurosurgery and potentially non-invasive neuromodulation at the cortical level^1^. This is underscored by the successful retrospective and prospective validations of the OCD and PD streamline targets in the present study, which provide initial evidence for clinical applications of our findings. We must emphasize, however, that the degree of certainty varies between the studied disorders as a function of the sizes of available samples, especially in TS as a relatively novel application for subthalamic DBS with only few implantations performed worldwide.

Sensorimotor and cerebellar loops have been linked to symptom improvements in DYT by investigations that used similar sources of information (such as lesions or different forms of brain stimulation)^18,37^. The sensorimotor cortex along with its basal ganglia interconnections^38^, but also the cerebellum and cerebello-thalamic pathway^39,40^, have been related to dystonic pathophysiology, with non-invasive sensorimotor-cortical^41^ as well as cerebellar^42^ stimulation yielding clinical benefit. Under DBS, aspects of motor control, such as motor sequence learning, voluntary movement coordination, sensorimotor adaptation and the control of specific body parts, may improve, which depend on the accurate functioning of cerebellar or sensorimotor loops (or an integration of both)^43^.

In PD, fronto-subthalamic loops connecting the SMA to the STN have been deemed critical in both historical^44^ and recent work^45,46^. Structural connectivity between subthalamic electrodes and SMA as well as pre-motor areas correlated with motor improvements in PD, and overlap of stimulation volumes with this set of streamlines, was associated with outcomes in independent patients^45^. This is in line with PD motor benefit observed under cortical SMA stimulation^47^. Functionally, these therapeutic effects may relate to involvement of the SMA in movement selection, preparation and initiation^48^.

The streamline bundle identified in the OCD cohort emerged as an effective ‘OCD response streamline target’ beyond stimulation of the STN region (for a review, see ref. ^20^). Conceptualized as an associative-limbic hyperdirect pathway with passage through the internal capsule on its trajectory toward the STN and other mesencephalic nuclei, this bundle is connected to diverse prefronto-cortical regions, such as the anterior cingulate and dorsolateral prefrontal cortex^5,6^. These findings were also confirmed via functional mappings^20,49^. Concurrently, these cortical sites have been FDA approved as transcranial neuromodulation targets for OCD^50^. Functionally, recalibrating this streamline bundle may resolve repetitive thoughts and behavior by interrupting a pathological control signal emitted through hyperactive prefrontal regions^20^.

Network correlates to restore functionality in TS are less established, especially not via the more recent application of subthalamic DBS^16,17^. Here, the most highly weighted hyperdirect streamlines showed connectivity to M1 and SMA. These regions align with tic-related alterations^51,52^, have been associated with tic reduction under thalamic^53^ or pallidal DBS^54,55^ and have been probed as non-invasive neuromodulation targets for TS^56^. The SMA, in interaction with M1, may be involved in tic preparation, relaying signals to areas underpinning action monitoring or tic execution^57^.

Our study has several limitations. First, analyses relied mainly on retrospective data, which may bias the interpretation of clinical outcomes. Aggregation of a large multi-center sample (*n* = 534 electrodes) further inevitably introduced different sources of variability. Still, most of our models extrapolated across differences in targeting strategies between surgeons and centers, imaging modalities and protocols, electrode models, stimulation paradigms or clinical assessment strategies. Furthermore, we validated results on unseen cohorts and prospectively tested PD and OCD circuits in individual patient cases.

Second, the underlying physiological effect may not be fully captured by the simplified biophysical model employed here to approximate the amount of tissue activated. For instance, E-field models neglect the impact of stimulation onto glial cells, intracellular processes or synaptic reorganization^58^. In addition, varying stimulation parameters may entail differential consequences^59^. For these reasons, refined modeling of the focal stimulation impact might contribute to a further increased validity of results^25^.

Third, warping lead localizations into template space may have introduced slight mismatches. We sought to counteract this bias as much as possible by using an advanced processing pipeline that included brain shift correction^60^, multi-spectral normalization, subcortical refinements and phantom-validated electrode reconstructions^61^. We further applied a normalization strategy with similar performance in STN segmentation as manual delineations by anatomical experts in two independent evaluations^62,63^. In addition to meticulous visual inspection and refinement of all remaining outputs of this processing pipeline, the subthalamic atlas fit was manually optimized via the WarpDrive toolbox^64^.

Fourth, with patient-specific dMRI largely unavailable, anatomical delineations were based on normative or disease-matched connectivity. Undoubtedly, the use of connectivity acquired outside of the patient sample in question introduces limitations regarding the anatomical accuracy. Nevertheless, an advantage of normative connectomes lies in their higher resolution and signal-to-noise ratio than what would be attainable during clinical routine. This unprecedented quality results from the possibility of longer scanning durations and reduced movement artifacts in healthy participants as compared to movement disorder and neuropsychiatric patient populations. Similarly, the frequent use of advanced acquisition tools critically contributes to increased imaging quality. At the same time, our study was precisely aimed at deriving a ‘broad lens’ description of dysfunctional networks in the average human brain, and mappings were largely consistent across four normative connectomes. Additionally, we demonstrated the validity of segregations in the face of disease-specific connectivity alterations. Although here and in previous research (for a review, see ref. ^1^) normative models could significantly account for variance in clinical outcome outside of the discovery sample and showed prospective clinical benefit, the reported streamlines should be further validated in patient-specific data before clinical application in stereotactic targeting and programming of DBS systems.

Finally, cohorts of two disorders (OCD and TS) analyzed in the present study were similarly small, and most of the resulting models did not survive CVs. The same methodology was applied to all disorders, and, hence, the approach itself could be validated based on the remaining two disorders (PD and DYT). We further validated the OCD streamline model using an additional patient cohort and two prospective cases. Concurrently, the same bundle has been described^21,65^ and validated repeatedly in earlier work to be relevant for OCD (for a review, see ref. ^20^). However, especially for TS, the limitation of a low *n* still stands.

In conclusion, our study demonstrates the potential of invasive brain stimulation as a ‘flashlight’ pointing from the subcortex onto the topography of the human ‘dysfunctome’. Specifically, we found beneficial stimulation effects in four different brain disorders to be organized as a function of cardinal symptom domain at the level of fronto-subthalamic circuits and their interconnected cortical sites. Intriguingly, a comparable—yet miniaturized—topography was mirrored focally at the site of stimulation in the subcortex. This scaling effect on dysfunction attributions across neuroanatomical levels provides a compelling answer to the conundrum of similar clinical effects after stimulation to different access nodes of a shared therapeutic network.

## Methods

### Patient cohorts, imaging and clinical assessments

Every stage of the research process complied with all relevant ethical regulations, and post hoc analyses performed for the purpose of the present manuscript were approved by the institutional review board of Charité – Universitätsmedizin Berlin (master vote EA2/186/18). Procedures of clinical trials and studies leading to the collection of these data were approved by the institutional review boards at each of the respective data collection sites. They were carried out in accordance with the 1975 Declaration of Helsinki, and all participants signed an informed consent before study participation. Participants received no compensation in exchange for taking part in this research.

#### Discovery cohort

The present study sought to establish models of optimal focal stimulation sites and streamlines, harnessing a retrospective discovery sample of eight patient cohorts (*n* = 197) spanning seven international DBS centers (San Francisco, Shanghai, Berlin, Würzburg, Grenoble, London and Pisa/Milan). Each of these patients had been bilaterally implanted with subthalamic DBS for treatment of DYT (*n* = 70), PD (*n* = 94), TS (*n* = 14) or OCD (*n* = 19). The full sample consisted of two patient cohorts per disease, with the Shanghai center contributing two cohorts (DYT and TS data). Among all available patients with complete neuroimaging and clinical outcome information, no patient was discarded from our analyses. Instead of prospective randomization, we leveraged incidental variability in electrode placement within each disease cohort, which can be presumed to be random. Supplementary Table 1 summarizes the included discovery cohorts, with more detailed patient-wise demographic and clinical information listed in Supplementary Tables 2–5.

Given the exploratory nature of our study, no statistical methods were used to pre-determine sample sizes. As neuropsychiatric applications of STN-DBS are recent and rare, samples, especially in TS and OCD, are limited by the small number of worldwide surgeries. In the TS cohort, we included all globally treated patients undergoing STN stimulation for this condition at the time of analysis. Overall, we were able to include two cohorts per disease group. Our initial assumption of expected effect sizes was based on Li et al.^21^ and Treu et al.^70^, with an R of approximately 0.4 for reported correlations between empirical clinical outcomes and estimated gain scores. In view of the natural restrictions in available sample sizes, we calculated a ‘compromise’ type power analysis using G*Power, version 3.1.9.6 (refs. ^71,72^), to determine the power of our analyses based on the accessible data per disorder to detect the assumed effect. Given a β/α ratio of 1, the available DYT sample (*n* = 56 in the main cohort) used for the model setup was powered to 0.94, the PD sample (*n* = 94) to 0.98, the TS sample (*n* = 14) to 0.77 and the OCD sample (*n* = 19) to 0.81 for detecting the assumed effect size. To our knowledge, this is the largest transdiagnostic study of its kind.

#### Retrospective validation cohorts

To further validate streamline models in two exemplary disorders based on out-of-sample data, two additional patient cohorts were integrated. The first consisted of a further cohort of patients with PD from Würzburg receiving STN-DBS (*n* = 32). The second comprised an additional cohort of patients with OCD, pooled across London, Cologne and Boston centers, treated with DBS of the VC/VS region (*n* = 35). Crucially, these patients contributed entirely independent data points that had not been used to inform the previous streamline model setup. The only exception was formed by the OCD-DBS cohort from London, in which patients had received a set of electrodes each to both targets (STN and VC/VS, with *n* = 4 electrodes per patient) that had been activated independently during the original study^73^. For this cohort, stimulation settings and clinical scores with ‘optimized’ stimulation of both targets combined or of each target separately were available. For model generation within the discovery cohort (with subthalamic focus), stimulation parameters and corresponding Y-BOCS improvement values collected during the ‘STN-DBS-only’ phase were implemented, whereas corresponding information acquired during the ‘VC/VS-DBS-only’ phase was used to inform the retrospective OCD model validation. Supplementary Table 6 summarizes these two additional retrospective cohorts. Patient-specific information is provided in Supplementary Table 7 (PD validation cohort) and Supplementary Table 8 (OCD validation cohorts). Again, none of the available patients with complete neuroimaging and clinical information was excluded from further analysis.

#### Prospective patient cases

Streamline models for PD and OCD were further prospectively validated by reprogramming DBS settings in a patient with PD and in a patient with OCD from Würzburg and Boston, respectively, guided by the aim of maximized engagement of their stimulation volumes with the corresponding streamline model. Both patients were recruited and investigated within the ongoing clinical service—namely, in the inpatient DBS program at University Hospital Würzburg in the case of the patient with PD or in the DBS program of the psychiatry and neurosurgery departments at Massachusetts General Hospital (MGH) in the case of the patient with OCD.

Finally, a single patient with OCD from São Paulo underwent DBS surgery and programming as informed by the OCD streamline model. This patient was recruited within the regular surgical service of Clínica de Dor e Funcional after classification as a refractory case of OCD that was associated with depression. He underwent evaluation by a neurologist, a psychologist and two functional neurosurgeons before approval of the DBS implantation surgery by the Ethics Board Committee of the State of Rio Grande do Sul. Supplementary Table 11 provides additional details on these three patient cases.

To inform surgical planning and for exclusion of structural abnormalities, all patients received high-resolution multi-spectral structural MRI that had been acquired at 3T field strength. High imaging quality was ensured through visual inspection by a multi-disciplinary team during stereotactic planning, and, in case of movement artifacts, pre-operative acquisitions were repeated under general anesthesia. Intra-operative microelectrode recordings and macrostimulations as well as either post-operative MRI (*n* = 73) or computed tomography (CT) of the head (*n* = 188) (Supplementary Tables 1–8 and 11) were acquired to confirm accurate lead placement.

Specifics on electrode models implanted in each cohort used for the model setup are summarized in Supplementary Table 1, and the same information for retrospective model validation cohorts and prospective patient cases is provided in Supplementary Tables 6 and 11, respectively. Stimulation settings and corresponding clinical improvement scores for all cohorts were selected from times of follow-up to which stimulation effects had sufficiently stabilized (Supplementary Tables 1, 6 and 11).

Times of follow-up available for some patients within the *n* = 58 cohort of patients with DYT from Shanghai were shorter than those of other disease cohorts. In addition, as DYT is a heterogeneous disease of several forms (for example, generalized, segmental and focal somatotopic expressions), pre-operative BFMDRS summary scores in some Shanghai patients were considerably lower than those of patients in the San Francisco cohort. To ascertain stabilized and comparable DBS effects across cohorts, main analyses were, thus, carried out on the DYT sample including a subcohort of Shanghai patients (*n* = 44), which sufficed to more conservative inclusion criteria (baseline BFMDRS scores ≥5 and follow-up ≥6 months). However, we repeated our results on the complete DYT sample (*n* = 70) including the full Shanghai cohort (*n* = 58) to demonstrate stability of effects (Supplementary Fig. 10).

Clinical outcome data and DBS parameters were retrieved from the collecting sites using Microsoft Excel, version 16.70, and imported for analysis using MATLAB R2022b, version 9.13.0.2105380 (MathWorks). Clinical improvement was measured in the form of relative change from pre-operative baseline to post-operative follow-up under DBS ON (or from post-operative OFF to ON DBS conditions in the case of PD) within the primary outcome assessment of each disease cohort: BFMDRS in DYT, UPDRS-III in PD, Y-BOCS in OCD and YGTSS in TS. Blinding was not relevant in the case of the secondary analyses of existing datasets performed here. To mitigate the risk of observer bias, we tested the explanatory value of our models in hold-out data and performed retrospective and prospective validation experiments (see below).

Of note, most statistical results in the present manuscript involve Spearman’s rank correlations, which do not make assumptions about the underlying distribution. In addition, for these results, scatter plots of individual data points are shown. For analyses in which *t*-tests were calculated, normality and equality of variances were formally tested (and present for all cases).

### DBS electrode localization and E-field modeling

DBS electrodes of all patients were localized based on default settings in an advanced, state-of-the-art processing pipeline as implemented in Lead-DBS software, version 3.0 (https://www.lead-dbs.org)^74^. MATLAB R2022b, version 9.13.0.2105380, was used to apply this Lead-DBS-based analysis stream. In brief, our approach involved linear co-registrations of post-operative head CT or MRI scans to pre-operative T1-weighted images by means of Advanced Normalization Tools (ANTs; http://stnava.github.io/ANTs/)^75^. Co-registration results were subsequently corrected for potential intra-operative brain shift via an automatized subcortical refinement module (as implemented in Lead-DBS) but also needed to conform to meticulous visual inspection by two expert users (B.H. and N.L.). This latter step led to manual refinement in cases where aberrations were detected.

All pre-operative acquisitions were used for multi-spectral spatial normalization into ICBM 2009b Nonlinear Asymmetric (‘MNI’) template space^76^ using the Symmetric Normalization (SyN) approach included in ANTs with the ‘effective: low variance + subcortical refinement’ preset in Lead-DBS. This method had outperformed similar approaches for subcortical normalizations (including STN segmentation) across >10,000 nonlinear warps and different normalization techniques in two independent studies, with precision approaching manual expert segmentation^62,63^. For all analyses and visualizations of results, atlas definitions of the STN were based on the DBS Intrinsic Template (DISTAL) atlas, version 1.1 (ref. ^28^), a precise subcortical atlas explicitly created for use within Lead-DBS and based on convergent information from multi-modal MRI, histology and structural connectivity.

To maximize registration accuracy further, normalization warp fields were manually refined using the WarpDrive^64^ toolbox included in Lead-DBS, version 3.0 (ref. ^74^), wherever mismatches in the registration were clearly visible and with particular attention to the STN as the anatomical structure in focus. Although high registration precision between a template brain and individual brain anatomy is of utmost importance for accurate reconstructions of electrode localizations, the low contrast of basal ganglia structures on typically applied clinical imaging sequences renders the automated registration between an individual and an atlas STN challenging^62,77^. WarpDrive is conceived as a dedicated but optional module that allows to manually counteract small misalignments after automated normalization has been performed. Supplementary Fig. 11 shows examples of optimized normalization warp fields after manual WarpDrive refinements (ANTs + WarpDrive) in head-to-head comparison to unrefined, direct results of the automated pipeline (ANTs only). Across the entire discovery cohort, displacements of 0–1 mm were applied in *n* = 296 electrodes, of 1–2 mm in *n* = 82 electrodes and of >2 mm in *n* = 16 electrodes.

Subsequently, electrodes were pre-localized using the phantom-validated Precise and Convenient Electrode Reconstruction for Deep Brain Stimulation (PaCER) algorithm^61^ in the case of post-operative CT. In the case of post-operative MRI, the trajectory search/contact reconstructions (TRAC/CORE) algorithm^78^ was implemented instead, both as included in Lead-DBS, version 3.0 (ref. ^74^). The resulting pre-localizations were visually inspected and manually refined by two experienced users (B.H. and N.L.).

Integrating patient-specific active electrode contacts with corresponding stimulation parameters, the E-field as the gradient distribution of electrical potential in space was simulated in native patient space via an adaptation of the SimBio/FieldTrip pipeline (https://www.mrt.uni-jena.de/simbio/ and http://fieldtriptoolbox.org/)^79^ as implemented in Lead-DBS, version 3.0 (ref. ^74^). Using a finite element (FEM) approach, a volume conductor model was created on the basis of a four-compartment mesh^60^, which involves a realistic three-dimensional model of electrodes (metal and insulating electrode aspects) and surrounding anatomy (gray and white matter). Again, gray matter was defined using the DISTAL atlas, version 1.1 (ref. ^28^). Finally, electrodes and E-fields were transformed into template space based on the (manually optimized) warp fields priorly determined during normalization of pre-operative MRI acquisitions. These steps allowed for visualization and analysis of electrodes and stimulation fields at the group level using the Lead-Group toolbox^70^ as well as DBS Sweet Spot and Fiber Filtering Explorers^74^.

### Dysfunction mappings at the subthalamic level

Model definition (Fig. 1a). Our group-level approach intended to delineate and compare the organization of disorder-specific stimulation effects across different neuroanatomical levels, namely (1) that of the subthalamic target site (DBS Sweet Spot Mapping) as well as (2) that of fronto-subthalamic pathways and interconnected cortical sites (DBS Fiber Filtering).

In the first part of our analysis stream, DBS Sweet Spot Mapping^18^ (Fig. 1a) was performed in each disease cohort separately to identify subthalamic voxels linked to optimal stimulation-related improvements within each respective cardinal dysfunction. For this purpose, information from patient-specific E-fields was integrated with corresponding clinical outcome scores. The E-field denotes the first derivative of the estimated voltage distribution administered to voxels in space, exhibiting greater intensity near active electrode contacts and diminishing rapidly as distance increases. On a voxel-by-voxel basis, the E-field magnitude in each voxel encompassed by the E-field volume was denoted for each patient across the cohort. To account for variability in voxels covered across E-fields within each cohort and to circumvent unrepresentative results biased by too few data points, the region of interest (ROI) was limited to brain voxels encompassed by at least 50% of E-fields exceeding a magnitude threshold of 200 V/m. This E-field magnitude corresponds to a commonly assumed estimate of voltage needed to activate axons^45,80,81^ and has been repeatedly successfully applied in models of DBS effects on anatomy surrounding the active electrode contact across disorders (for example, in refs. ^19,82^). Nonetheless, sweet spot modeling and corresponding quantitative validations were repeated for a range of different thresholds (that is, 180 V/m, 200 V/m and 220 V/m) to demonstrate robustness of results (Supplementary Fig. 1). For each considered voxel, this procedure resulted in a vector of E-field magnitude values of the length of the respective patient sample.

Iterating through brain voxels encompassed by the group of thresholded E-fields in template space, Spearman’s rank correlations were calculated between the vector of E-field magnitudes and the vector of relative clinical improvements of all patients. This procedure resulted in a map of positive peak voxels associated with beneficial stimulation effects (sweet spot) as well as negative peak voxels related to detrimental stimulation effects (sour spot). The resulting model can be conceived as an optimal map of where E-fields should ideally stimulate the focal anatomy to maximize treatment success within the respective domain of dysfunction. Of note, these correlation coefficients should not be interpreted as significant results due to the mass-univariate (voxel-wise) nature of our analysis. Instead, they were validated by probing model performance in estimating clinical outcomes in a fivefold CV design (see below).

#### Estimation of outcomes based on the model

Once optimal sweet spot models had been established in each disease cohort, each model was tested for its explanatory value for clinical outcome variance. To do so, magnitudes of individual E-fields were multiplied with the model in a voxel-wise fashion, and results were averaged across voxels. This led to the attribution of one ‘Sweet Spot Score’ per E-field, and scores were finally averaged across bilateral E-fields to achieve one single Sweet Spot Score per patient. Our modeling approach followed the logic that E-fields in which peaks spatially overlapped highly with the sweet spot (receiving a high positive Sweet Spot Score) would be associated with considerable clinical improvements, whereas E-field peaks markedly encompassing the sour spot (high negative Sweet Spot Score) would result in low or negative estimates. To probe the tenability of this hypothesis, we performed in-sample Spearmanʼs correlations (two-sided tests) between Sweet Spot Scores and empirical clinical outcomes across the cohort. More precisely, the model was calculated on the full discovery cohort, and E-field overlap with it was used to estimate clinical outcomes in each discovery patient. Although representing circular outcomes, these in-sample correlations allowed to compare results (1) across disorders and (2) between sweet spot and sweet streamline findings.

To investigate the generalizability of these results, we further tested whether disease-wise models were robust when subjected to a fivefold CV design, where the sweet spot model was built on a subset of four-fifths of the respective disorder’s discovery cohort (training set) in each of the five folds, and results were used to estimate clinical outcome of the remaining (held-out) fifth of patients (test set). Crucially, because data of remaining patients were not used to inform the model, respectively, the CV strategy was unbiased by circularity. Once estimates for all patients had been derived, the sweet spot model accuracy was finally tested by correlating model-based Sweet Spot Scores with empirical outcomes across the disease cohort. In all analyses, *P* values were derived based on permuted testing building on 5,000 iterations.

#### Visualization of subthalamic dysfunction mappings

Disease-wise sweet spots were smoothed by a kernel of two at full width at half maximum using Statistical Parametric Mapping (SPM12) software (https://www.fil.ion.ucl.ac.uk/spm/) to visualize the organizational pattern of subthalamic dysfunction mappings across disorders. Smoothed profiles were projected onto the surface of a three-dimensional model of the STN in ICBM 2009b Nonlinear Asymmetric space derived from the DISTAL atlas, version 1.1 (ref. ^28^), using Surf Ice software, version 1.0.20211006 (https://www.nitrc.org/projects/surfice). Three-dimensional density plot renderings of sweet spots were further generated by plotting R value magnitudes coded by spheres with different sizes and alpha values in space using Lead-DBS, version 3.0 (ref. ^74^). Namely, the size and alpha value (transparency) of spheres were weighted by the correlation of modulating the coordinate with clinical outcomes, so that higher correlation results were visualized in the form of larger and less transparent spheres. Two-dimensional axial and coronal views of sweet and sour spots were additionally displayed separately for each disorder using 3D Slicer software, version 5.2.1 (https://www.slicer.org/).

### Dysfunction mappings at streamline and cortical levels

#### Model definition (Fig. 1b)

The second part of our analysis stream followed the intention of deriving the topographical organization of dysfunction mappings (1) at the level of hyperdirect fronto-subthalamic streamlines and (2) that of interconnected sites within the frontal cortex.

To understand the relationship between DBS-based modulation of specific streamlines and a given clinical effect, we, thus, harnessed a previously validated structural connectivity analysis, termed DBS Fiber Filtering^65^ (Fig. 1b), in an adapted form for implementation in (non-binarized) E-fields^19^. Structural connectivity was primarily defined by a population-based group connectome derived from multi-shell dMRI-based tractography data of 985 healthy participants acquired within the HCP 1,200 subjects release^22^. Details on the calculation procedure of this connectome are reported in Li et al.^21^. In brief, computation of a whole-brain connectome for each of the 985 healthy patients was performed using the Lead-Connectome tool, as provided within the Lead-DBS environment, version 2.0 (ref. ^60^) (https://www.lead-dbs.org/about/lead-connectome/). Normalization of streamlines into template space involved a multi-spectral warp building on T1-weighted, T2-weighted and diffusion-weighted acquisitions through use of ANTs (using the ‘Effective Low Variance’ preset in Lead-DBS, version 2.0 (ref. ^60^)). In total, 6,000 streamlines were sampled per individual, which were finally aggregated across all 985 HCP participants to form a collective dataset in MNI space (encompassing a total of 6,000,000 fibers).

Although, by design, normative connectivity is unable to fully account for patient-specific anatomical variability, it is optimally suited for ‘broad lens’ insight into the average human brain at particularly high resolution, as precisely aimed at in the present investigation. Although small inter-individual differences in the topography of human fronto-subcortical interconnections exist, at least a general agreement can be presumed (Supplementary Fig. 12). To assess the general topography of cortico-subthalamic interconnections, we seeded streamlines from five seed points that were manually placed along the dorsal convex shape of the STN in standard space (Supplementary Fig. 12a). We normalized streamlines originating from nine random individual HCP subjects and selected streamlines that connected to each of the seed points in individualized connectomes (Supplementary Fig. 12b). This enabled visual comparability between the topography of STN connections. Although, as expected, the general fronto-rostral topography of interconnections between cortex and STN was revealed in all nine individuals (Supplementary Fig. 12c), the results also show individual variance. In this context, we must mention that only an unknown fraction of these differences should be attributed to true anatomical variance. As demonstrated by several authors using test–retest analysis of brains that were scanned multiple times with dMRI, a substantial fraction of individual differences needs to be attributed to noise and distortions in the data, the choice of MRI machine and tractography algorithms^83,84^.

Again, the streamline modeling procedure was performed in each disease cohort separately. Per disorder-wise cohort, we first isolated the subset of streamlines from the normative connectome that passed in proximity of at least a minimal number of electrodes. These were characterized in the form of streamlines traversing a rather high E-field magnitude (>0.8 V/mm) close to active contacts in more than 0.5% of E-fields within that cohort. Iterating through this subset of streamlines, one streamline at a time, the stimulation impact per E-field on each streamline was estimated by the peak value among E-field magnitudes collected from points along its passage. This resulted in a ‘streamlines by E-field peaks’ matrix in which each entry denoted the peak impact of each E-field on each streamline.

Second, the entries of the ‘streamlines by E-field peaks‘ matrix were Spearman’s rank correlated with clinical improvements across the disease cohort. Following this procedure, each streamline was tagged by an R value coding for the association strength of its modulation with clinical outcome. The resulting streamline profile can be seen as a model of optimal connectivity for maximal clinical improvements, where streamlines with positive weights would be strongly modulated by E-fields of good performers (sweet streamlines) and such with negative weights by E-fields of poor performers (sour streamlines). As these correlation coefficients relied on a mass-univariate approach, streamline profiles were later validated by probing their capability to estimate clinical improvement in data that had not been used to inform the model (see below).

#### Estimation of outcomes based on the model

To determine how well disease-wise optimal sweet streamline profiles would perform in estimating clinical improvement in single patients, the peaks of their E-fields were overlapped with the streamline model of optimal electrode connectivity. Specifically, streamlines from the model touched by that E-field were first isolated. Iterating through this subset, the R value of each streamline was subsequently multiplied by the peak E-field magnitude to account for the strength of its modulation by this E-field. Moreover, only the peak 5% of these weighted sweet streamline R values were retained to maximally account for the streamlines most impacted on by that E-field. These top 5% of weighted R values were then summed up, so that each E-field was tagged by the ‘weighted peak 5% of Fiber R scores’. Because each patient within the cohorts considered in the present study had been implanted to the STN at both hemispheres, the Fiber R score was finally averaged across bilateral E-fields to result in one single value per patient.

Following the logic of this procedure, E-field peaks displaying high overlaps with beneficial streamlines would receive high clinical scores, whereas those with no (or merely peripheral) overlap would receive low clinical estimates. A subset of the most relevant sweet streamlines was selected for this validation step using an R value threshold set at the top 1% of the cumulative distribution function of R values of all streamlines. This pre-selection step included such streamlines where high modulation had led to high improvement (with high meaningfulness for the model) while discarding potentially less relevant or noisy correlations.

Model validation within the discovery cohort followed a similar strategy as implemented in the case of Sweet Spot Mapping. Specifically, we tested for an association between spatial overlap of a patient’s E-fields with the model (weighted peak 5% of Fiber R scores) with empirical clinical improvements across patients using Spearmanʼs correlation (two-sided test). In doing so, the ability of streamline models to explain in-sample variance could be scrutinized for comparability of results (1) across disorders and (2) with sweet spots. For in-sample analyses, sweet streamline models were derived based on E-fields of all patients within each disease cohort and validated using each patient of the respective sample (circular analysis). Ultimately, all models were subjected to CVs in a fivefold design to investigate the generalizability of their explanatory value in hold-out data. Again, this approach was implemented by randomly splitting each disorder’s discovery patient cohort into five folds. The disease-specific model was built on four-fifths of patients and validated on the remaining fifth. This strategy was repeated five times, so that model-based estimates (weighted peak 5% of Fiber R scores) were obtained for all patients across all folds. These estimates could then be correlated with empirical clinical outcomes to evaluate model validity in a non-circular fashion. Again, *P* values were calculated using permutation testing based on 5,000 iterations.

#### Visualization of cortical dysfunction mappings

To elucidate the topographical organization of interconnected fronto-cortical regions, disease-wise sets of sweet streamlines were first converted to voxelized images (streamline density maps). The resulting maps were then smoothed using an 8-mm Gaussian kernel at full width at half maximum as implemented in SPM12 (https://www.fil.ion.ucl.ac.uk/spm/) and projected onto the cortical surface of the MNI template using Surf Ice software, version 1.0.20211006 (https://www.nitrc.org/projects/surfice). Anatomical correlates of disease-wise cortical sites interconnected with sweet streamlines were then defined based on the Johns Hopkins University (JHU) atlas parcellation^68^.

#### Quantification of spatial uncertainty

Furthermore, we aimed to quantify and visualize the degree of spatial uncertainty per streamline within disorder-wise dysfunction mappings at the streamline level. For this purpose, the thickness of each streamline was determined by the −log(*P*) value, meaning that thicker streamlines would be illustrative of lower *P* values.

#### Influence of electrode placement

Subsequently, we intended to scrutinize the relative impact of different model inputs. Besides the choice of a normative connectome, DBS Fiber Filtering results are determined mainly by two major sources of variability across patients—namely, (1) by the precise placement of the stimulation volume and (2) by clinical improvements. In three out of the four disorders of interest in the present study (DYT, PD and TS), stereotactic targeting aims at the same site within the dorsolateral aspect of the STN, whereas the OCD target resides more antero-medially. Thus, the partitioning of dysfunction mappings among various disorders could predominantly be driven by the stimulation impact on clinical outcomes and may not rely solely on differential electrode placement.

To investigate this hypothesis, we implemented a total of three data-driven control analyses. First, plain streamline connections seeding from bilateral stimulation volumes were isolated for each disorder. These comprised the entirety of structural connections activated by a bilateral E-field, irrespective of the importance of their modulation for clinical outcome. Among these, only the subset of streamlines shared across disorders was retained and contrasted to disease-specific sweet streamlines. Four-sample and pairwise tests for equality of proportions (two-sided tests) were performed to compare the degree of overlap between them.

Second, we fit a three-dimensional Gaussian distribution to the standard (second-to-lowest) electrode contacts of all patients with a specific disorder, leading to four blurred volumes within the STN. Streamlines were then seeded from each of these Gaussians as ROIs. The partitioning among the resulting disease-wise connectivity profiles was consequently visually compared to the streamline segregation model that had been achieved using DBS Fiber Filtering. In the latter approach, streamlines connected to empirical stimulation volumes of patients had been weighted by stimulation-related outcomes within the four different domains of dysfunction.

Third, each connection within each respective sweet streamline model per disorder was color-coded by a specificity value, which was calculated by dividing its R value by the average of R values that it received across the three remaining disorders. The streamline segregation result of this approach was finally visually compared to that of the connectivity profile that had been established based on ‘conventional’ color-coding as informed by a streamline’s unbiased R value (resulting from our DBS Fiber Filtering analysis).

#### Model specificity

Besides validation of each model within the respective disorder that it had been calculated on, we were interested in the degree of specificity of disease-wise models in their ability of explaining clinical outcome variance. To demonstrate specificity, we considered the sweet streamline models for each disorder and overlapped E-fields of patients in all remaining three disorders with the model to predict clinical outcomes in a disorder-by-disorder fashion. Details of this cross-prediction approach were equivalent to those of the CV strategy described above. In brief, weighted peak 5% of Fiber R scores were estimated for each patient based on the degree to which their E-fields encompassed the model, and, finally, Spearmanʼs correlations were performed between these estimates and the empirical clinical outcomes across the cohort of patients to test for model accuracy. In the case of specificity of dysfunction mappings, each of the models would show predictive utility uniquely for clinical improvements within the corresponding outcome measure (good fit between estimates and outcomes) but not for those of other clinical scales (poor fit).

#### Influence of choice of connectome

Furthermore, we aimed to scrutinize the influence imposed by a particular normative resource chosen to inform connectivity in our DBS Fiber Filtering analyses. To do so, we repeated modeling and model validation procedures using five additional connectomes based on otherwise equivalent model parameters. The first such resource consisted of a normative whole-brain connectome, derived from a multi-shell diffusion-weighted imaging dataset at 760-µm isotropic diffusion acquired in vivo in a single healthy participant over a total duration of 18 scanning hours^23^ (MGH Single Subject 760 µm Connectome; openly available from https://datadryad.org/stash/dataset/doi:10.5061/dryad.nzs7h44q2). Although generalizability of results derived using this connectome to a larger population is naturally limited by its single-patient origin, it lends itself particularly well for detailed anatomical insight and visualization by dint of its unprecedented imaging resolution.

Second, an axonal pathway atlas^26^ (Basal Ganglia Pathway Atlas; openly available from https://osf.io/mhd4z/) was implemented, which did not rely on tracking of streamlines based on dMRI data and, thus, circumvents some of the most important drawbacks of dMRI-based tractography (such as the possibility of integrating false-positive connections)^24^. Instead, streamlines included in this tractogram were manually defined by expert anatomists within an advanced augmented reality (holography) framework. Guided by control points, this technique allows for precise localization and reconstruction of basal ganglia anatomy aided by three-dimensional images created from laser beams. Although the expert-characterized nature of this resource ensures a highly accurate representation of empirically existing (true-positive) connections, it is limited by a higher degree of false-negative streamlines (as the focus in its creation by the expert anatomists lies in accuracy at the expense of exhaustiveness).

Third, we employed a custom-made pathway atlas (DBS Tractography Atlas, version 2.1; openly available from https://github.com/netstim/DBS-Tractography-Atlas.git) informed on previously defined pathway atlases, including the DBS Tractography Atlas, version 1 (ref. ^27^), and the aforementioned Basal Ganglia Pathway Atlas^26^. It was completed by additional streamline tracking with focus on a comprehensive description of subthalamic interconnections with multiple cortical and subcortical nodes, leading to a finite set of 6,525,876 streamlines. Its creation specifically followed the intention of representing streamlines that had previously lacked delineation in other resources.

To generate this atlas, a first subset of streamlines connecting the STN to different cortical regions was derived via streamline tracking based on the HCP-1,065 diffusion data, which scanned 1,065 young and healthy adults^85^. These data are openly available within DSI-Studio (https://brain.labsolver.org/hcp_template.html; fiber orientation maps at 1-mm resolution). Using DSI-Studio (https://dsi-studio.labsolver.org/), the STN as defined within the DISTAL atlas, version 1.1 (ref. ^28^), was specified as an end region, and 1,500 streamlines were tracked from each of nine cortical Brodmann areas (BAs) as ROIs from the digitized Brodmann atlas^86^. These comprised BA1/2/3 (primary and secondary somatosensory cortex), BA4 (primary motor cortex), BA6 (supplementary motor area), BA10 (fronto-parietal cortex), BA13 (insular cortex), BA24/32 (cingulate cortex), BA25 (subgenual anterior cingulate cortex) and BA45/47 (frontal gyrus). Two further ROIs of the subcortical region—the substantia nigra pars compacta and pars reticulata—were added from the California Institute of Technology reinforcement learning atlas, version 1.1 (CIT168)^69^.

To enable cortical branching, the angular threshold was set to a range of 60°–90°. Sampling was thresholded at a minimum length of 5 mm to avoid the inclusion of short streamlines and to prioritize long-range cortico-subthalamic projections. Streamline tracking between STN and substantia nigra aspects was performed based on a minimal tracking length of 10 mm, considering the distance between these two regions. To account for the exploratory nature of these connections, 20 iterations of topology-informed pruning were further implemented to limit the possibility of including false-positive streamlines^87^. Because of the role of these cortical regions in neuromodulation for affective disorders^88^, streamline tracking between BA24/25/32 was seeded from the limbic aspect of the STN (based on its definition within the DISTAL atlas, version 1.1 (ref. ^28^)). In this case, a twofold dilation of the limbic STN was implemented to allow for limbic regions adjacent to the anterior STN to be included^6^, and streamlines were mirrored between hemispheres (thus increasing the streamline count per sampling to a total of 3,000) to limit the occurrence of potential spurious lateralization effects.

In addition, the creation of this anatomically inclusive pathway atlas was complemented by representations of the anterior thalamic radiation, the cerebellothalamic tract, the dentato-rubro-thalamic tract and the fasciculus subthalamicus from the DBS Tractography Atlas, version 1 (ref. ^27^). Finally, definitions of the ansa and fasciculus lenticularis as well as subthalamic-pallidal connections between STN and all pallidal nuclei were added from the Basal Ganglia Pathway Atlas^26^, comprising interconnections between STN and internal pallidum (GPi), STN and GPe, Gpi and STN (associative and somatomotor aspects) and Gpe and STN (associative and somatomotor aspects).

Last, we aimed to demonstrate the validity of our findings in the face of disease-specific connectivity alterations in consequence of two exemplary brain circuit disorders. To this end, we repeated our DBS Fiber Filtering approach in a group connectome informed on diffusion scans acquired before surgery in the *n* = 6 patients with OCD (one female, mean age = 45.50 ± 10.52 years) from London. The supplementary materials of the original publication^73^ contain more detailed information on scanning parameters. In brief, transformations from patient space into ICBM 2009b NLIN asymmetric space were derived using ANTs (http://stnava.github.io/ANTs/)^75^. dMRI data were pre-processed using FMRIB Software Library (FSL)^89^, release 6.0 (https://fsl.fmrib.ox.ac.uk/fsl/fslwiki/FSL) (topup and eddy). A generalized *q*-sampling approach^90^ was performed as implemented in DSI-Studio (http://dsi-studio.labsolver.org/). In total, 500,000 streamlines were deterministically tracked per patient with an angular threshold of 30 and a smoothing factor of 1, and default settings were kept for all remaining parameters.

A second disease-matched group connectome^28^ was finally implemented that had previously been calculated based on data by *n* = 85 patients with PD (28 females; mean age = 59.48 ± 10.39 years) from the Parkinson’s Progressive Marker Initiative^91^ (PPMI; https://www.ppmi-info.org/). This connectome (PPMI-85, version 1.1; openly available from https://www.lead-dbs.org/helpsupport/knowledge-base/atlasesresources/normative-connectomes/) has been repeatedly used in the DBS context (for example, in refs. ^19,45^). Details on scanning parameters can be derived from the project website (https://www.ppmi-info.org/), and specifics on the creation of the resulting PD group connectome are reported elsewhere^28^. In brief, a fast diffeomorphic image registration algorithm^92^ (Diffeomorphic Anatomical Registration Through Exponentiated Lie Algebra (DARTEL)), as implemented in SPM12 (http://www.fil.ion.ucl.ac.uk/spm/software/spm12/) and Lead-DBS, version 2.0 (ref. ^60^), was applied per subject, to estimate a nonlinear deformation field into ICBM 2009b NLIN asymmetric space based on T2-weighted acquisitions. To determine global streamline sets, a generalized *q*-sampling approach^90^ was run as available in DSI-Studio (http://dsi-studio.labsolver.org/). Specifically, 20,000 streamlines were sampled for each patient, using seeds within a white matter mask derived from segmenting the T2-weighted acquisitions using SPM12. Subsequently, the streamline set per patient was standardized into MNI space, employing the methodology described in refs. ^93,94^.

Of note, the terms ‘fibers’ or ‘tracts’ should ideally be reserved for anatomical images and not be used to refer to derivatives of tracking algorithms delineating pathways based on water molecule diffusion within the brain. Instead, dMRI-based tractography is an indirect estimate of physical connections—or axons—and cannot inform on their directionality within the brain^5^. Thus, we speak of ‘streamlines’ to refer to tracking results throughout the manuscript. For reasons of consistency with previous publications, we solely maintain the term ‘DBS Fiber Filtering’ here to denote our streamline modeling approach.

### Retrospective and prospective streamline model validations

#### Retrospective model validations

Models informed on data points from the discovery cohort were further externally validated based on fully independent data (see above for the exception of the patients with OCD from London). In the two retrospective validation analyses, this strategy was carried out using the exact same approach as the CV analyses performed within the discovery sample—that is, by calculating the peak magnitude of each E-field at the intersection with each streamline of the respectively corresponding model (PD/OCD) and correlating the resulting aggregated value (weighted peak 5% of Fiber R score) with empirical clinical improvements.

#### Prospective model validations

In both patient cases receiving model-guided stimulation parameter optimization, reprogramming for clinical purposes took place based on a multi-disciplinary assessment of DBS location and streamline information. In the first reprogramming case of a patient with PD from Würzburg implanted to the STN, UPDRS-III scores were taken under DBS and dopaminergic medication OFF (after a 12-h-long washout period), under active DBS (medication OFF) with clinical DBS settings as well as under settings that maximized overlap of stimulation volumes with the PD streamline model. All conditions were assessed 3 months after surgery, and both clinical and streamline-informed DBS settings had been active for at least 24 h at the time of testing. The patient with PD was blinded to the DBS settings (clinically optimized versus streamline-informed) and evaluated by an independent physician blinded to the programming conditions. In the second reprogramming case of a patient with OCD from Boston receiving DBS to the VC/VS region, Y-BOCS scores were taken 1 month after surgery under clinical parameters as well as under parameters based on consideration of the OCD streamline model, and both conditions were compared to pre-surgical baseline. Of note, the patient was reprogrammed for clinical purposes, after clinically optimized stimulation parameters had failed to provide sufficient symptom relief. Thus, neither the patient nor the team of treating physicians was blinded to the activation of DBS nor to the programming condition (clinically optimized versus streamline-informed) during the evaluation of stimulation effects.

Third, the patient with OCD from the São Paulo center was treated via bilateral lead implantation surgery to the STN as well as stimulation parameter programming, which were both fully informed on the OCD streamline model. Y-BOCS outcome 1 month after streamline-based surgery and programming was finally contrasted to the severity of pre-operative OCD symptomatology. Given that only one condition was tested (streamline-optimized DBS), neither the evaluating physician nor the patient was blinded to the activation of DBS during the assessment.

In all three prospective patient cases, a pre-operative diffusion scan had been acquired, so that patient-specific tractography could be performed to confirm agreement between individual streamlines and normative connectivity models.

### Dysfunction mappings at the level of indirect pathways

Besides hyperdirect fronto-cortical interconnections, the STN receives indirect projections from the striato-pallido-fugal system^29^. In second instance, we, thus, sought to understand whether and in which way indirect anatomical connections would be partitioned as a function of stimulation impact on disorder-wise core symptom domains. To this end, we appended an additional DBS Fiber Filtering analysis informed on pallido-subthalamic connections that had been extracted from the Basal Ganglia Pathway Atlas^26^ while keeping remaining model parameters consistent.

### Reporting summary

Further information on research design is available in the Nature Portfolio Reporting Summary linked to this article.

## Online content

Any methods, additional references, Nature Portfolio reporting summaries, source data, extended data, supplementary information, acknowledgements, peer review information; details of author contributions and competing interests; and statements of data and code availability are available at 10.1038/s41593-024-01570-1.

### Supplementary information

Supplementary information Supplementary Figs. 1–12 and Supplementary Tables 1–11.

Reporting Summary

## Data Availability

Detailed patient-wise demographic and clinical information is available in Supplementary Tables 2–5, 7, 8 and 11 in anonymized form. Patient imaging data cannot be publicly shared as this would compromise patient privacy according to current data protection regulations. They are, however, available from the principal investigators of the collecting sites upon reasonable request within the framework of a data-sharing agreement. Inquiries for further information and data-sharing requests should be directed to the corresponding authors of this manuscript (A.H., ahorn1@bwh.harvard.edu, or N.L., ningfei.li@gmail.com) who commit to replying to any request within a timeframe of 30 d. Anonymized E-field derivatives along with pre- and postoperative clinical outcome scores of patients analyzed for the purpose of the present manuscript can be retrieved via a dedicated public repository, which is embedded within the Open Science Framework (https://osf.io/zu9c6/)^95^. The atlas of sweet streamline profiles and sweet spots of all four disorders is also included in this repository. In addition, this atlas is openly available within Lead-DBS software, version 3.0 (https://www.lead-dbs.org/). Although a processed version of the HCP 985 Connectome^21^ can be requested from the corresponding authors, source data are freely accessible via the repository of the HCP (https://www.humanconnectome.org/study/hcp-young-adult/document/1200-subjects-data-release). Furthermore, the DBS Tractography Atlas, version 2.1, can be openly downloaded (https://github.com/netstim/DBS-Tractography-Atlas.git). The HCP-1,065 diffusion source data^85^ used to inform this atlas can be openly accessed via DSI-Studio (https://brain.labsolver.org/hcp_template.html; fiber orientation maps at 1-mm resolution). The following normative resources have been made openly available by the original authors: the MGH 760 µm Connectome^67^ (https://datadryad.org/stash/dataset/doi:10.5061/dryad.nzs7h44q2) and the Basal Ganglia Pathway Atlas^26^ (https://osf.io/mhd4z/). The PD-matched PPMI-85 connectome can be openly and publicly derived via the Lead-DBS knowledge base (https://www.lead-dbs.org/helpsupport/knowledge-base/atlasesresources/normative-connectomes/). Source data used for calculation of this connectome can be freely accessed via the homepage of the Parkinsonʼs Progression Markers Initiative (https://www.ppmi-info.org/access-data-specimens/download-data). The OCD matched connectome can be shared by the corresponding authors upon reasonable request (see contact details stated above). Source data of patients with OCD employed to calculate this connectome cannot be publicly shared due to patient privacy restrictions. The DISTAL atlas, version 1.1 (ref. ^28^), and the CIT168 atlas, version 1.1 (ref. ^69^), are openly available via the Lead-DBS knowledge base (https://www.lead-dbs.org/helpsupport/knowledge-base/atlasesresources/atlases-2/) and come pre-installed with the Lead-DBS software. The JHU atlas parcellation^68^ is openly accessible as a pre-installation within the Surf Ice software (https://www.nitrc.org/projects/surfice/).

## Code availability

The entirety of code used in the analyses presented in this work is openly available within the Lead-DBS environment (https://github.com/leaddbs/leaddbs).
