# Supplementary material for: Mapping dysfunctional circuits in the frontal cortex using deep brain stimulation
Source: Nat Neurosci. 2024 Feb 22;27(3):573–86. doi: 10.1038/s41593-024-01570-1 (PMC10917675; doi:10.1038/s41593-024-01570-1)
Supplement: Supplementary file 1 — Supplementary Figs. 1–12 and Supplementary Tables 1–11. [file 41593_2024_1570_MOESM1_ESM.pdf]

nature neuroscience

Article

https://doi.org/10.1038/s41593-024-01570-1

Mapping dysfunctional circuits in the frontal cortex using deep brain stimulation

In the format provided by the authors and unedited

Table of Contents

Supplementary Figures pp. 3-14

Fig. S1: Influence of electrical field threshold on sweet spot mapping results. **pp. 3-4**

Fig. S2: Anatomical relationship of dysfunction mappings at the streamline level across disorders. **p. 4**

Fig. S3: Unthresholded sweet and sour streamline landscapes. **p. 5**

Fig. S4: Visualization of spatial uncertainty in dysfunction mappings at the streamline level. **p. 6**

Fig. S5: Sweet spots and connected streamlines based on a Gaussian fit to standard electrode contacts. **p. 7**

Fig. S6: Visualization of specificity in sweet streamline segregations. **p. 8**

Fig. S7: Model specificity of disease-wise sweet streamlines in explaining clinical outcome variance. **p. 9**

Fig. S8: Influence of choice of connectome on the topography of dysfunction mappings at the streamline level. **p. 10**

Fig. S9: Topographical organization of dysfunction mappings informed on disease-matched connectomes. **p. 11**

Fig. S10: Comparable results based on restricted vs. unrestricted dystonia samples. **p. 12**

Fig. S11: Optimization of normalization warp fields at the subthalamic level. **p. 13**

Fig. S12: Comparison of the topographical organization of cortico-subthalamic interconnections across individuals. **p. 14**

Supplementary Tables pp. 15-37

Table S1: Summary of demographic and clinical patient characteristics within each discovery cohort. **pp. 15-17**

Table S2: Patient-wise demographic and clinical characteristics of the dystonia discovery cohorts. **pp. 18-20**

Table S3: Patient-wise demographic and clinical characteristics of the Tourette's syndrome discovery cohorts. **pp. 21**

Table S4: Patient-wise demographic and clinical characteristics of the Parkinson's disease discovery cohorts. **pp. 22-25**

Table S5: Patient-wise demographic and clinical characteristics of the obsessive-compulsive disorder discovery cohorts. **pp. 26-27**

Table S6. Summary of demographic and clinical patient characteristics within each retrospective model validation cohort. **pp. 28-29**

Table S7: Patient-wise demographic and clinical characteristics of the Parkinson's disease validation cohort. **pp. 30-31**

Table S8: Patient-wise demographic and clinical characteristics of the obsessive-compulsive disorder validation cohorts. **pp. 32-34**

Table S9: Peak voxel coordinates of subthalamic sweet spots and of disease-specific cortical sites of interconnection with sweet tracts. **p. 35**

Table S10: Overlap between connected streamlines shared among disorders and disease-specific sweet streamlines. **pp. 35**
Table S11: Patient-wise demographic and clinical characteristics of patient cases for prospective model validations. **pp. 36-37**
References **pp. 38-39**

Supplementary Figures

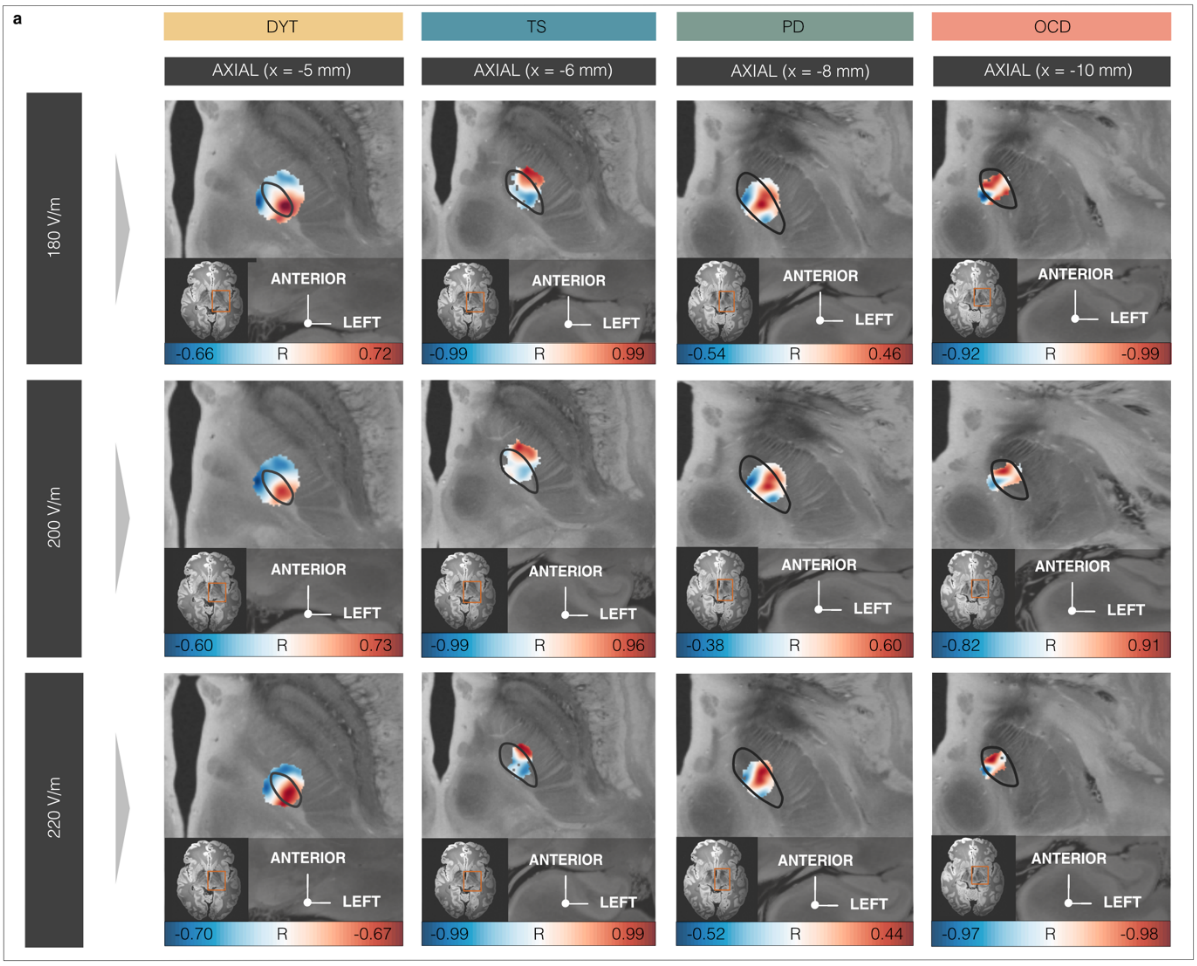

b

E-FIELD THRESHOLD	IN-SAMPLE	5-FOLD CV	IN-SAMPLE	5-FOLD CV	IN-SAMPLE	5-FOLD CV	IN-SAMPLE	5-FOLD CV
180 V/m	R = 0.57; p = 2.000e-4	R = 0.42; p = 0.002	R = 0.48; p = 2.000e-4	R = 0.38; p = 5.999e-4	R = 0.81 p = 7.998e-4	R = -0.49; p = 0.067	R = 0.52; p = 0.027	R = 0.36 p = 0.129
200 V/m	R = 0.56; p = 2.000e-4	R = 0.41; p = 0.001	R = 0.49; p = 2.000e-4	R = 0.41; p = 2.000e-4	R = 0.70; p = 0.009	R = -0.51; p = 0.055	R = 0.44; p = 0.055	R = 0.53; p = 0.023
220 V/m	R = 0.56; p = 2.000e-4	R = 0.40; p = 0.001	R = 0.49; p = 2.000e-4	R = 0.44; p = 2.000e-4	R = 0.71; p = 0.005	R = -0.50; p = 0.057	R = 0.47; p = 0.046	R = 0.52; p = 0.025

Fig. S1: Influence of electrical field threshold on sweet spot mapping results. (a) Sweet and sour spot configurations in dystonia (DYT; n = 56), Parkinson's disease (PD; n = 94), Tourette's syndrome (TS; n = 14) and obsessive-compulsive disorder (OCD; n = 19) show consistency across different electrical field (E-field) thresholds when remaining model parameters are kept consistent. Relative anatomical position of disease-wise maps based on E-field thresholds of 180 V/m, 200 V/m, and 220 V/m are shown in axial view of a comparable slice per disorder. Sweet and sour spots are presented with regard to the left subthalamic nucleus (STN; black outlines) in template space, derived from the DBS Intrinsic Template (DISTAL) atlas [1], and in superposition to an 100 µm ex-vivo template [2]. Color-coding of voxels represents Spearman's rank correlation strength (warm colors for positive, cool colors for negative associations) between E-field magnitudes and clinical improvements. **(b)** In-sample correlations and five-fold cross-validation (CV) results are comparable with main results of the present manuscript. Spearman correlations test for association between the similarity in E-field peaks with disease-wise models of sweet spots (expressed as mean Sweet Spot score under each E-field, averaged for bilateral scores per patient) across the cohort (two-sided tests). Grey

shaded areas represent 95% confidence intervals. *Abbreviations:* CV, cross-validations; TS, Tourette's syndrome; OCD, obsessive-compulsive disorder; PD, Parkinson's disease.

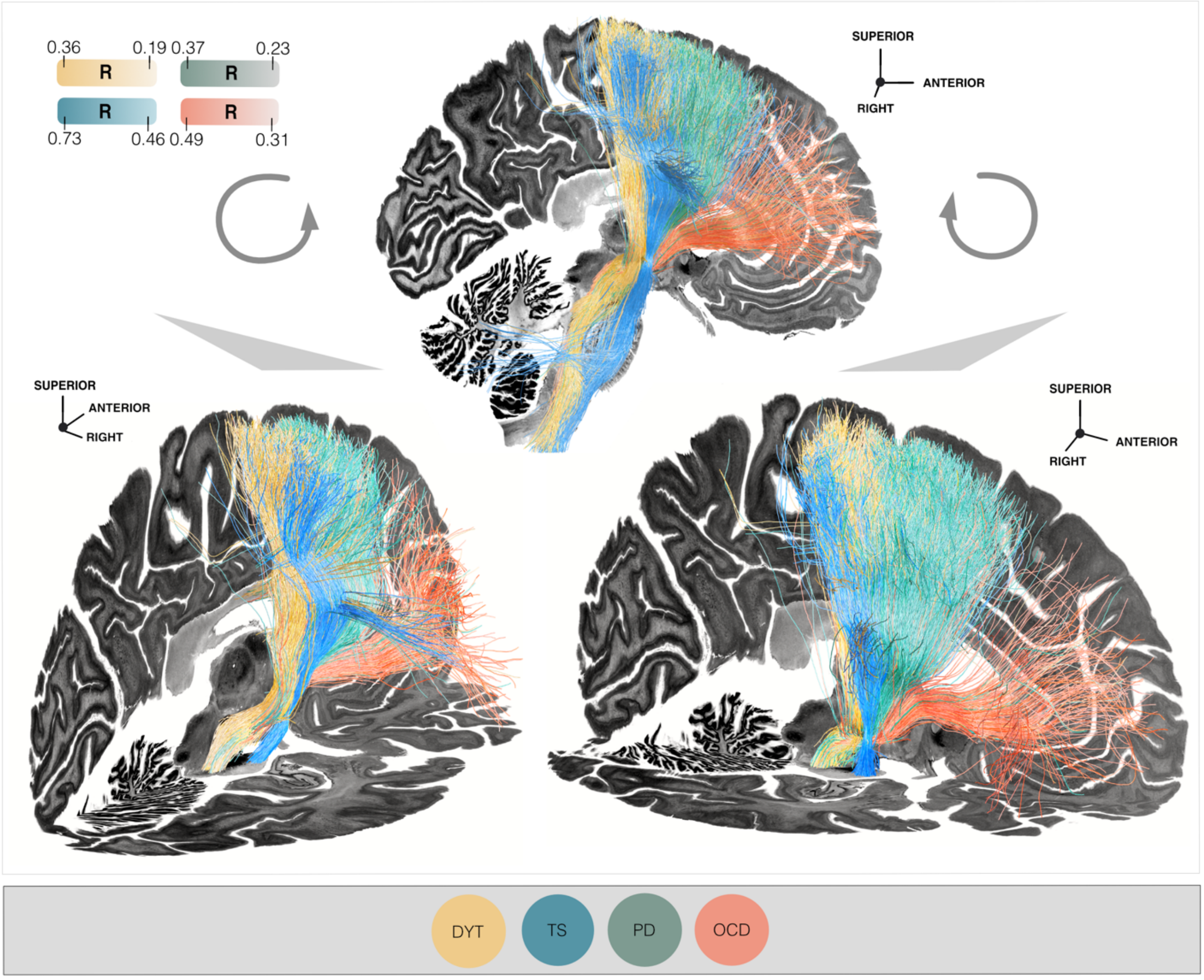

Fig. S2: Anatomical relationship of dysfunction mappings at the streamline level across disorders. Posteriorly **(a)** and anteriorly **(b)** tilted views of dysfunction mappings resulting from deep brain stimulation (DBS) Fiber Filtering in dystonia (DYT; n = 56), Parkinson's disease (PD; n = 94), Tourette's syndrome (TS; n = 14) and obsessive-compulsive disorder (OCD; n = 19). Disease-specific analyses were informed on streamlines extracted from a population-based group connectome [3]. Streamlines connected to stimulation volumes within each respective disorder were weighted by their association with clinical improvement in the disorder-specific clinical outcome measure, using Spearman's rank correlation. Each streamline is colored by R-value magnitude, with darker colors representative of higher correlations. Results are displayed in relation to a sagittal (x = 5 mm) and an axial slice (z = -17 mm) of the Big Brain template [4] in ICBM 2009b Non-linear Asymmetric ('MNI') space for anatomical reference.

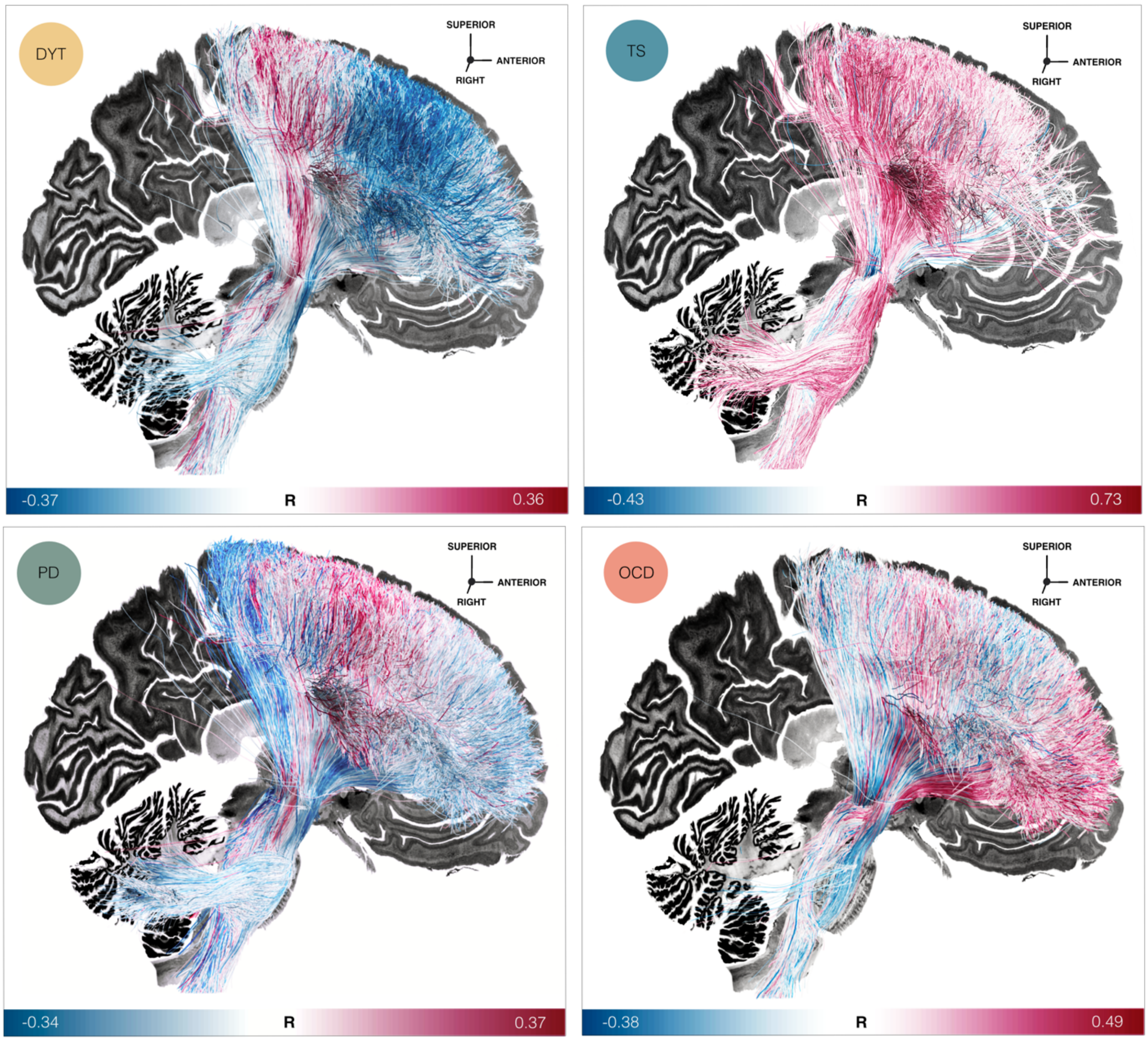

Fig. S3: Unthresholded sweet and sour streamline landscapes. Unthresholded models of optimal electrode connectivity for maximized stimulation-related improvement (sweet streamlines, in red) and worsening (sour streamlines, in blue) in disease-specific dysfunction resulting from Deep Brain Stimulation (DBS) Fiber Filtering in dystonia (DYT; n = 56), Parkinson's disease (PD; n = 94), Tourette's syndrome (TS; n = 14) and obsessive-compulsive disorder (OCD; n = 19). Each streamline comprised within disease-specific models is color-coded by an R-value which denotes the association between the strength of its modulation with clinical outcomes across patients in the respective disorder cohort (as calculated using Spearman's rank correlation). Accordingly, streamlines whose modulation is of highest importance for treatment success receive dark red colors, while those whose modulation is most relevant for suboptimal outcomes are colored in dark blue. The whiter the streamline, the less discriminative it was in coding for optimal vs. suboptimal outcomes. Disorder-wise results are overlayed on top of a sagittal slice (x = -5 mm) of the Big Brain template [4].

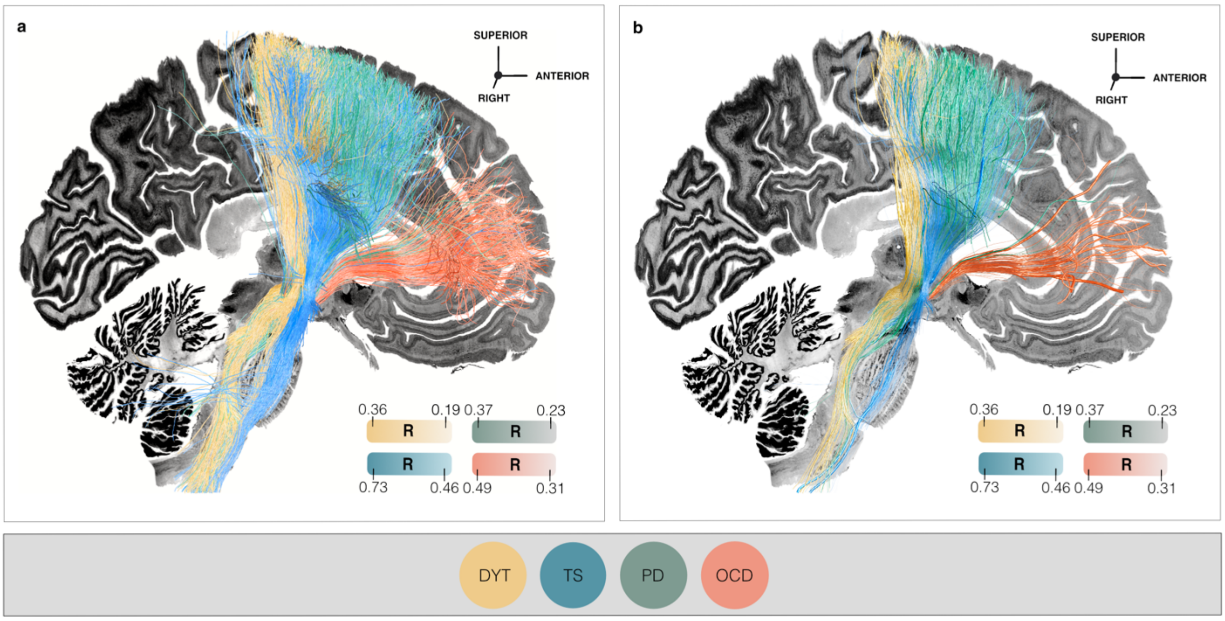

Fig. S4: Visualization of spatial uncertainty in dysfunction mappings at the streamline level. (a) Segregation into therapeutic streamline bundles achieved by means of deep brain stimulation (DBS) in dystonia (DYT; n = 56), Parkinson's disease (PD; n = 94), Tourette's syndrome (TS; n = 14) and obsessive-compulsive disorder (OCD; n = 19), with streamline thickness attributed in a universal fashion across the connectome (as done in the visualizations illustrating the main analysis of the present manuscript). **(b)** The same DBS Fiber Filtering results are represented, this time with streamline thickness informed based on the negative log(p)-value of the Spearman's rank correlation coefficient the respective streamline had received in the corresponding disorder-wise model. Accordingly, lower p-values are representative of thicker streamlines. Applying this procedure demonstrates a higher degree of uncertainty of TS results (given the low n) but reinforces the OCD results which were also confirmed in an unseen validation cohort (see **Fig. 6** in the main manuscript). Association strength between the degree of streamline modulation and clinical improvements across the disease cohort is used to color-code streamlines in each disorder-specific color, with darker colors indicating higher correlations. Results are represented against the backdrop of a sagittal slice (x = -5 mm) of the Big Brain template [4].

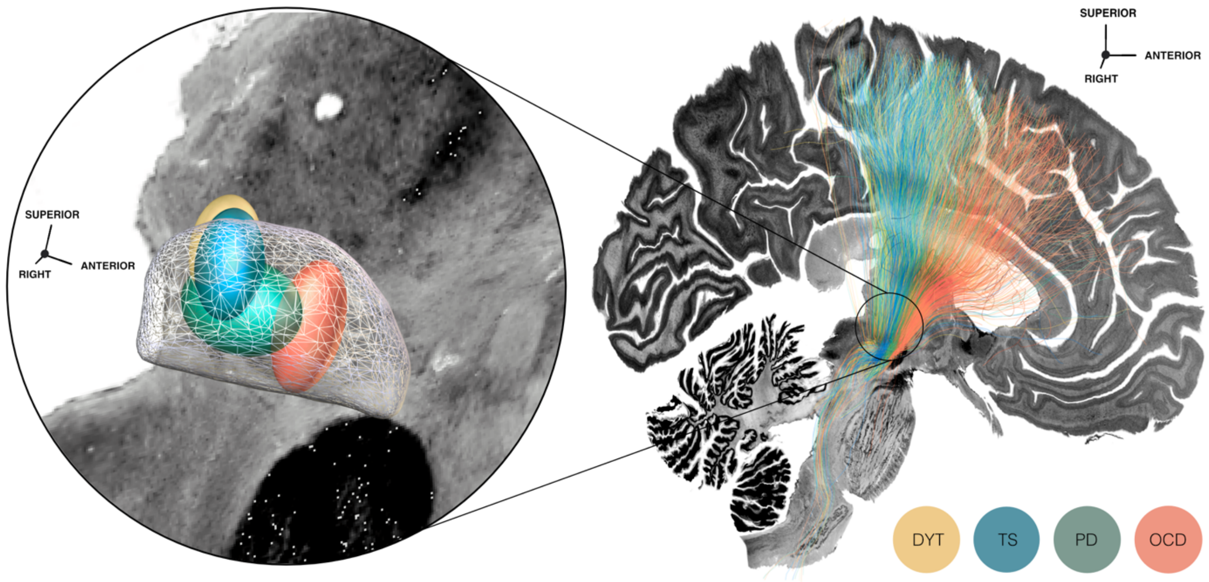

Fig. S5: Sweet spots and connected streamlines based on a Gaussian fit to standard electrode contacts. *Left:* Sweet spot maps are fit to the standard electrode contact (second-to-lowest) in form of a Gaussian for dystonia (DYT; n = 56), Parkinson's disease (PD; n = 94), Tourette's syndrome (TS; n = 14) and obsessive-compulsive disorder (OCD; n = 19) cohorts. Results are represented relative to a three-dimensional model of the left subthalamic nucleus (STN) in template space derived from the DBS Intrinsic Template (DISTAL) atlas [1]. *Right:* Streamlines are seeded from the Gaussian sweet spot maps per disorder and displayed against a sagittal slice (x = -5 mm) of the Big Brain template [4]. Note that the partitioning of disease-wise sweet streamline bundles expectedly vanishes entirely for those disorders known to be treated by implantation to the same posterolateral site of the STN, encompassing DYT, TS, and PD. Segregation is retained for OCD streamlines, which is expected given the different (anteromedial) subthalamic implantation site typically chosen by surgeons to treat this disorder. Nonetheless, the resulting streamlines course much more dorsally compared to the sweet streamline mapping retrieved using Deep Brain Stimulation (DBS) Fiber Filtering [5]. These findings support the notion that segregations in DBS Fiber Filtering-based dysfunction mappings are not merely driven by a-priori differences in electrode placement between disease-wise cohorts. Instead, they are majorly influenced by the importance of modulating these streamlines for clinical outcomes.

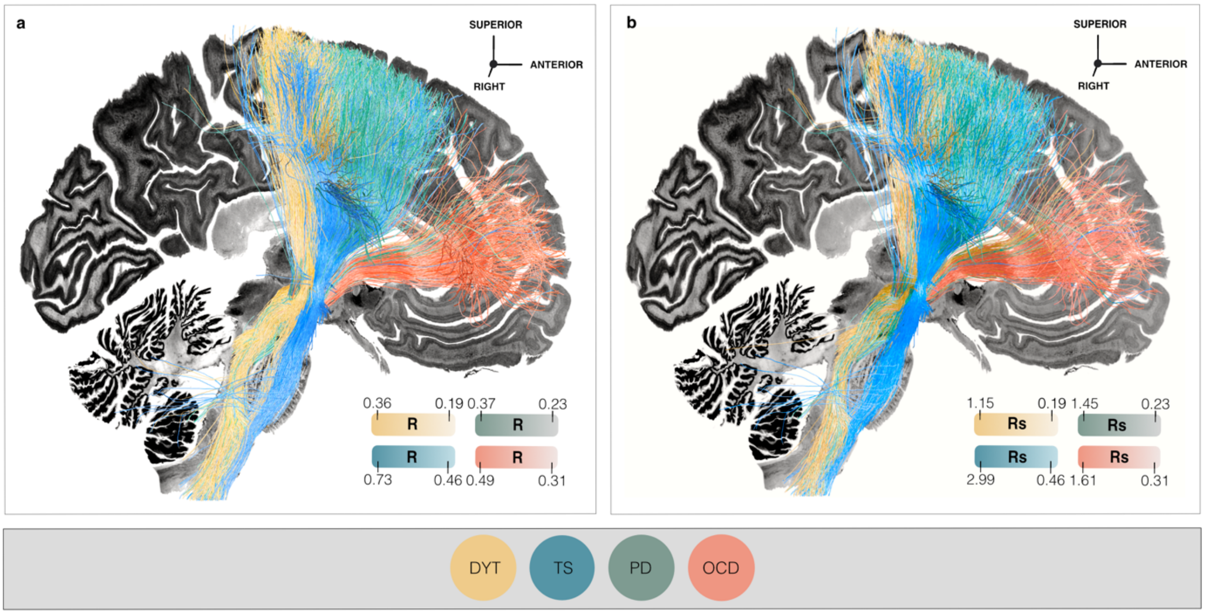

Fig. S6: Visualization of specificity in sweet streamline segregations. (a) Mappings of improvements in disease-wise dysfunction following deep brain stimulation (DBS) at the level of streamlines in dystonia (DYT; n = 56), Parkinson's disease (PD: n = 94), Tourette's syndrome (TS; n = 14) and obsessive-compulsive disorder (OCD; n = 19). Coloring of streamlines is graded by the magnitude of its R-value per disease, with streamlines whose modulation was associated with higher treatment benefit receiving more intense color values. The R-value of each streamline represents the result of a Spearman's rank correlation between streamline modulation strength and clinical improvement across the cohort. **(b)** In each disorder, coloring of the same therapeutic streamline model is graded by a dedicated specificity value (denoted as 'Rs'). For each streamline, this specificity value was calculated by dividing its R-value by the average of R-values that streamline had received within data of the remaining three diseases. In direct comparison, the resulting segregations look highly similar, which corroborates the specificity of segregated dysfunction attributions resulting from DBS Fiber Filtering [5]. Both views are shown in relation to an axial slice (x = -5 mm) of the Big Brain template [4] in ICBM 2009n Non-linear Asymmetric ('MNI') space.

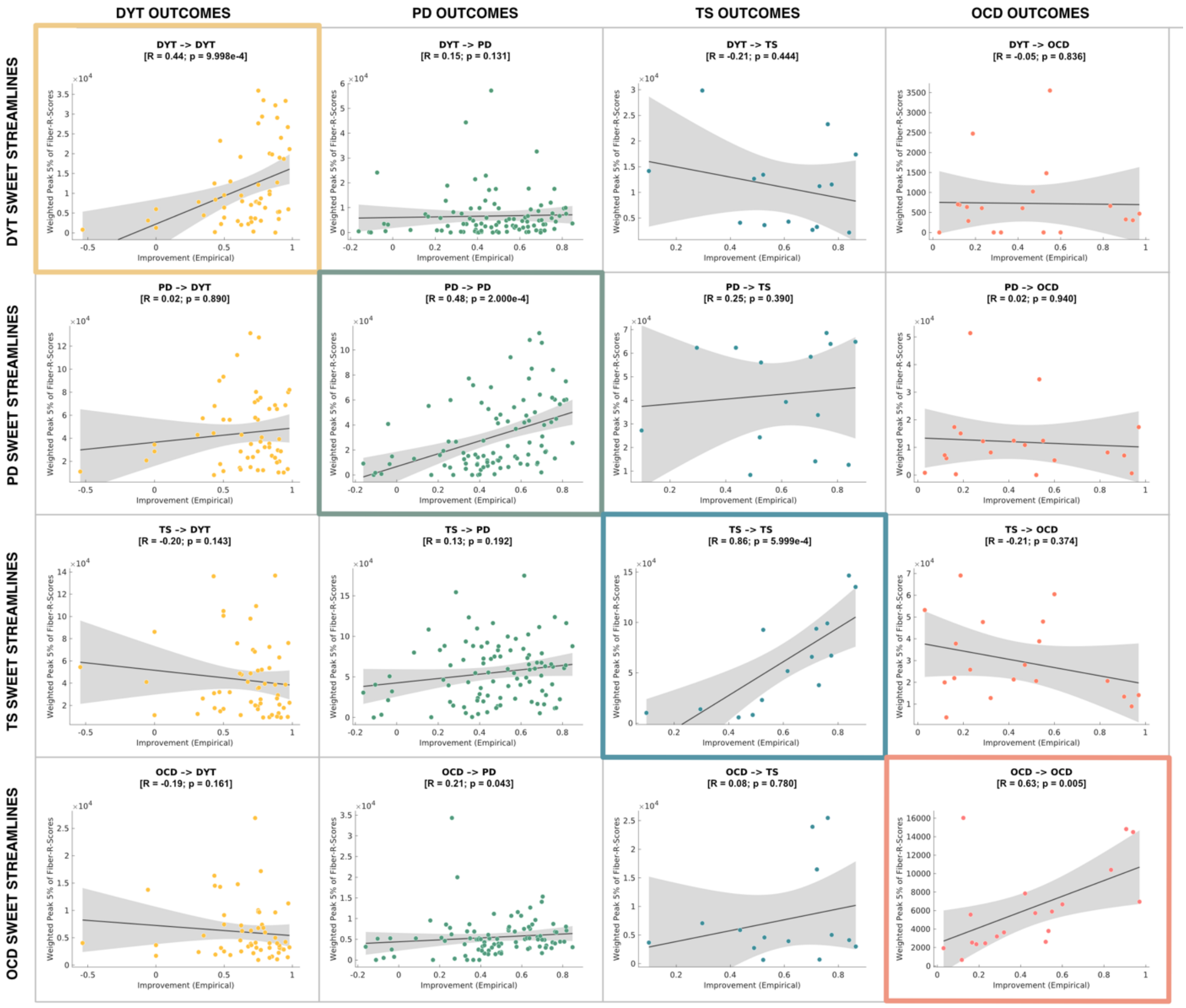

Fig. S7: Model specificity of disease-wise sweet streamlines in explaining clinical outcome variance. Based on the degree of overlap of electrical fields (E-fields) with sweet streamline profiles of all remaining three disorders, clinical improvement in each disease cohort is cross-predicted. More precisely, we tested for association between the stimulation magnitude of positive streamlines in a given disorder (i.e., the mean 5% of Fiber R-Scores under each E-field, averaged for bilateral scores per patient) and empirical clinical outcome in all remaining disorders using Spearman correlation (two-sided tests). P-values of these Spearman's correlations are based on permutation tests of 5,000 randomizations. This is done to confirm model specificity in explaining outcome variance within the respective domain of dysfunction. Streamline models considered here had been informed on a connectome calculated from 985 healthy participants from the Human Connectome Project [6]. Grey shaded areas represent 95% confidence intervals. *Abbreviations:* DYT, dystonia; TS, Tourette's syndrome; OCD, obsessive-compulsive disorder; PD, Parkinson's disease.

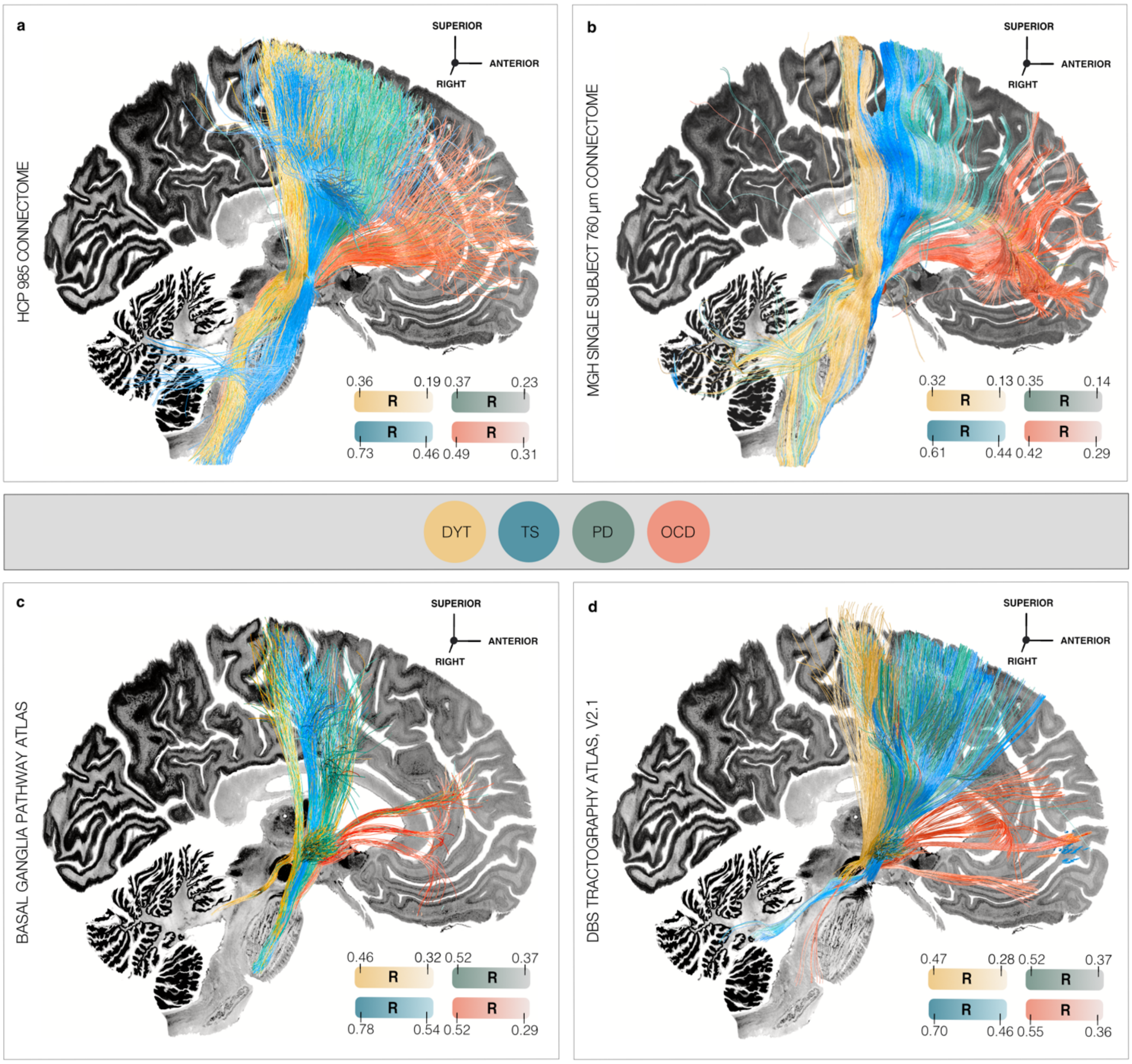

Fig. S8: Influence of choice of connectome on the topography of dysfunction mappings at the streamline level. To scrutinize the impact of a specific connectomic resource on the topographical organization of dysfunction attributions, we recalculated disease-wise deep brain stimulation (DBS) Fiber Filtering analyses using four different connectomes. A similar caudo-rostral topography emerged for clinically beneficial sets of streamlines in dystonia (DYT; n = 56), Parkinson's disease (PD; n = 94), Tourette's syndrome (TS; n = 14) and obsessive-compulsive disorder (OCD; n = 19), when they were filtered from the Human Connectome Project (HCP) 985 Connectome [3,6] **(a)**, the Massachusetts General Hospital (MGH) Single Subject 760 μm Connectome [7] **(b)**, the Basal Ganglia Pathway Atlas [8] **(c)**, as well as the DBS Tractography Atlas, v2.1 **(d)**. Across panels, thresholded streamline models are displayed in disease-specific color. Within each disease-specific model, each streamline received a value coding for the strength of association between stimulation magnitude and clinical outcome across the disease cohort using Spearman's rank correlation, where more intense colors indicate higher correlation values. Results are shown against a sagittal slice (x = -5 mm) of the Big Brain template [4].

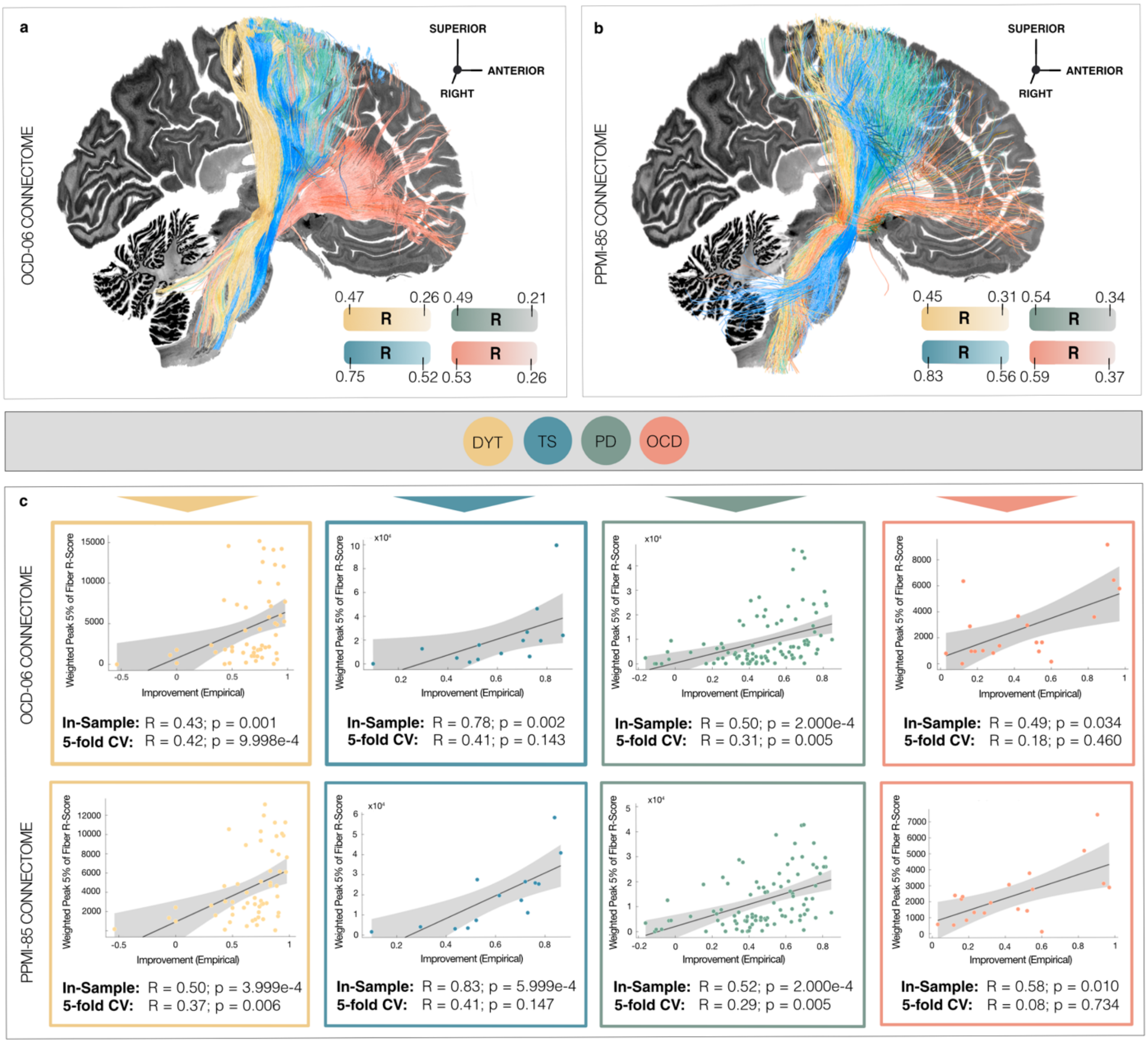

Fig. S9: Topographical organization of dysfunction mappings informed on disease-matched connectomes. To investigate the influence of disease-specific connectivity alterations on dysfunction mappings reported in the main manuscript, the deep brain stimulation (DBS) Fiber Filtering analysis for dystonia (DYT; n = 56), Parkinson's disease (PD; n = 94), Tourette's syndrome (TS; n = 14) and obsessive-compulsive disorder (OCD; n = 19) cohorts was repeated based on two disease-matched connectomes. This comprised **(a)** an OCD matched connectome calculated on diffusion-weighted imaging based tractography of n = 6 OCD patients. The same analysis was repeated, this time informed on **(b)** a previously derived PD matched connectome [1] based on data by n = 85 PD patients from the Parkinson's Disease Progressive Marker Initiative [9] (PPMI; https://www.ppmi-info.org/). In both panels, streamline models of all four disorders are represented in conjunction with each other, in thresholded form and highlighted in disease-specific color. Within each disease model, streamlines were color-coded by the degree of Spearman's rank correlation between stimulation impact (peak electrical field magnitude) and clinical improvements across the respective disease cohort, with higher color intensities indicating higher correlations. Results are overlayed on top of a sagittal slice (x = -5 mm) of the Big Brain template [4]. **(c)** Finally, in-sample correlations and five-fold cross-validations (CV) were carried out to scrutinize the capability of these streamline models to explain outcome variance within the respective clinical outcome scale. For this purpose, Spearman correlations were performed between the stimulation magnitude of positive streamlines (mean 5% of Fiber R-scores attributed to each electric field, averaged for bilateral scores per patient) and empirical clinical improvements (two-sided tests). Grey shaded areas are indicative of 95% confidence intervals.

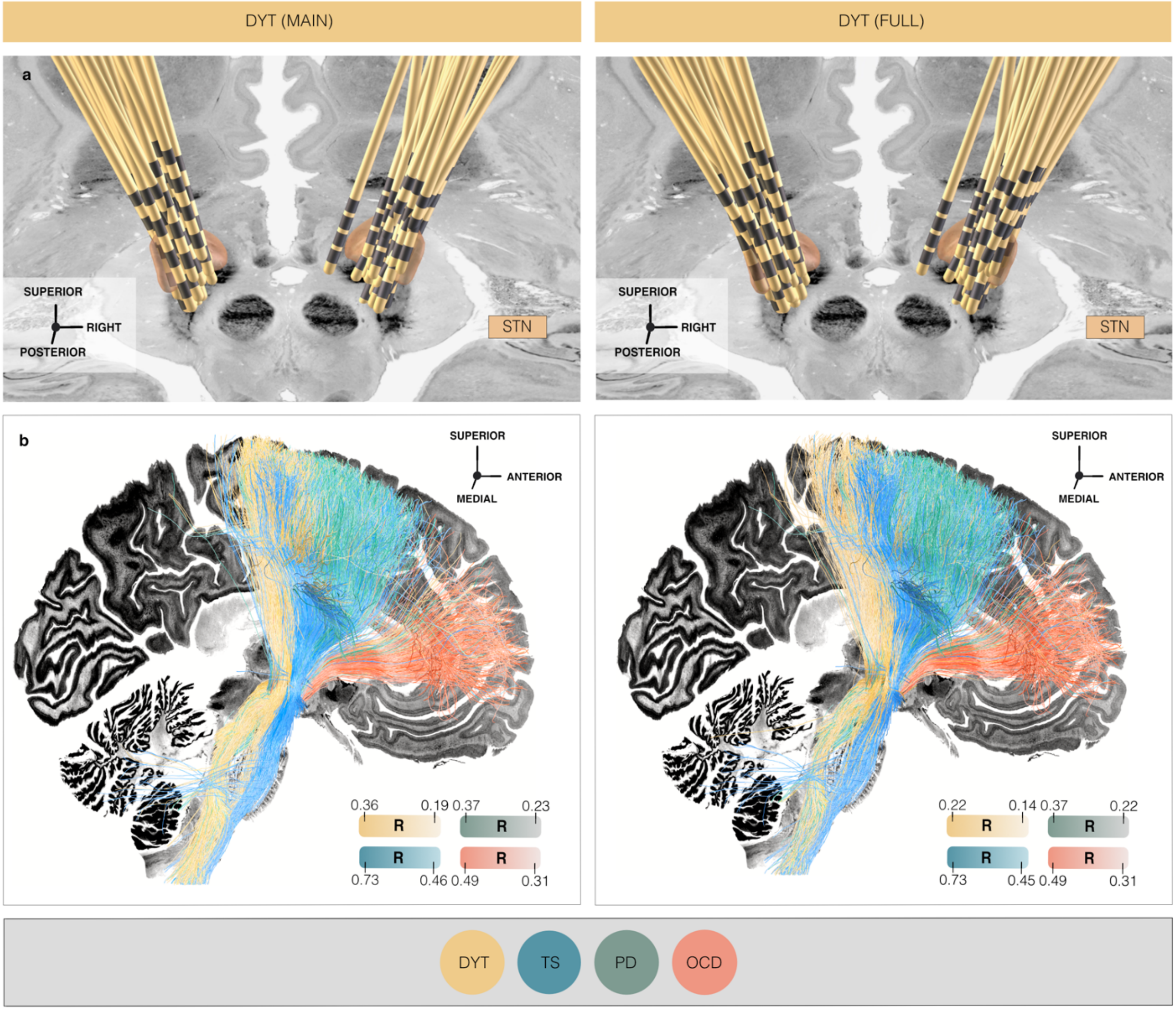

Fig. S10: Comparable results based on restricted vs. unrestricted dystonia samples. Respectively left panels show anatomical electrode placement relative to the subthalamic nucleus (STN) **(a)**, as well as disease-wise deep brain stimulation (DBS) Fiber Filtering results **(b)** in the restricted (main) dystonia (DYT) sample. The data of DYT patients reported within the main body of the manuscript followed a more conservative scheme of exclusion criteria. In this case, only patients with baseline scores in the Burke-Fahn-Marsden Dystonia Rating Scale (BFMDRS) ≥ 5 and time points of follow-up assessment of ≥ 6 months were considered (n = 56) to ensure comparable and sufficiently stabilized stimulation effects across cohorts. The respectively right panels show electrode placement and dysfunction mapping results comprising the unrestricted (full) DYT sample (n = 70), which included the n = 14 additional patients from Shanghai. Streamlines in both panels are represented in a disease-wise color scheme, with the color intensity representative of the relationship of each streamline's modulation (peak electric field magnitude) with clinical outcome, as expressed via Spearman's rank correlations. More intense colors represent higher correlation strength. STN defined by the DBS Intrinsic Template (DISTAL) atlas [1], with an axial plane of the BigBrain template in 100 µm resolution [4] displayed as a backdrop. *Abbreviations:* TS, Tourette's syndrome; OCD, obsessive-compulsive disorder; PD, Parkinson's disease.

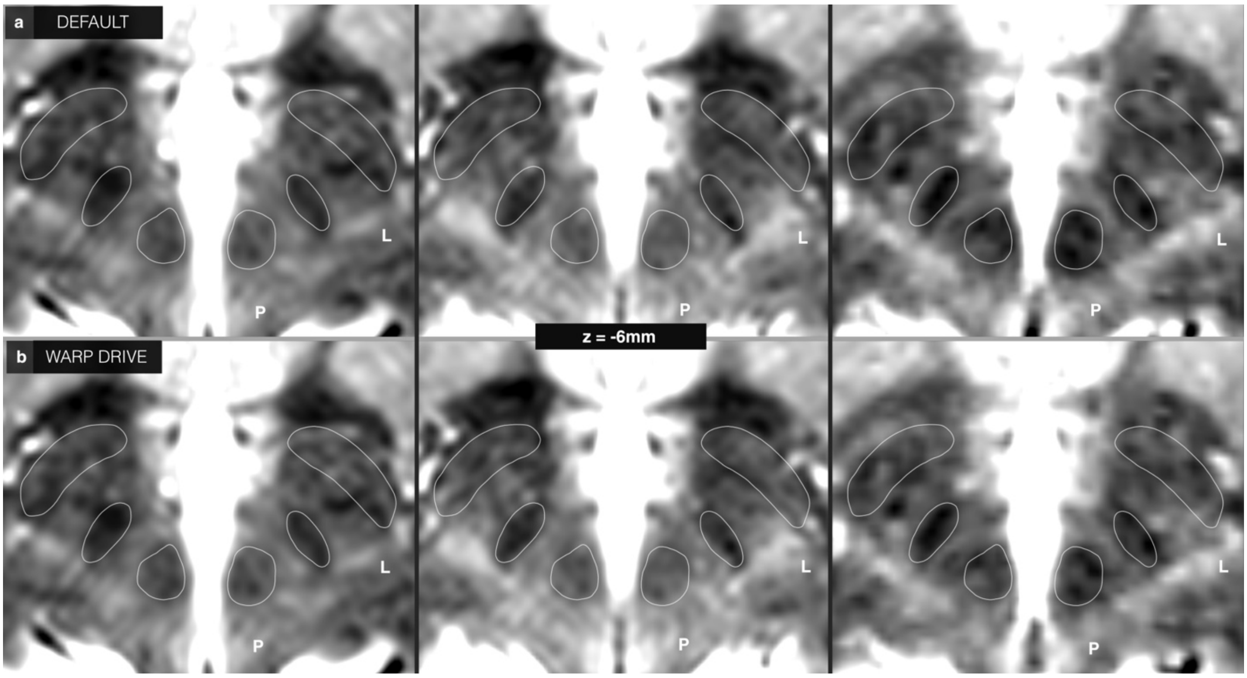

Fig. S11: Optimization of normalization warp fields at the subthalamic level. Three example cases of registrations between atlas template and individual brain anatomy are shown. Panel **(a)** demonstrates results following automatized normalization. Panel **(b)** shows results after the additional manual refinement of small mismatches with focus on the subthalamic nucleus (STN) region using the WarpDrive toolbox [10], as implemented in Lead-DBS, v3 software [11]. Outlines of displayed atlas structures are defined based on the DBS Intrinsic Template (DISTAL) atlas [1]. In most cases, no substantial changes needed to be carried out (such as in the leftmost case, in which automated results were excellent). In others, small refinements to better fit the patient STN to the atlas STN were carried out using WarpDrive.

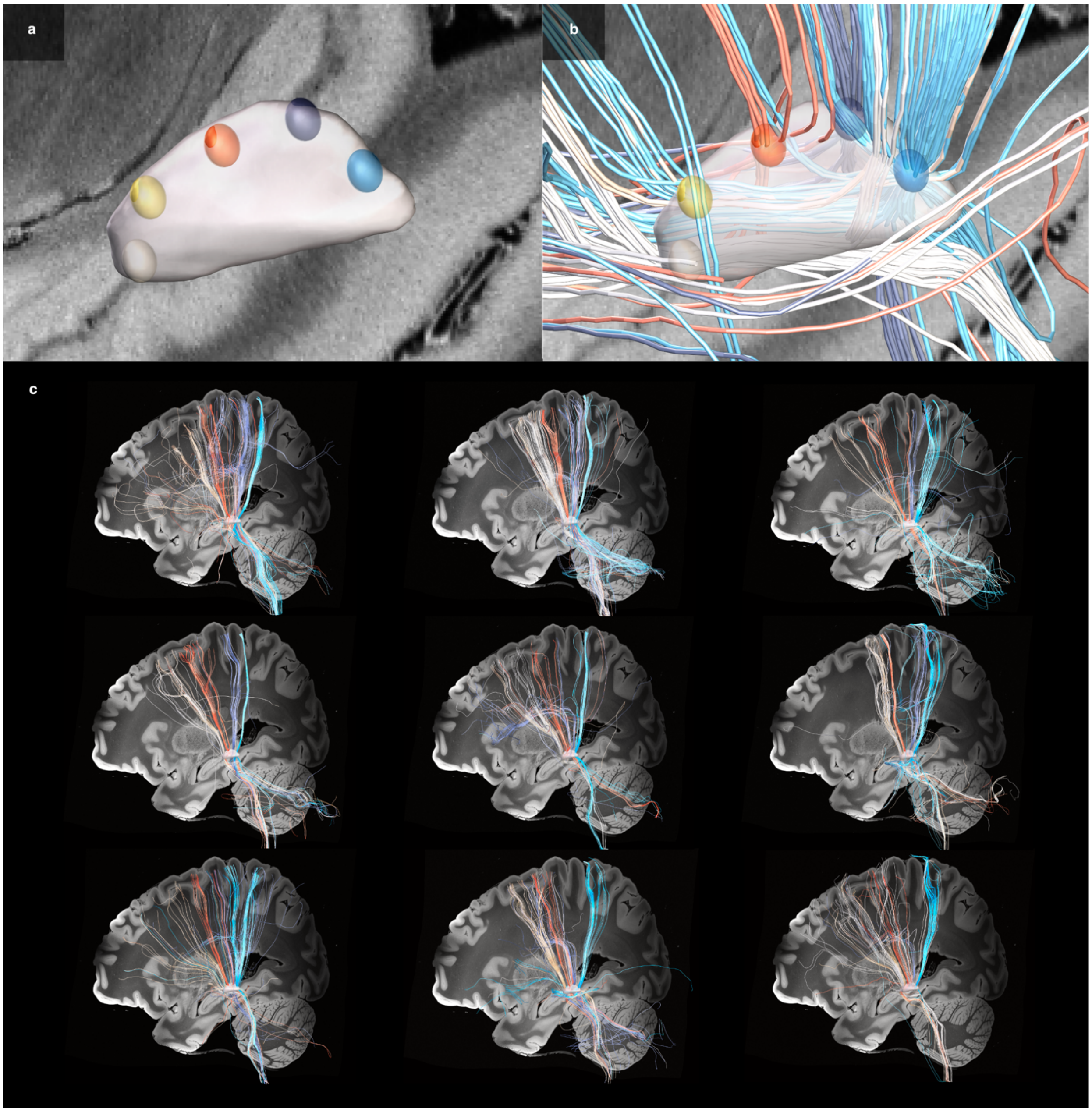

Fig. S12: Comparison of the topographical organization of cortico-subthalamic interconnections across individuals. (a) To interrogate the degree of inter-individual variability in cortico-subthalamic anatomical connectivity, five seed points were placed along the dorsal convex shape of the subthalamic nucleus (STN) in standard space, as represented by a three-dimensional model of the left STN from the DBS Intrinsic Template (DISTAL) atlas [1]. **(b)** Streamlines were tracked from these seed points in nine randomly selected healthy participants of the Human Connectome Project (HCP) [6], respectively. To allow for comparison of their anatomical organization, streamlines are tagged with a color that is matched to their respective seed point in panel (a). **(c)** Despite similarities in the general topographical organization across individual brains, visible differences in the granularities of streamline representations emerged (as discernible in color-coded fashion). Streamline tracking results are displayed against a sagittal slice (x = -30 mm) of the 7T MRI ex-vivo 100 µm human brain template [2].

Supplementary Tables

Table S1: Summary of demographic and clinical patient characteristics within each discovery cohort.

**Disease cohort**	**DYT**		**PD**		**TS**		**OCD**	
				**Demographic Information**				
**Cohort**	San Francisco	Shanghai	Berlin	Würzburg	Pisa/Milan	Shanghai	London	Grenoble
**Surgical DBS center**	University of California San Francisco	Ruijin Hospital Shanghai	Charité - Universitäts-medizin Berlin	University Hospital Würzburg	Fondazione IRCCS Instituto Neurologico Carlo Besta Milan	Ruijin Hospital Shanghai	National Hospital for Neurology and Neurosurgery	University Hospital Grenoble
**n (females)**	12 (7)	Main*: 44 (23); Full: 58 (31)	51 (17)	43 (12)	4 (2)	10 (1)	6 (1)	13 (9)
**Age at time of surgery (mean ± SD; range; in years)**	56.08 ±16.06; 16-68	Main*: 39.43 ± 21.06, 7-74; Full: 41.90 ± 20.60, 7-74	59.98 ± 7.93.; 42-75	60.49 ± 8.23; 46-74	34.00 ± 8.83; 26-46	23.10 ± 9.39; 14-38	45.50 ± 10.52; 37-62	39.15 ± 8.23; 27-53
**Disease duration at time of surgery (mean ± SD; range; in years)**	9.50 ± 9.54; 2-32	Main*: 5.94 ± 7.01, 0.5-30; Full:5.15 ± 6.36, 0.25-30	10.38 ± 3.85; 5-21	12.53 ± 4.46; 5-23	24.25 ± 6.08; 19-33	14.8 ± 9.38; 3-30	24.17 ± 3.92; 20-30	18.92 ± 8.96; 5-39
				**Clinical Outcome**				
**Main clinical outcome assessment**	BFMDRS [12] (total score; range 0-120, with higher scores corresponding to higher symptom burden)		UPDRS-III [13] (total score; range 0-199, with higher scores corresponding to higher symptom burden)		YGTSS [14] (global severity score; range 0-100, with higher scores corresponding to higher symptom burden)		Y-BOCS [15] (total score; range 0-40, with higher scores corresponding to higher symptom burden)	
**Time of FU (in months)**	6	Main*: mean = 19.09 ± 15.60 SD, range 6-84; Full: mean = 18.00 ± 15.56 SD, range 2-84	12	12	6	6	3 or 6 (post-amSTN phase)[+]	12

**Comparison basis for calculation of improvement values (baseline to DBS stimulation ON conditions)**	Pre- to postoperative	Pre- to postoperative	Postoperative ON vs. OFF stimulation (ON dopaminergic medication)	Postoperative ON vs. OFF stimulation (ON dopaminergic medication)	Pre- to postoperative	Pre- to postoperative	Pre- to postoperative	Pre- to postoperative
**Score at baseline (mean ± SD)**	16.88 ± 9.30	Main*: 19.09 ± 17.68; Full: 16.40 ± 16.57	38.56 ± 12.92	49.84 ± 12.36	89.25 ± 5.25	69.70 ± 10.19	36.17 ± 1.83	33.54 ± 3.76
**Score at time of FU under stimulation ON condition (mean ± SD)**	6.88 ± 5.90	Main*: 6.02 ± 9.21; Full: 5.25 ± 8.29	20.12 ± 8.82	24.47 ± 10.62	34.25 ± 15.97	28.30 ± 16.47	19.83 ± 10.57	18.92 ±10.74
**Relative improvement (mean ± SD; in %)**	52.07 ± 41.71	Main*: 69.90 ± 23.46; Full: 65.17 ± 28.79	45.34 ± 23.03	49.46 ± 23.97	61.50 ± 17.81	59.14 ± 24.09	45.00 ± 29.33	43.59 ± 31.56
**Absolute improvement (mean ± SD)**	10.00 ± 10.83	Main*: 13.07 ± 14.72; Full: 11.15 ± 13.60	18.44 ± 11.31	25.37 ± 13.30	55.00 ± 17.00	41.40 ± 17.59	16.33 ± 10.67	14.62 ± 10.75
**Imaging and DBS Specifications**								
**Imaging modality (postoperatively)**	MRI (n = 12)	Main*: CT (n = 44); Full: CT (n = 58)	MRI (n = 45), CT (n = 6)	CT (n = 43)	CT (n = 4)	CT (n = 10)	MRI (n = 6)	MRI (n = 10), CT (n = 3)
**Electrode models**	MDT 3389 (n = 12)	Main*: MDT 3387 (n = 17), PINS L302 (n = 17), SR 1210 (n = 10); Full: MDT 3387 (n = 22), PINS L302 (n = 26), SR 1210 (n = 10)	MDT 3389 (n = 51)	MDT 3389 (n = 44)	MDT 3389 (n = 4)	MDT 3387 (n = 2), SR 1210 (n = 7), PINS L302 (n = 1)	MDT 3389 (n = 6)	MDT 3389 (n = 14)

**Related citation**	Ostrem et al., 2011 [16]; 2016 [17]	Lin et al., 2019 [18]; He, 2021 [19]	Horn et al., 2017 [20]; 2019 [21]	Horn et al., 2017 [20]; 2019 [21]	Vissani et al., 2019 [22]	Dai et al., 2022 [23]	Tyagi et al., 2019 [24]	Polosan et al. 2019 [25]

Notes: *The full sample of dystonia (DYT) patients from the Shanghai center (n = 58) is based on more liberal inclusion criteria compared to the sample reported on within the main manuscript (n = 44). More conservative exclusion criteria (baseline scores on the Burke-Fahn-Marsden Dystonia Rating Scale [BFMDRS] ≥ 5 and time points of follow-up for postoperative assessments ≥ 6 months) were chosen for main analyses to ensure sufficiently stabilized and comparable deep brain stimulation (DBS) effects across cohorts. Results based on the full sample are reported in the supplements (Fig. S10). [+]OCD patients from London were bilaterally implanted with DBS electrodes to two different target sites (four electrodes per patient) – the anteromedial subthalamic nucleus (amSTN) and the ventral capsule/ventral striatum (VC/VS). Patients were randomly assigned to either receive 'pure amSTN' for the first three months or from months four to six postoperatively (and 'pure VC/VS' for the remainder of the first six months). Model set-up (based on the discovery cohort) was informed on stimulation parameters and corresponding clinical scores taken after the amSTN phase, while model validation was performed based on VC/VS-stimulation related data points. AmSTN stimulation was applied via a Medtronic (MDT) model of type 3389, while MDT 3387 leads were implanted for stimulation of the VC/VS target zone (considered only for model validations, see Tables S6 and S8 below). *Abbreviations:* CT, computed tomography; DBS, deep brain stimulation; FU, follow-up; MRI, magnetic resonance imaging; OCD, obsessive-compulsive disorder; PD, Parkinson's disease; SD, standard deviation; SR, SceneRay; TS, Tourette's syndrome; UPDRS-III, Unified Parkinson's Disease Rating Scale – Part III; Y-BOCS, Yale-Brown Obsessive-Compulsive Scale; YGTSS, Yale Global Tic Severity Scale.

Table S2: Patient-wise demographic and clinical characteristics of the dystonia discovery cohorts.

SAN FRANCISCO (n = 12)

Patient Nr.	Age at Surgery (years)	Sex	Disease Duration at Surgery (years)	BFMDRS Baseline (total)	BFMDRS Postop. at FU (total)	BFMDRS Improvement Postop. at FU (%)	Time of FU (months)	Symptom Sites	Genetic Mutation	Postop. Imaging Modality
1	45	F	8	19	2	89	6	oromandibular, cervical	none	MRI
2	48	M	7	14	14	0	6	cervical	none	MRI
3	45	F	2	34	1	97	6	cervical, trunk	none	MRI
4	40	F	9	8	3	63	6	cervical	none	MRI
5	55	F	3	12	3	75	6	face, neck	none	MRI
6	48	F	4	7	4	43	6	neck	none	MRI
7	52	F	32	10	4	60	6	arms	DYT-1 mutation	MRI
8	17	M	6	11,5	3,5	70	6	arms, neck	DYT-1 mutation	MRI
9	66	M	3	22	12,5	43	6	cervical	none	MRI
10	68	F	10	17	5,5	68	6	mouth, neck, and left hand	none	MRI
11	16	M	4	35	10	71	6	right arm, neck	DYT-1 mutation	MRI
12	53	M	26	13	20	-54	6	neck, arms	none	MRI

SHANGHAI (n = 44 in the main analysis, n = 58 in the supplemental analysis)

Patient Nr.	Age at Surgery (years)	Sex	Disease Duration at Surgery (years)	BFMDRS Baseline (total)	BFMDRS Postop. at FU (total)	BFMDRS Improvement Postop. at FU (%)	Time of FU (months)	Dystonia Classification	Genetic Mutation	Postop. Imaging Modality
1	62	F	3	18	4,5	75	12	segmental	none	CT
2	44	F	1,5	8	5,5	31	15	segmental	none	CT
3	26	F	2	10	1	90	11	multi-focal	none	CT
4	25	F	5,3	9	2	78	12	generalized	none	CT
5	58	M	1,5	9	1,5	83	12	segmental	none	CT
6	60	F	1,5	10	2	80	18	segmental	none	CT

7	65	F	1,5	19	7	63	18	multi-focal	none	CT
8	73	M	1	11	5	55	12	segmental	none	CT
9	66	M	10	6	0,5	92	12	focal	none	CT
10	10	M	2	26	6	77	9	generalized	DYT-1 mutation	CT
11	32	F	2	6	3	50	12	focal	none	CT
12	51	M	30	26	7	73	17	multi-focal	DYT-24 mutation	CT
13	9	M	2	34	36	-6	12	multi-focal	none	CT
14	55	M	1,2	8	2	75	84	focal	none	CT
15	39	F	1	16,5	5	70	10	segmental	none	CT
16	38	M	6	47,5	1	98	8	generalized	none	CT
17	67	M	10	19,5	11	44	11	segmental	none	CT
18	9	M	6	24	4	83	24	multi-focal	none	CT
19	16	M	1,5	16	1,5	91	12	generalized	none	CT
20	21	M	1	5	1,5	70	10	segmental	none	CT
21	68	F	8	8,5	4,5	47	48	segmental	none	CT
22	69	F	1	11	3	73	30	segmental	none	CT
23	28	M	3,5	19	5	74	6	generalized	none	CT
24	41	M	3	10,5	4	62	24	generalized	none	CT
25	47	F	8	10	0,5	95	24	segmental	none	CT
26	41	F	15	8	2	75	30	generalized	none	CT
27	24	F	12	30	1	97	15	multi-focal	none	CT
28	11	M	2	21	2	90	18	generalized	none	CT
29	34	M	28	24	3	88	72	generalized	none	CT
30	53	F	6	16,5	4	76	7	generalized	none	CT
31	56	F	1,5	6	3	50	30	focal	none	CT
32	68	F	9	5	2,5	50	11	segmental	none	CT
33	51	F	9	49	49	0	12	multi-focal	none	CT
34	22	F	21	20	13	35	24	segmental	none	CT

35	17	M	2	92	19,5	79	24	generalized	none	CT
36	74	M	6,5	32,5	17	48	12	segmental	none	CT
37	38	M	1,5	20	7,5	63	18	generalized	none	CT
38	7	M	1	74	11	85	10	generalized	none	CT
39	9	F	0,5	16	1	94	13	multi-focal	none	CT
40	18	M	1	9	1	89	12	multi-focal	none	CT
41	39	F	15	6	1	83	8	segmental	none	CT
42	14	F	2	8	1	88	9	segmental	none	CT
43	58	F	1	6,5	1,5	77	16	segmental	none	CT
44	22	F	14	9	1	89	36	multi-focal	none	CT
Patients in the following list are included only in the supplemental analysis.										
45	66	F	2,5	3	1,5	50	12	segmental	none	CT
46	34	M	1,5	4	1	75	24	segmental	none	CT
47	12	M	1	26	14,5	44	3	segmental	none	CT
48	53	F	0,3	3,5	1,5	57	12	segmental	none	CT
49	51	F	1	6	2	67	2	focal	none	CT
50	69	F	8,5	3,5	3	14	7	segmental	none	CT
51	46	M	3,5	3,5	1	71	60	segmental	none	CT
52	34	M	3	28	3	89	4	generalized	none	CT
53	65	M	7	8	0,5	94	3	segmental	none	CT
54	62	F	4	4	1	75	12	segmental	none	CT
55	47	M	0,25	9	2	78	2	segmental	none	CT
56	74	F	3	4,5	5	-11	24	focal	none	CT
57	29	F	0,5	4	3	25	24	generalized	none	CT
58	53	F	1	4	0,5	88	13	multi-focal	none	CT

Abbreviations: BFMDRS, Burke-Fahn-Marsden Dystonia Rating Scale; CT, computed tomography; F, female; FU, follow-up; M, male; MRI, magnetic resonance imaging; Nr., number; postop., postoperative.

Table S3: Patient-wise demographic and clinical characteristics of the Tourette's syndrome discovery cohorts.

PISA/MILAN (n = 4)

Patient Nr.	Age at Surgery (years)	Sex	Disease Duration at Surgery (years)	YGTSS Baseline	YGTSS at 6 mo. Postop.	YGTSS Improvement at 6 mo. Postop. (%)	Type of Tics	Comorbidities	Postop. Imaging Modality
1	29	F	23	92	22	76	pure motor	no OCD or major depression	CT
2	26	M	19	89	20	78	pure motor	no OCD or major depression	CT
3	35	F	22	94	53	44	pure motor	no OCD or major depression	CT
4	46	M	33	82	42	49	pure motor	no OCD or major depression	CT

SHANGHAI (n = 10)

Patient Nr.	Age at Surgery (years)	Sex	Disease Duration at Surgery (years)	YGTSS Baseline	YGTSS at 6 mo. Postop.	YGTSS Improvement at 6 mo. Postop. (%)	Motor Tics: Body Regions Involved	Comorbidities	Postop. Imaging Modality
1	20	M	11	75	12	84	neck, shoulders, legs, feet	none	CT
2	14	M	9	71	21	70	eyes, neck	none	CT
3	15	M	3	68	19	72	arms, trunk, legs	none	CT
4	38	M	30	71	64	10	nose, mouth, neck, arms, hands, legs	anxiety	CT
5	18	F	12	59	8	86	eyes, nose, mouth, neck, shoulders, arms, hands	OCD, depression	CT
6	38	M	27	69	33	52	eyes, mouth, neck, shoulders, arms, hands	none	CT
7	18	M	6	76	36	53	eyes, mouth, neck, hands, legs	none	CT
8	26	M	16	63	17	73	eyes, nose, shoulders, trunk	OCD	CT
9	30	M	25	91	35	62	neck, shoulders, arms, legs, feet	OCD	CT
10	14	M	9	54	38	30	eyes, mouth, neck	none	CT

Abbreviations: CT, computed tomography; F, female; M, male; mo., months; Nr., number; OCD, obsessive-compulsive disorder; postop., postoperative; YGTSS, Yale Global Tic Severity Scale.

Table S4: Patient-wise demographic and clinical characteristics of the Parkinson's disease discovery cohorts.

Pat. Nr.	Age at Surgery (years)	Sex	Disease Duration at Surgery (years)	PD Pheno-type*	UPDRS-III Preop. Baseline (OFF Medication)	UPDRS-III Preop. Baseline (ON Medication)	UPDRS-III Postop. (STIM OFF at 12 mo.)	UPDRS-III Postop. (STIM ON at 12 mo.)	UPDRS-III Improvement ON vs. OFF STIM at 12 mo. Postop. (%)	LEDD Baseline (OFF DBS)	LEDD at 12 mo. Postop. (ON DBS)	LEDD Reduction (%)	Improvement Levodopa Response (%)	Postop. Imaging Modality
**BERLIN (n = 51)**														
1	71	F	8	1	20	10	29	30	-3	968	498	49	50	MRI
2	56	M	10	1	33	20	41	23	44	900	725	19	39	MRI
3	66	F	12	1	n/a	n/a	54	36	33	1000	400	60	31	MRI
4	61	M	11	1	33	11	45	30	33	855	640	25	67	MRI
5	69	M	6	1	31	18	37	19	49	1035	1115	-8	42	MRI
6	49	M	5	1	29	3	39	15	62	610	0	100	90	MRI
7	59	F	7	1	n/a	n/a	52	29	44	1150	100	91	72	MRI
8	72	F	18	1	n/a	n/a	13	11	15	1500	650	57	26	CT
9	70	M	12	1	n/a	n/a	40	20	50	1650	475	71	54	CT
10	63	M	11	1	n/a	n/a	44	26	41	900	600	33	46	MRI
11	67	M	9	1	27	9	36	22	39	1275	1305	-2	67	MRI
12	54	M	7	1	36	20	46	30	35	1620	1000	38	44	MRI
13	70	M	14	1	25	14	28	17	39	1640	1398	15	44	CT
14	70	F	n/a	1	n/a	n/a	27	29	-7	n/a	n/a	n/a	n/a	MRI
15	63	M	11	1	16	9	28	13	54	3065	400	87	44	MRI
16	68	M	10	1	32	17	29	19	34	1170	780	33	47	MRI
17	54	M	8	1	n/a	n/a	24	9	63	1000	267	73	85	MRI
18	58	M	7	1	n/a	n/a	32,5	8	75	600	50	92	79	CT
19	72	M	9	1	n/a	n/a	27	20	26	1300	700	46	43	MRI
20	72	F	5	1	n/a	n/a	24	25	-4	650	700	-8	44	MRI

21	53	M	16	1	36	16	33	6	82	700	375	46	56	MRI
22	52	M	9	1	42	13	47	10	79	2000	553	72	69	MRI
23	65	M	14	1	n/a	n/a	28	15	46	1450	900	38	76	MRI
24	63	F	7	1	n/a	n/a	53	19	64	2050	600	71	30	MRI
25	63	F	8	1	n/a	n/a	46	28	39	700	200	71	71	MRI
26	61	M	7	1	21	12	28	13	54	1125	890	21	43	MRI
27	55	M	14	1	n/a	n/a	53	23	57	600	0	100	44	MRI
28	59	F	16	1	n/a	n/a	26	13	50	900	300	67	65	MRI
29	53	M	16	2	48	18	53	17	68	2438	423	83	63	MRI
30	57	M	13	1	n/a	n/a	59	14	76	1150	75	93	50	MRI
31	55	F	6	1	43	10	42	35	17	930	n/a	n/a	77	MRI
32	59	M	9	1	n/a	n/a	65	21	68	950	700	26	50	MRI
33	59	M	10	1	n/a	n/a	24	19	21	800	800	0	n/a	MRI
34	57	F	10	1	n/a	n/a	42	13	69	700	200	71	64	MRI
35	66	M	21	1	29	8	57	36	37	850	300	65	72	MRI
36	42	M	5	1	n/a	n/a	47	17	64	500	0	100	62	MRI
37	49	F	12	1	n/a	n/a	36	14	61	683	150	78	n/a	MRI
38	47	F	8	1	n/a	n/a	29	18	38	1400	0	100	52	MRI
39	58	M	7	1	n/a	n/a	21	9	57	333	50	85	n/a	CT
40	51	M	14	2	42	n/a	44	11	75	875	200	77	n/a	MRI
41	67	M	15	1	28	11	25	7	72	1328	360	73	61	CT
42	61	M	10	1	34	14	33	11	67	1100	720	35	59	MRI
43	67	M	17	2	60	21	71	40	44	1453	n/a	n/a	65	MRI
44	51	F	9	1	n/a	n/a	67	39	42	1200	375	69	n/a	MRI
45	43	F	12	2	n/a	n/a	40	27	33	1025	500	51	53	MRI
46	63	M	16	1	n/a	n/a	39	30	23	500	650	-30	31	CT

47	55	M	11	1	n/a	n/a	32	10	69	400	0	100	32	MRI
48	62	F	10	1	37	12	35	26	26	945	688	27	68	MRI
49	75	M	6	2	18	16	33	21	36	400	800	-100	11	MRI
50	54	F	5	1	n/a	n/a	45	13	71	750	50	93	25	MRI
51	53	M	6	3	13	7	18	20	-11	515	600	-17	46	MRI
**WÜRZBURG (n = 43)**														
1	53	M	13	1	78	46	78	36	54	1280	380	70	41	CT
2	60	M	13	1	53	25	53	28	47	1700	0	100	53	CT
3	57	M	20	1	43	12	43	15	65	2400	300	88	72	CT
4	70	M	16	1	49	18	49	30	39	1333	300	77	63	CT
5	50	F	21	1	36	3	36	33	8	1650	200	88	92	CT
6	59	F	15	1	43	13	43	25	42	1000	450	55	70	CT
7	65	M	15	1	43	8	43	23	47	1400	700	50	81	CT
8	70	M	16	1	55	21	55	40	27	2000	1050	48	62	CT
9	55	M	6	1	56	30	56	38	32	1275	850	33	46	CT
10	63	F	9	1	56	15	56	40	29	1800	700	61	73	CT
11	47	F	10	1	73	39	73	22	70	290	0	100	47	CT
12	70	M	13	1	71	36	71	40	44	1600	800	50	49	CT
13	70	F	12	1	47	14	47	14	70	1100	200	82	70	CT
14	63	F	12	1	31	10	31	36	-16	1450	950	34	68	CT
15	59	F	15	1	50	15	50	26	48	650	350	46	70	CT
16	74	F	17	1	59	15	59	14	76	1400	500	64	75	CT
17	47	M	23	1	52	29	52	12	77	1100	100	91	44	CT
18	58	M	6	1	43	10	43	13	70	2350	950	60	77	CT
19	66	F	14	1	60	19	60	17	72	500	300	40	68	CT
20	64	M	9	1	87	18	67	27	60	2000	950	53	79	CT

21	64	M	10	1	29	14	29	32	-10	1350	900	33	52	CT
22	55	F	6	1	47	18	47	20	57	2850	250	91	62	CT
23	60	M	18	1	40	10	40	31	23	1700	900	47	75	CT
24	66	M	14	1	51	38	51	23	55	1100	450	59	25	CT
25	53	M	5	1	26	3	26	5	81	650	400	38	88	CT
26	50	M	11	1	46	11	46	17	63	1100	200	82	76	CT
27	60	M	11	1	43	24	43	22	49	1000	150	85	44	CT
28	54	M	19	1	50	11	50	15	70	1800	600	67	78	CT
29	55	M	10	1	69	14	69	25	64	2600	550	79	80	CT
30	52	F	11	1	55	4	55	14	75	1320	980	26	93	CT
31	73	M	18	1	57	16	57	29	49	2020	688	66	72	CT
32	46	M	21	1	64	41	64	48	25	2320	813	65	36	CT
33	74	M	13	1	44	16	44	45	-2	1680	1640	2	64	CT
34	68	F	6	1	64	31	64	23	64	1033	460	55	52	CT
35	71	M	9	1	38	35	38	7	82	270	0	100	8	CT
36	61	M	11	3	38	22	38	19	50	988	105	89	42	CT
37	67	M	11	1	40	18	40	24	40	2040	1880	8	55	CT
38	46	M	13	1	44	23	44	24	45	1300	800	38	48	CT
39	47	M	9	1	37	22	37	22	41	1000	413	59	41	CT
40	63	M	10	1	67	18	67	39	42	2395	832	65	73	CT
41	61	M	9	1	52	23	52	19	63	916	733	20	56	CT
42	69	M	7	1	46	26	46	7	85	1020	443	57	43	CT
43	66	M	12	1	31	2	31	13	58	1068	0	100	94	CT

Notes: *Classification into Parkinson's disease (PD) phenotypes according to Schiess et al (2000) [26]: 1 = axial-rigid type, 2 = mixed type, 3 = tremor dominant type. *Abbreviations*: CT, computed tomography; F, female; LEDD, levodopa equivalent daily dosage; M, male; Mo., months; MRI, magnetic resonance imaging; Nr., number; postop., postoperative; preop., preoperative; STIM, stimulation; UPDRS-III, Unified Parkinson's Disease Rating Scale – Part III.

Table S5: Patient-wise demographic and clinical characteristics of the obsessive-compulsive disorder discovery cohorts.

GRENOBLE (n = 13)

Patient Nr.	Age at Surgery (years)	Sex	Disease Duration at Surgery (years)	Y-BOCS Baseline	Y-BOCS at 12 mo. Postop.	Y-BOCS Improvement at 12 mo. Postop. (%)	OCD Subtype	Comorbidities	Postop. Imaging Modality
1	35	M	13	32	26	19	checking, repeating	none	MRI
2	36	M	17	32	28	13	washing, ordering	anankastic personality	MRI
3	36	F	18	25	12	52	washing	none	MRI
4	30	F	24	34	30	12	checking	skin picking, hypothyroidia	MRI
5	51	F	25	40	16	60	washing	none	MRI
6	45	M	26	33	32	3	checking, hoarding	social phobia	MRI
7	53	F	22	33	2	94	checking	depression, Minkowski-Chauffard syndrome	MRI
8	37	F	11	36	6	83	washing	none	MRI
9	27	M	10	38	22	42	checking, washing	hypersomnia	CT
10	50	F	39	35	25	29	checking	None	CT
11	40	F	15	36	30	17	washing	history of eating disorder, history of one seizure, alcoholism, pruritus	MRI
12	37	F	5	32	3	91	checking	none	MRI
13	32	F	21	30	14	53	checking	dermatillomania	CT

LONDON (n = 6)

Patient Nr.	Age at Surgery (years)	Sex	Disease Duration at Surgery (years)	Y-BOCS Baseline	Y-BOCS after 'amSTN-only' Phase[+]	Y-BOCS Improvement at FU (%)	OCD Subtype	Comorbidities	Postop. Imaging Modality
1	38	F	22	38	32* at 6 mo.	16	washing, repeating	none	MRI
2	38	M	22	34	26 at 6 mo.	23	ordering, checking, repeating	body dysmorphic disorder	MRI
3	62	M	30	37	17 at 6 mo.	55	washing, checking, repeating	none	MRI

4	37	M	20	38	20 at 3 mo.	47	washing, checking, ordering	recurrent depression	MRI
5	55	M	23	34	23 at 3 mo.	32	hoarding	recurrent depression	MRI
6	43	M	28	36	1 at 3 mo.	97	checking	generalized anxiety disorder, depression	MRI

Notes: *Indicates last entry carried forward. [+] Obsessive-compulsive disorder (OCD) patients from London were bilaterally implanted with deep brain stimulation (DBS) electrodes to two different target sites (four electrodes per patient) – the anteromedial subthalamic nucleus (amSTN) and the ventral capsule/ventral striatum (VC/VS). Patients were randomly assigned to either receive 'pure amSTN' for the first three months or from months four to six postoperatively (and 'pure VC/VS' for the remainder of the first six months). Model set-up (based on the discovery cohort) was informed on stimulation parameters and corresponding clinical scores taken after the amSTN phase, while model validation was performed based on VC/VS-stimulation related data points. AmSTN stimulation was applied via a Medtronic (MDT) model of type 3389, while MDT 3387 leads were implanted for stimulation of the VC/VS target zone (considered only for model validations, see Tables S6 and S8 below). *Abbreviations:* amSTN, anteromedial subthalamic nucleus; CT, computed tomography; F, female; FU, follow-up; M, male; mo., months; MRI, magnetic resonance imaging; Nr., number; postop., postoperative; Y-BOCS, Yale-Brown Obsessive-Compulsive Scale.

Table S6. Summary of demographic and clinical patient characteristics within each retrospective model validation cohort.

Disease cohort	PD	OCD		
**Demographic Information**				
**Cohort**	Würzburg	Boston	Cologne	London
**Surgical DBS center**	University Hospital Würzburg	Massachusetts General Hospital	University Hospital Cologne	National Hospital for Neurology and Neurosurgery
**n (females)**	32 (10)	7 (4)	22 (13)	6 (1)
**Age at time of surgery (mean ± SD; range; in years)**	58.00 ± 7.73; 46-79	38.86 ± 16.80; 21-64	42.20 ± 13.38; 21-64	45.50 ± 10.52; 37-62
**Disease duration at time of surgery (mean ± SD; range; in years)**	10.06 ± 4.21; 3-20	25.14 ± 14.21; 10-44	25.27 ± 12.29; 6-49	24.17 ± 3.92; 20-30
**Clinical Outcome**				
**Surgical target**	STN	VC/VS	VC/VS	VC/VS
**Main clinical outcome assessment**	UPDRS-III [13] (total score; range 0-199, with higher scores corresponding to higher symptom burden)	Y-BOCS [15] (total score; range 0-40, with higher scores corresponding to higher symptom burden)	Y-BOCS [15] (total score; range 0-40, with higher scores corresponding to higher symptom burden)	Y-BOCS [15] (total score; range 0-40, with higher scores corresponding to higher symptom burden)
**Time of FU (in months)**	mean: 11.5 ± 3.08 SD; range: 6-22	18	12	3 or 6 (post-VC/VS phase)[+]
**Comparison basis for calculation of improvement values (baseline to DBS stimulation ON conditions)**	Postoperative ON vs. OFF stimulation (ON dopaminergic medication)	Pre- to postoperative	Pre- to postoperative	Pre- to postoperative
**Score at baseline (mean ± SD)**	45.84 ± 10.93	33.57 ± 2.37	31.12 ± 4.30	36.17 ± 1.83

**Score at time of FU under stimulation ON condition (mean ± SD)**	24.16 ± 10.65	20.29 ± 10.40	20.70 ± 7.70	17.00 ± 8.74
**Relative improvement (mean ± SD; in %)**	46.90 ± 21.11	40.13 ± 29.84	31.00 ± 20.5	52.60 ± 25.51
**Absolute improvement (mean ± SD)**	21.69 ± 10.96	13.29 ± 9.55	9.60 ± 6.50	19.17 ± 9.39
		**Imaging and DBS Specifications**		
**Imaging modality (postop.)**	CT (n = 32)	CT (n = 7)	CT (n = 22)	MRI (n = 6)
**Electrode models**	BSV (n = 23) or BSV Directed (n = 9)	MDT 3387 (n = 7)	MDT 3387 (n = 3) or MDT 3389 (n = 19)	MDT 3387 (n = 6)
**Related citations**	Butenko et al., 2022 [27]	McLaughlin et al., 2021 [28]	Baldermann et al., 2019 [29]; Li et al., 2020 [3]; 2021 [30]	Tyagi et al., 2019 [24]

Notes: [+] Obsessive-compulsive disorder (OCD) patients from London were bilaterally implanted with deep brain stimulation (DBS) electrodes to two different target sites (four electrodes per patient) – the anteromedial subthalamic nucleus (amSTN) and the ventral capsule/ventral striatum (VC/VS). Patients were randomly assigned to either receive 'pure VC/VS' for the first three months or from months four to six postoperatively (and 'pure amSTN' for the remainder of the first six months). Model set-up (based on the discovery cohort) was informed on stimulation parameters and corresponding clinical scores taken after the amSTN phase, while model validation was performed based on VC/VS-stimulation related data points. VC/VS stimulation was applied via a Medtronic (MDT) model of type 3387 (considered only for model validations), while MDT 3389 leads were implanted for stimulation of the amSTN target zone (considered only for the model set-up stage, see Tables S1 and S5 above). *Abbreviations:* BSV, Boston Scientific Vercise; CT, computed tomography; DBS, deep brain stimulation; FU, follow-up; MRI, magnetic resonance imaging; PD, Parkinson's disease; postop., postoperative; SD, standard deviation; UPDRS-III, Unified Parkinson's Disease Rating Scale – Part III; Y-BOCS, Yale-Brown Obsessive-Compulsive Scale.

Table S7: Patient-wise demographic and clinical characteristics of the Parkinson's disease validation cohort.

Pat. Nr.	Age at Surgery (years)	Sex	Disease Duration at Surgery (years)	UPDRS-III Total Preop. Baseline (OFF Medication)	UPDRS-III Total Preop. Baseline (ON Medication)	UPDRS-III Total Postop. (STIM ON at FU)	UPDRS-III Total Improvement ON vs. OFF STIM at FU (%)	LEDD Baseline (OFF DBS)	Time of FU (months)	Postop. Imaging Modality
**WÜRZBURG (n = 32)**										
1	57	M	12	48	9	17	65	81	12	CT
2	50	F	9	62	27	38	39	56	12	CT
3	62	F	12	42	22	17	60	48	12	CT
4	50	M	18	52	34	34	35	35	10	CT
5	60	M	7	56	9	17	70	84	16	CT
6	60	M	12	49	20	24	51	59	12	CT
7	73	M	10	40	20	28	30	50	22	CT
8	58	F	11	44	10	8	82	77	12	CT
9	64	M	5	45	12	27	40	73	12	CT
10	55	F	3	61	34	39	36	44	12	CT
11	62	M	11	45	11	32	29	76	12	CT
12	54	F	6	31	10	16	48	68	12	CT
13	50	M	12	47	20	36	23	57	12	CT
14	49	M	7	44	20	14	68	55	13	CT
15	46	M	7	38	13	9	76	66	12	CT
16	67	M	8	45	5	48	-7	89	13	CT
17	52	M	9	50	13	26	48	74	12	CT
18	46	M	9	44	17	21	52	61	12	CT
19	64	M	6	39	18	35	10	54	12	CT
20	53	M	8	16	8	10	38	50	12	CT

21	58	M	10	52	38	24	54	27	12	CT
22	57	F	18	41	14	22	46	66	12	CT
23	68	F	17	45	18	7	84	60	12	CT
24	64	M	9	44	23	19	57	48	12	CT
25	79	M	5	42	26	25	40	38	12	CT
26	61	F	16	39	14	28	28	64	12	CT
27	56	M	8	42	33	39	7	21	12	CT
28	47	F	12	40	19	20	50	53	6	CT
29	58	M	20	85	42	42	51	51	6	CT
30	60	M	13	51	25	20	61	51	6	CT
31	52	F	6	41	7	14	66	83	6	CT
32	64	M	6	47	22	17	64	53	6	CT

Abbreviations: CT, computed tomography; F, female; FU, follow-up; LEDD, levodopa equivalent daily dosage; M, male; MRI, magnetic resonance imaging; Nr., number; postop., postoperative; preop., preoperative; STIM, stimulation; UPDRS-III, Unified Parkinson's Disease Rating Scale – Part III.

Table S8: Patient-wise demographic and clinical characteristics of the obsessive-compulsive disorder validation cohorts.

BOSTON (n = 7)

Patient Nr.	Age at Surgery (years)	Sex	Disease Duration at Surgery (years)	Y-BOCS Baseline	Y-BOCS at 18 mo. Postop.	Y-BOCS Improvement at 18 mo. Postop. (%)	OCD Subtype	Comorbidities	Postop. Imaging Modality
1	58	M	41	32	0	100	doubt, checking	n/a	CT
2	25	F	13	35	16	54	doubt, checking	n/a	CT
3	64	F	44	32	21	34	contamination, cleaning	n/a	CT
4	21	M	10	33	29	12	doubt, checking	n/a	CT
5	26	F	11	34	22	35	doubt, checking	n/a	CT
6	42	F	27	31	22	29	contamination, cleaning	n/a	CT
7	36	M	30	38	32	16	contamination, cleaning	n/a	CT

COLOGNE (n = 22)

Patient Nr.	Age at Surgery (years)	Sex	Disease Duration at Surgery (years)	Y-BOCS Baseline	Y-BOCS at 12 mo. Postop.	Y-BOCS Improvement at 18 mo. Postop. (%)	OCD Subtype	Comorbidities	Postop. Imaging Modality
1	36	M	24	30	8	73	magical thinking, incompleteness, responsibility	recurrent major depressive disorder (moderate)	CT
2	32	M	15	34	34	0	washing, contamination	none	CT
3	36	M	27	33	24	27	washing, contamination, symmetry, catastrophic fears	none	CT
4	54	M	6	34	12	65	washing, controlling, contamination fears, aggressive thought	none	CT
5	27	M	22	27	14	48	contamination, aggressive impulses, symmetry	none	CT
6	21	M	18	25	20	20	repetition, keeping order, mental exactness, checking	none	CT
7	25	F	15	35	23	34	bodydysmorphobia, checking, magical thinking	hypochondriasis	CT

8	49	M	24	32	22	31	somatic obsessions, contamination fears	none	CT
9	34	M	19	37	34	8	cleanliness, washing, contamination fears, disgust	none	CT
10	47	F	19	28	13	54	checking, aggressive fears, responsibility	none	CT
11	59	F	49	36	22	39	aggressive thoughts/impulses, mental urges	recurrent major depressive disorder (severe without psychotic features)	CT
12	27	F	6	26	16	38	aggressive impulses, checking, doubting	none	CT
13	64	F	20	25	18	28	catastrophic fears, responsibility	anxiety disorder (not otherwise specified); recurrent major depressive disorder (moderate)	CT
14	56	M	35	35	25	29	catastrophic fears, responsibility	recurrent major depressive disorder (moderate)	CT
15	29	M	18	33	22	33	aggressive impulses, responsibility	recurrent major depressive disorder (severe without psychotic features)	CT
16	60	M	44	25	20	20	aggressive impulses, responsibility	recurrent major depressive disorder (severe without psychotic features)	CT
17	39	F	29	30	28	7	magical thinking, incompleteness, mental urges	recurrent major depressive disorder (moderate)	CT
18	41	F	34	26	21	19	aggressive impulses, responsibility, checking	none	CT
19	45	F	39	37	27	27	mental rituals/urges, magical thinking	none	CT
20	54	M	42	34	29	15	contamination, cleanliness	none	CT
21	36	M	11	36	36	0	responsibility, catastrophic fears, checking	recurrent major depressive disorder (moderate)	CT

22	56	F	40	32	11	66	somatic obsessions, sexual obsessions	sedative, hypnotic, or anxiolytic dependence	CT
**LONDON (n = 6)**									
**Patient Nr.**	**Age at Surgery (years)**	**Sex**	**Disease Duration at Surgery (years)**	**Y-BOCS Baseline**	**Y-BOCS after 'VC/VS only' Phase[+]**	**Y-BOCS Improvement at FU (%)**	**OCD Subtype**	**Comorbidities**	**Postop. Imaging Modality**
1	38	F	22	38	22 at 3 mo.	42	washing, repeating	none	MRI
2	38	M	22	34	29 at 3 mo.	15	ordering, checking, repeating	body dysmorphic disorder	MRI
3	62	M	30	37	18 at 3 mo.	51	washing, checking, repeating	none	MRI
4	37	M	20	38	13 at 6 mo.	66	washing, checking, ordering	recurrent depression	MRI
5	55	M	23	34	17 at 6 mo.	50	hoarding	recurrent depression	MRI
6	43	M	28	36	3 at 6 mo.	92	checking	generalized anxiety disorder, depression	MRI

Notes: [+] Obsessive-compulsive disorder (OCD) patients from London were bilaterally implanted with deep brain stimulation (DBS) electrodes to two different target sites (four electrodes per patient) – the anteromedial subthalamic nucleus (amSTN) and the ventral capsule/ventral striatum (VC/VS). Patients were randomly assigned to either receive 'pure VC/VS' for the first three months or from months four to six postoperatively (and 'pure amSTN' for the remainder of the first six months). Model set-up (based on the discovery cohort) was informed on stimulation parameters and corresponding clinical scores taken after the amSTN phase, while model validation was performed based on VC/VS-stimulation related data points. VC/VS stimulation was applied via a Medtronic (MDT) model of type 3387 (considered only for model validations), while MDT 3389 leads were implanted for stimulation of the amSTN target zone (considered only for the model set-up stage, see Tables S1 and S5 above). *Abbreviations:* CT, computed tomography; F, female; FU, follow-up; M, male; mo., months; MRI, magnetic resonance imaging; Nr., number; postop., postoperative; Y-BOCS, Yale-Brown Obsessive-Compulsive Scale.

Table S9: Peak voxel coordinates of subthalamic sweet spots and of disease-specific cortical sites of interconnection with sweet tracts.

		DYT			TS			PD			OCD		
		**(x**	**y**	**z)**	**(x**	**y**	**z)**	**(x**	**y**	**z)**	**(x**	**y**	**z)**
**Cortical sites of interconnection with sweet tracts (in mm)**	*Left*	(-22.00	-20.00	72.00)	(-3.32	-29.65	-45.59)	(-14.00	2.00	70.00)	(-8.23	-10.29	-16.50)
	Right	(23.50	-18.75	72.00)	(10.00	-12.00	-16.50)	(10.00	2.00	70.00)	(7.88	-9.50	-16.50)
**Subthalamic sweet spots (in mm)**	*Left*	(-14.72	-14.38	-4.10)	(-12.96	-11.08	-6.30)	(-12.08	-13.94	-6.74)	(-8.78	-11.52	-9.60)
	Right	(14.54	-13.50	-4.30)	(13.00	-9.98	-6.08)	(11.90	-13.28	-6.74)	(8.38	-10.86	-9.38)

Abbreviations: DYT, dystonia; mm, millimeters; OCD, obsessive-compulsive disorder; PD, Parkinson's disease; TS, Tourette's syndrome.

Table S10: Overlap between connected streamlines shared among disorders and disease-specific sweet streamlines.

	DYT	PD	TS	OCD
**Overlap (%)**	4.41	2.21	1.75	5.42
**Four-sample test for equality of proportions**		p < 2.2e-16		
Pairwise test for equality of proportions (two-sided tests)				
**DYT**				
**PD**	p < 2.2e-16			
**TS**	p < 2.2e-16	p = 2.835e-14		
**OCD**	p = 1.353e-11	p < 2.2e-16	p < 2.2e-16	

Abbreviations: DYT, dystonia; mm, millimeters; OCD, obsessive-compulsive disorder; PD, Parkinson's disease; TS, Tourette's syndrome.

Table S11: Patient-wise demographic and clinical characteristics of patient cases for prospective model validations.

PD

Age at Surgery (years)	Sex	Disease Duration at Surgery (years)	PD Phenotype	UPDRS-III Total at 3 Mo. Postop. (STIM OFF, Med. OFF)	UPDRS-III Total at 3 Mo. Postop. (STIM ON under Clinical Settings, Med. OFF)	UPDRS-III Total at 3 Mo. Postop. (STIM ON under Model-Based Settings, Med. OFF)	UPDRS-III Total Improvement ON vs OFF STIM at 3 Mo. Postop. (under Clinical Settings, Med. OFF, %)	UPDRS-III Total Improvement ON vs OFF STIM at 3 Mo. Postop. (under Model-Based Settings, Med. OFF, %)	Electrode Model	Clinical Settings	Model-Based Settings	Postop. Imaging Modality
PD patient implanted to the STN at Würzburg University Hospital (Würzburg, Germany)												
67	M	9	Akinetik-rigid	35	14	10	60	71	BSV Directed	left hem.: C+, 1- (60%), 2- (40%) / 2.4 mA / 60 µs / 130 Hz; right hem..: C+, 1- (20%), 2- (20%), 3- (3%), 4- (57%) / 2.6 mA / 60 µs / 130 Hz	left hem.: C+, 4- (70%), 7- (30%) / 3 mA / 60 µs / 130 Hz; right hem.: C+, 7- (30%), 8- (70%) / 3 mA / 60 µs / 130 Hz	CT

OCD

Age at Surgery (years)	Sex	Disease Duration at Surgery (years)	OCD Phenotype	Comorbi-dities	Previous Treatments	Y-BOCS Total Preop.	Y-BOCS Total at 1 Mo. Postop. (STIM ON under Clinical Settings)	Y-BOCS Total at 1 Mo. Postop. (STIM ON under Model-Based Settings)	Y-BOCS Total Improvement Preop. vs. STIM ON at 1 Mo. Postop. (under Clinical Settings, %)	Y-BOCS Total Improvement Preop. Vs. STIM ON at 1 Mo. Postop. (under Model-Based Settings, %)	Electrode Model	Clinical Settings	Model-Based Settings	Postop. Imaging Modality
OCD patient implanted to the VC/VS region at Massachusetts General Hospital (Boston, USA)														
21	F	13	obsessions about food and water intake, compulsions involving	major depressive disorder	11 different medication trials (including multiple SSRIs, clomipramine,	35	29	22	17	37	MDT 3387	left hem.: C+, 1- , 2- / 3 mA / 140 µs / 130 Hz;	left hem.: C+, 2- / 4.2 mA / 80 µs / 130 Hz;	CT

			ingestion events, skin picking		and ketamine), intensive ERP, ECT							right hem.: C+, 9- / 2.4 mA / 140 µs / 130 Hz;	right hem.: C+, 10- / 3.4 mA / 80 µs / 130 Hz;	
OCD patient implanted to the STN at Clínica de Dor e Funcional (São Paulo, Brazil)														
32	M	14	compulsions of noting words unknown to the patient and transcribing their meaning from dictionaries, death-related intrusions leading to compulsive religious rituals	apathy, major depressive disorder	48 sessions of ECT, CBT, biofeedback, mindfulness training, 3 different protocols of TMS with over 50 sessions	26	-	6	-	77	BSV	-	left hem.: C+, 1- (100%) / 3 V / 90 µs / 130 Hz; right hem.: C+, 9- (100%) / 3 V / 90 µs / 130 Hz;	CT

Abbreviations: BSV, Boston Scientific Vercise; CBT, cognitive behavioral therapy; CT, computed tomography; ECT, electroconvulsive therapy; ERP, exposure and response prevention therapy; F, female; hem., hemisphere; Hz, Hertz; M, male; mA, milliampere; Mo., months; MDT, Medtronic; postop., postoperative; preop., preoperative; postop., postoperative; SSRI, selective serotonin reuptake inhibitor; STIM, stimulation; STN, subthalamic nucleus; TMS, transcranial magnetic stimulation; UPDRS-III, Unified Parkinson's Disease Rating Scale – Part III; VC/VS, ventral capsule/ventral striatum; V, volt; Y-BOCS, Yale-Brown Obsessive-Compulsive Scale.

References

1. Ewert, S. *et al.* Toward defining deep brain stimulation targets in MNI space: A subcortical atlas based on multimodal MRI, histology and structural connectivity. *Neuroimage* **170**, 271–282 (2018).
2. Edlow, B. L. *et al.* 7 Tesla MRI of the ex vivo human brain at 100 micron resolution. *Sci. Data* **6**, 244 (2019).
3. Li, N. *et al.* A unified connectomic target for deep brain stimulation in obsessive-compulsive disorder. *Nat. Commun.* **11**, 3364 (2020).
4. Amunts, K. *et al.* BigBrain: An ultrahigh-resolution 3D human brain model. *Science (80-.).* **340**, 1472–1475 (2013).
5. Irmen, F. *et al.* Left prefrontal connectivity links subthalamic stimulation with depressive symptoms. *Ann. Neurol.* **87**, 962–975 (2020).
6. Van Essen, D. C. *et al.* The WU-Minn Human Connectome Project: An overview. *Neuroimage* **80**, 62–79 (2013).
7. Wang, F. *et al.* In vivo human whole-brain Connectom diffusion MRI dataset at 760 µm isotropic resolution. *Sci. Data* **8**, 122 (2021).
8. Petersen, M. V *et al.* Holographic reconstruction of axonal pathways in the human brain. *Neuron* **104**, 1056-1064.e3 (2019).
9. Marek, K. *et al.* The Parkinson Progression Marker Initiative (PPMI). *Prog. Neurobiol.* **95**, 629–635 (2011).
10. Oxenford, S. *et al.* WarpDrive: Improving spatial normalization using manual refinements. *Med. Image Anal.* **91**, 103041 (2024).
11. Neudorfer, C. *et al.* Lead-DBS v3.0: Mapping deep brain stimulation effects to local anatomy and global networks. *Neuroimage* **268**, 119862 (2023).
12. Burke, R. E. *et al.* Validity and reliability of a rating scale for the primary torsion dystonias. *Neurology* **35**, 73–77 (1985).
13. Goetz, C. G. *et al.* Movement disorder society-sponsored revision of the unified Parkinson's disease rating scale (MDS-UPDRS): Process, format, and clinimetric testing plan. *Mov. Disord.* **22**, 41–47 (2007).
14. Leckman, J. F. *et al.* The Yale Global Tic Severity Scale: Initial testing of a clinician-rated scale of tic severity. *J. Am. Acad. Child Adolesc. Psychiatry* **28**, 566–573 (1989).
15. Goodman, W. K. *et al.* The Yale-Brown Obsessive Compulsive Scale: I. Development, use, and reliability. *Arch. Gen. Psychiatry* **46**, 1006–1011 (1989).
16. Ostrem, J. L. *et al.* Subthalamic nucleus deep brain stimulation in primary cervical dystonia. *Neurology* **76**, 870–878 (2011).
17. Ostrem, J. L. *et al.* Subthalamic nucleus deep brain stimulation in isolated dystonia: A 3-year follow-up study. *Neurology* **88**, 25–35 (2016).

18. Lin, S. *et al.* Deep brain stimulation of the globus pallidus internus versus the subthalamic nucleus in isolated dystonia. *J. Neurosurg.* **132**, 721–732 (2019).
19. He, W. *et al.* Weight change after subthalamic nucleus deep brain stimulation in patients with isolated dystonia. *Front. Neurol.* **12**, 1–8 (2021).
20. Horn, A. *et al.* Connectivity predicts deep brain stimulation outcome in Parkinson's disease. *Ann. Neurol.* **82**, 67–78 (2017).
21. Horn, A. *et al.* Deep brain stimulation induced normalization of the human functional connectome in Parkinson's disease. *Brain* **142**, 3129–3143 (2019).
22. Vissani, M. *et al.* Spatio-temporal structure of single neuron subthalamic activity identifies DBS target for anesthetized Tourette Syndrome patients. *J. Neural Eng.* **16**, 066011 (2019).
23. Dai, L. *et al.* Subthalamic deep brain stimulation for refractory Gilles de la Tourette' s syndrome: Clinical outcome and functional connectivity. *J. Neurol.* **269**, 6116–6126 (2022).
24. Tyagi, H. *et al.* A randomized trial directly comparing ventral capsule and anteromedial subthalamic nucleus stimulation in obsessive-compulsive disorder: Clinical and imaging evidence for dissociable effects. *Biol. Psychiatry* **85**, 726–734 (2019).
25. Polosan, M. *et al.* Affective modulation of the associative-limbic subthalamic nucleus: deep brain stimulation in obsessive–compulsive disorder. *Transl. Psychiatry* **9**, (2019).
26. Schiess, M. C., Zheng, H., Soukup, V. M., Bonnen, J. G. & Nauta, H. J. W. Parkinson's disease subtypes: Clinical classification and ventricular cerebrospinal fluid analysis. *Park. Relat. Disord.* **6**, 69–76 (2000).
27. Butenko, K. *et al.* Linking profiles of pathway activation with clinical motor improvements – A retrospective computational study. *NeuroImage Clin.* **36**, 103185 (2022).
28. McLaughlin, N. C. R. *et al.* Double blind randomized controlled trial of deep brain stimulation for obsessive-compulsive disorder: Clinical trial design. *Contemp. Clin. Trials Commun.* **22**, 100785 (2021).
29. Baldermann, J. C. *et al.* Connectivity profile predictive of effective deep brain stimulation in obsessive-compulsive disorder. *Biol. Psychiatry* **85**, 735–743 (2019).
30. Li, N. *et al.* A unified functional network target for deep brain stimulation in obsessive-compulsive disorder. *Biol. Psychiatry* **90**, 701–713 (2021).
