# Supplementary material for: Mapping dysfunctional circuits in the frontal cortex using deep brain stimulation
Source: Nat Neurosci. 2024 Feb 22;27(3):573–86. doi: 10.1038/s41593-024-01570-1 (PMC10917675; doi:10.1038/s41593-024-01570-1)
Supplement: Supplementary file 2 — Reporting Summary [file 41593_2024_1570_MOESM2_ESM.pdf]

nature portfolio

Corresponding author(s): Andreas Horn, MD PhD; Ningfei Li, PhD

Last updated by author(s): 01/26/2024

Reporting Summary

Nature Portfolio wishes to improve the reproducibility of the work that we publish. This form provides structure for consistency and transparency in reporting. For further information on Nature Portfolio policies, see our Editorial Policies and the Editorial Policy Checklist.

Statistics

For all statistical analyses, confirm that the following items are present in the figure legend, table legend, main text, or Methods section.

n/a	Confirmed	
☐	☒	The exact sample size (*n*) for each experimental group/condition, given as a discrete number and unit of measurement
☐	☒	A statement on whether measurements were taken from distinct samples or whether the same sample was measured repeatedly
☐	☒	The statistical test(s) used AND whether they are one- or two-sided<br>*Only common tests should be described solely by name; describe more complex techniques in the Methods section.*
☒	☐	A description of all covariates tested
☐	☒	A description of any assumptions or corrections, such as tests of normality and adjustment for multiple comparisons
☐	☒	A full description of the statistical parameters including central tendency (e.g. means) or other basic estimates (e.g. regression coefficient) AND variation (e.g. standard deviation) or associated estimates of uncertainty (e.g. confidence intervals)
☐	☒	For null hypothesis testing, the test statistic (e.g. *F*, *t*, *r*) with confidence intervals, effect sizes, degrees of freedom and *P* value noted<br>*Give P values as exact values whenever suitable.*
☒	☐	For Bayesian analysis, information on the choice of priors and Markov chain Monte Carlo settings
☒	☐	For hierarchical and complex designs, identification of the appropriate level for tests and full reporting of outcomes
☐	☒	Estimates of effect sizes (e.g. Cohen's *d*, Pearson's *r*), indicating how they were calculated

Our web collection on statistics for biologists contains articles on many of the points above.

Software and code

Policy information about availability of computer code

Data collection	Clinical outcome data and deep brain stimulation (DBS) parameters were retrieved from the collecting sites using Microsoft Excel v16.70 and imported for analysis via MATLAB R2022b, v9.13.0.2105380. Optimal DBS streamline profiles and sweet spots were constructed using MATLAB scripts (openly available within the Lead-DBS environment, https://www.lead-dbs.org/), the former combined with public human connectome data, see prior studies based on the same approach (e.g., Baldermann et al., 2019, Biol. Psychiatry; Horn et al., 2022, Proc. Natl. Acad. Sci; Irmen et al., 2020, Ann. Neurol.).
Data analysis	All statistical analyses were conducted using MATLAB scripts openly and publicly available within the Lead-DBS environment (https://www.lead-dbs.org/): https://github.com/netstim/leaddbs<br><br>As described in the manuscript, all analysis approaches built upon Lead-DBS, v3.0 software and included preprocessing of patient-specific imaging (head CT/MRI), followed by electrode reconstruction (both via the Lead-DBS toolbox) as well as estimation of stimulation volumes (via the Lead-Group toolbox). Some of these processig steps build upon algorithms adapted from SPM12, Advanced Normalization Tools (ANTs), Precise and Convenient Electrode Reconstruction for Deep Brain Stimulation (PaCER), SimBio, or FieldTrip (see https://www.lead-dbs.org/about/lead-dbs-dependencies/). The Symmetric Normalization (SyN) approach included in ANTs, as pre-installed with Lead-DBS, v3.0, was used for multispectral spatial normalization into template space. Manual optimizations of the standard normalization warp-fields were based on the WarpDrive toolbox, as implemented in Lead-DBS, v3.0 (https://github.com/netstim/SlicerNetstim). Electrodes were pre-localized using the PaCER algorithm, or the trajectory search/contact reconstructions (TRAC/CORE) algorithm, both as pre-installed with Lead-DBS, v3.0 software. The electric field (E-field) was simulated via an adaptation of the SimBio/FieldTrip pipeline (https://www.mrt.uni-jena.de/simbio/; http://fieldtriptoolbox.org/), as implemented in Lead-DBS, v3.0 software. G*Power software, v 3.1.9.6 was used to determine the power to detect a presumed effect by means of our available sample sizes.

Based on Lead-DBS-internal toolboxes, patient-specific results were further processed at the group-level using DBS Sweet Spot Mapping (via the DBS Sweet Spot Mapping Explorer) and DBS Fiber Filtering (via the DBS Fiber Filtering Explorer), both as included in Lead-DBS, v3.0.

SPM12 (https://www.fil.ion.ucl.ac.uk/spm/) was used for smoothing of sweet spots and sweet streamline bundles converted to voxelized images (tract-density maps). Surf Ice software, v.1.0.20211006 (https://www.nitrc.org/projects/surfice; https://github.com/neurolabusc/surf-ice/releases) was applied to display the topographical organization of sweet spots and that of interconnected sites of sweet streamlines at the cortical level in three-dimensional space. Finally, disease-wise sweet in conjunction with sour spots were displayed individually using 3D Slicer software, v5.2.1 (https://www.slicer.org/).

Diffusion-weighted magnetic resonance imaging (dMRI) data used to create the OCD disease matched connectome were preprocessed via FMRIB Software Library (FSL), Release 6.0 (https://fsl.fmrib.ox.ac.uk/fsl/fslwiki/FSL). DSI-Studio (http://dsi-studio.labsolver.org/) was employed to construct the OCD matched connectome, as well as the DBS Tractography atlas, v2.1, which served the purpose of informing connectivity-based analyses in the present study (amongst other previously established connectomic resources).

For manuscripts utilizing custom algorithms or software that are central to the research but not yet described in published literature, software must be made available to editors and reviewers. We strongly encourage code deposition in a community repository (e.g. GitHub). See the Nature Portfolio guidelines for submitting code & software for further information.

Data

Policy information about availability of data

All manuscripts must include a data availability statement. This statement should provide the following information, where applicable:

- Accession codes, unique identifiers, or web links for publicly available datasets
- A description of any restrictions on data availability
- For clinical datasets or third party data, please ensure that the statement adheres to our policy

Patient-specific data:
The analyses described in this manuscript build on datasets of eleven patient cohorts and three prospective patient cases from a total of ten international institutions. Detailed patient-wise demographic and clinical information is available in the supplementary materials in anonymized form. Imaging data cannot be publicly shared due to patient privacy restrictions but is available from the principal investigators of the collecting sites upon reasonable request within the scope of a data sharing agreement. Inquiries for further information and data sharing requests should be directed to the corresponding authors of this manuscript (A.H., via ahorn1@bwh.harvard.edu, or N.L., via ningfei.li@gmail.com) who commit to replying to any request within a timeframe of 30 days. Anonymized E-fields of patients analyzed in this work are made publicly available alongside clinical outcome information via a dedicated open repository of the Open Science Framework (OSF) (https://osf.io/zu9c6/).

Atlas of results:
The sweet streamline and sweet spot profiles are openly available in the form of an atlas included within Lead-DBS software, v3.0 (https://www.lead-dbs.org), as well as from the above-mentioned public repository of the OSF associated with this study (https://osf.io/zu9c6/).

Normative data:
- Human Connectome Project (HCP) 985 Connectome: The processed version (as described in Li et al., 2020, Nat. Commun) is currently not openly available but can be shared by the corresponding authors upon reasonable request. Source data used to calculate this connectome are openly accessible via the repository of the HCP (https://www.humanconnectome.org/study/hcp-young-adult/document/1200-subjects-data-release).
- Massachusetts General Hospital 760 µm Connectome (Edlow et al., 2019, Sci. Data): openly available from https://datadryad.org/stash/dataset/doi:10.5061/dryad.nzs7h44q2
- Basal Ganglia Pathway Atlas (Petersen et al., 2019, Neuron): openly available from https://osf.io/mhd4z/
- DBS Tractography Atlas, v2.1: The processed version of this atlas resource is openly available (https://github.com/netstim/DBS-Tractography-Atlas.git). The HCP-1,065 diffusion source data used to inform this pathway atlas can be openly accessed via DSI-Studio (https://sites.google.com/a/labsolver.org/brain/diffusion-mri-data/hcp-dmri-data).
- Parkinson's Progression Marker Initiative (PPMI)-85 Connectome, v1.1 (Ewert et al., 2018): The processed version can be openly and publicly derived via the Lead-DBS knowledge base (https://www.lead-dbs.org/helpsupport/knowledge-base/atlasesresources/normative-connectomes/). Source data used for calculation of this connectome can be freely accessed via the homepage of the PPMI (https://www.ppmi-info.org/access-data-specimens/download-data).
- OCD-06 Connectome: The processed version of this connectome can be shared by the corresponding authors upon reasonable request. Source data of OCD patients employed to calculate this connectome cannot be publicly shared due to patient privacy restrictions but is available from the principal investigators of the collecting sites upon reasonable request within the scope of a data sharing agreement. Inquiries for further information and data sharing requests should be directed to the corresponding authors of this manuscript (A.H., via ahorn1@bwh.harvard.edu, or N.L., via ningfei.li@gmail.com) who commit to replying to any request within a timeframe of 30 days.

Cortical and subcortical atlases:
- DBS Intrinsic Template (DISTAL) atlas, v1.1 (Ewert et al., 2017): The atlas is openly available via the Lead-DBS knowledge base (https://www.lead-dbs.org/helpsupport/knowledge-base/atlasesresources/atlases-2/) and comes pre-installed with the Lead-DBS software package.
- Johns Hopkins University (JHU) atlas parcellation (Faria et al., 2012): The atlas is openly accessible as a pre-installation within the Surf Ice software (https://www.nitrc.org/projects/surfice/).
- California Institute of Technology reinforcement learning atlas, v1.1 (CIT168; Pauli et al., 2018): The atlas is openly available via the Lead-DBS knowledge base (https://www.lead-dbs.org/helpsupport/knowledge-base/atlasesresources/atlases-2/) and comes pre-installed with the Lead-DBS software package.

Human research participants

Policy information about studies involving human research participants and Sex and Gender in Research.

Reporting on sex and gender

Proportions of self-identified gender in the sample are reported in the manuscript (Tables S1-8 & S11). No statistical analyses

Reporting on sex and gender

with regard to gender- or sex-specific groups have been performed as no differences relevant to the research question in focus were to be expected.

Population characteristics

Discovery cohorts:
Sweet spot and sweet streamline models were created based on data from eight international cohorts of bilaterally implanted DBS patients (N = 197, 394 DBS electrodes) receiving stimulation to the subthalamic region for treatment of one of four different brain disorders: dystonia (DYT; n = 70, 38 females), Parkinson's disease (PD; n = 94, 29 females), obsessive-compulsive disorder (OCD; n = 19, 10 females), and Tourette's syndrome (TS; n = 14, 3 females). Please refer to tables S1-5 for detailed and comprehensive group-level as well as patient-wise information on relevant demographic and clinical patient characteristics.

Retrospective validation cohorts:
PD and OCD streamline models were retrospectively validated based on an additional cohort of patients each. The validation cohort of PD patients (n = 32, 10 females) had been implanted to the subthalamic nucleus (STN), while OCD patients (n = 35, 18 female) received DBS to the ventral capsule/ventral striatum (VC/VS) region. Tables S6-8 comprise detailed cohort-averaged and patient-specific demographic and clinical information.

Prospective patient cases:
Two DBS patients (one with PD and OCD each) were prospectively reprogrammed and one additional patient with OCD was prospectively implanted and programmed based on the respectively corresponding streamline models. The first prospective reprogramming case comprised a 67-year-old male patient with PD receiving DBS targeted to the STN, and the second case a 21-year-old female patient with OCD implanted to the VC/VS region. The surgical case was a 32-year-old male patient with OCD receiving STN-DBS. Table S11 lists more detailed patient-specific information on these three patient cases.

Recruitment

Retrospectice data:
Each dataset relied on different recruitment parameters depending on the respective study type (as listed in Tables S1 & S6). Further details on recruitment procedures are either described in our previous publications, i.e., Horn et al., 2017, Ann. Neurol & Horn et al., 2019, Brain (PD cohorts from Berlin and Würzburg), or in the original source publication(s) by each respective collecting site. These latter comprised studies by Dai et al., 2022, J. Neurol. (TS discovery cohort from Shanghai), Vissani et al. (2019), J. Neural Eng. (TS discovery cohort from Pisa/Milan), Tyagi et al., 2019, Biol. Psychiatry (OCD discovery and model validation cohort from London), Polosan et al. (2019), Transl. Psychiatry (OCD discovery cohort from Grenoble), Lin et al., 2019, J. Neurosurg. & He et al., 2021, Front. Neurol. (DYT discovery cohort from Shanghai), Ostrem et al., 2011 & 2016, Neurology (DYT disovery cohort from San Francisco), Butenko et al., 2022, NeuroImage Clin. (model validation cohort from Würzburg), McLaughlin et al., 2021, Contemp. Clin. Trials Commun. (model validation cohort from Boston), as well as Baldermann et al., 2019, Biol. Psychiatry & Li et al., 2020, Nat. Commun. (model validation cohort from Cologne).

Prospective patient cases:
-PD reprogramming case from Würzburg, Germany: The PD patient from Würzburg was investigated within the ongoing clinical service of the inpatient DBS program at University Hospital Würzburg. Reprogramming took place based on a multidisciplinary assessment of DBS location and streamline information.
-OCD reprogramming case from Boston, MA, USA: This patient was investigated within the ongoing clinical service of the DBS program at Massachusetts General Hospital (MGH; psychiatry and neurosurgery departments). Reprogramming for clinical purposes took place based on a multidisciplinary assessment of DBS location and streamline information.
-OCD-DBS implantation case from São Paulo, Brazil: This patient was recruited within the regular surgical service of Clínica de Dor e Funcional after classification as a refractory case of OCD, associated with depression. He underwent evaluation by a neurologist, psychologist and two functional neurosurgeons prior to the analysis of the Ethics Board Commite of the State of Rio Grande do Sul, who finally aproved the DBS surgery proposed by the team.

Ethics oversight

This research complied with all relevant ethical regulations and post-hoc analyses were approved by the institutional review board at Charité – Universitätsmedizin (Berlin, Germany; master vote EA2/186/18). Procedures of clinical trials and studies leading to the collection of the herein analyzed data were approved by the individual institutional review boards at each of the respective collecting sites (DBS centers in San Francisco, Shanghai, Pisa/Milan, London, Grenoble, Berlin, Würzburg, Boston, & Cologne). They were all carried out in accord with the declaration of Helsinki from 1975, and all participants signed an informed consent prior to study participation. Participants received no compensation in exchange for their participation in the trials and studies.

Note that full information on the approval of the study protocol must also be provided in the manuscript.

Field-specific reporting

Please select the one below that is the best fit for your research. If you are not sure, read the appropriate sections before making your selection.

☒ Life sciences ☐ Behavioural & social sciences ☐ Ecological, evolutionary & environmental sciences

For a reference copy of the document with all sections, see nature.com/documents/nr-reporting-summary-flat.pdf

Life sciences study design

All studies must disclose on these points even when the disclosure is negative.

Sample size

Since neuropsychiatric disorders represent comparably recent and rare applications of DBS to the STN target, available samples – especially in TS and OCD – are largely restricted by the small number of surgeries performed world-wide to date. In the TS cohort, we included the full

number of patients stimulated to the STN in this disease globally available at the time of analyses. Overall, we were able to identify two independent datasets in all four disorders for the model set-up (discovery cohorts) that linked DBS sites to improvements in respective primary clinical outcome.

Given the exploratory nature of our study, an a priori power analysis was not straight-forward. Our initial assumption of expected effect sizes was based on Li et al., 2020, Nat. Commun, and Treu et al. 2020, Neuroimage, with an r of approximately 0.4 for reported correlations between empirical clinical outcomes and estimated gain scores. Given the natural restrictions in available sample sizes described above, we calculated a "compromise" type power analysis using G*Power, v 3.1.9.6 (Faul et al., 2007; 2009, Behav. Res. Methods) to determine the power of our analysis based on the accessible data per disorder to detect the assumed effect. Given a β/α ratio of 1, the available DYT sample (n = 56 in the main cohort) used for model set-up was powered to 0.94 (implied α/β error probability = 0.06), the PD sample (n = 94) to 0.98 (implied α/β error probability = 0.02), the TS sample (n = 14) to 0.77 (implied α/β error probability = 0.23), and the OCD sample (n = 19) to 0.81 (implied α/β error probability = 0.19) for detecting the assumed effect size.

In additon, we performed a total of five validation experiments based on data unseen by the models, featuring two retrospective patient cohorts, one comprised of patients with PD (N = 32) and one of patients with OCD (N = 35, spanning three institutions) as well as three prospective patient cases. To our knowledge, this is the largest transdiagnostic study of its kind.

Data exclusions	All participants with complete neuroimaging and clinical outcome scores in the respective primary assessment were included in our analyses.
Replication	We assessed overall reproducibility of sweet spot and sweet streamline profiles in their ability of explaining clinical improvements in hold-out data using a five-fold cross-validation design, across two independent datasets per disease type from a total of seven institutions. Additionally, the main study result (i.e., the topographical organization of sweet streamlines) was re-assessed in four normative and two disease-matched connectomic resources of different properties to scrutinize the influence of choice of a specific tractogram. These additional analyses revealed a largely similar organizational pattern of dysfunction mappings irrespective of which connectome the attributions had been informed on. Moreover, streamline models in two exemplary disorders (OCD and PD) were retrospectively validated in independent (out-of-sample) data of one additional patient cohort each (with the OCD validation sample spanning data pooled across three institutions).
Randomization	Our study compared optimal connectivity and focal stimulation sites associated with symptom improvement in relation to precisely placed DBS electrodes between four different disease cohorts. Group affiliation of patients was thus determined by disorder category. This approach did not involve any form of experimental manipulation, comparison of conditions, or stimulus presentation, and the majority of results (with the exception of the three prospective patient cases for model validation) were based on retrospective data analysis. Instead of prospective randomization, we leveraged incidental variability in electrode placement within each disease-cohort, which can be presumed to be random.
Blinding	Secondary analysis of retrospective data:<br>Blinding was not relevant for the main body of our study which consisted of a secondary analysis of existing datasets. To minimize the risk of observer bias, we tested the explanatory value of both DBS sweet spot and sweet streamline profiles for clinical outcome variability within the respective clinical outcome measure in hold-out data in a five-fold cross-validation design (and across connectomic resources, in the case of streamline profiles). We additionally performed retrospective model validations based on two additional independent datasets of two of the investigated disorders (OCD and PD).<br><br>Prospective patient cases:<br>-PD reprogramming case from Würzburg, Germany: The patient was blinded to the DBS settings (clinically optimized vs. streamline-informed) and evaluated by an independent physician blind to the programming conditions.<br>-OCD reprogramming case from Boston, MA, USA: The patient was reprogrammed for clinical purposes, after clinically optimized stimulation parameters had failed to provide symptom relief. Thus, neither the patient nor the team of treating physicians was blinded to the activation of DBS nor to the programming condition (clinically optimized vs. streamline-informed) during the postsurgical evaluation of stimulation effects.<br>-OCD-DBS implantation case from São Paulo, Brazil: Given that only one condition was tested (streamline-optimized DBS), neither the patient nor the team of treating physicians was blinded to the activation of DBS during the postsurgical evaluation of stimulation effects.

Reporting for specific materials, systems and methods

We require information from authors about some types of materials, experimental systems and methods used in many studies. Here, indicate whether each material, system or method listed is relevant to your study. If you are not sure if a list item applies to your research, read the appropriate section before selecting a response.

Materials & experimental systems

n/a	Involved in the study	
☒	☐	Antibodies
☒	☐	Eukaryotic cell lines
☒	☐	Palaeontology and archaeology
☒	☐	Animals and other organisms
☒	☐	Clinical data
☒	☐	Dual use research of concern

Methods

n/a	Involved in the study	
☒	☐	ChIP-seq
☒	☐	Flow cytometry
☐	☒	MRI-based neuroimaging

Magnetic resonance imaging

Experimental design

Design type	Individualized structural MRI and/or CT of the head combined with normative structural human connectome data
Design specifications	Patient-specific structural MRI or CT scans of the head were used to localize stimulation sites (Horn & Kühn, 2014, Neuroimage; Horn et al., 2019, Neuroimage; Neudorfer et al., 2023, Neuroimage). Accounting for individual stimulation parameters, E-fields were further calculated to measure the stimulation impact on surrounding anatomy.<br><br>Based on these patient-specific electrode localizations and stimulation volumes, analysis (1) relied on voxel-wise weighting of the stimulation effect by its impact on clinical outcomes in each disorder-wise primary clinical outcome measure using DBS Sweet Spot Mapping (Horn et al., 2022, Proc. Natl. Acad. Sci; Neudorfer et al., 2023, Neuroimage).<br><br>In analysis (2), six different normative human connectomes (four healthy & two disease-matched) were employed to estimate clinically relevant structural connectivity across stimulation volumes using DBS Fiber Filtering (Baldermann et al., 2019, Biol. Psychiatry; Irmen et al., 2020, Ann. Neurol.; Neudorfer et al., 2023, Neuroimage).<br><br>The main connectomic resource employed to inform analysis part (2) consisted of a group connectome representative of average connectivity in a large healthy human sample (n = 985) (Li et al., 2020, Nat. Commun) from the HCP (Van Essen et al., 2013, Neuroimage). Analyses were further repeated using a normative ultra-high-resolution (760 µm isotropic) tractogram of a single healthy participant (Edlow et al., 2019, Sci. Data), the Basal Ganglia Pathway Atlas (Petersen et al., 2019, Neuron), as well as a customized pathway atlas (DBS Tractography Atlas, v2.1) explicitly created for this work using population-based fiber tracking and expert-defined pathways with focus on cortico-subthalamic inputs not represented in other atlas resources (for a detailed description of the creation of this atlas resource, please see the methods section in the main manuscript). Finally, two disease-matched connectomes were implemented to scrutinize the generalizability of dysfunction mappings in the face of disease-specific connectivity alterations on the examples of PD and OCD. The former comprised the PPMI-85 Connectome (Ewert et al., 2018, NeuroImage) and the latter the OCD-06 Connectome which had been calculated for the purpose of the present study based on diffusion imaging data by n = 6 patients with OCD.
Behavioral performance measures	Datasets used disorder-specific scales to measure primary clinical outcomes (as delineated in Tables S1 & S6). These comprised the Burke-Fahn-Marsden Dystonia Rating Scale (BFMDRS) in DYT, the Unified Parkinson's Disease Rating Scale – Part III (motor part; UPDRS-III) in PD, the Yale Global Tic Severity Scale (YGTSS) in TS, and the Yale-Brown Obsessive-Compulsive Scale (Y-BOCS) in OCD. Stimulation-related change within these measures relative to baseline (pre- vs. postoperative under DBS ON, or postoperative DBS OFF vs. ON conditions in the case of PD) was related to voxels in the subthalamic zone (DBS Sweet Spot Mapping) or structural connectivity (DBS Fiber Filtering).

Acquisition

Imaging type(s)	Normative dMRI data of n = 985 healthy controls from the HCP 1,200 subjects release (Van Essen et al., 2013, NeuroImage) for the construction of the HCP 985 Connectome, of n = 1,065 healthy young adults (Yeh et al., 2016, PLoS Comput. Biol.) for the construction of the DBS Tractography Atlas, v2.1 (see Methods for details on the creation of this atlas resource), of n = 85 Parkinson's disease patients from the PPMI (Marek et al., 2011, Prog. Neurobiol.), of n = 6 OCD patients (Tyagi et al., 2019, Biol. Psychiatry), as well as individualized (patient-specific) preoperative structural MRI (n = 261) and postoperative MRI (n = 73) or CT of the head (n = 188).
Field strength	MRI data collected using 3T scanner
Sequence & imaging parameters	Preoperative T1 & T2-weighted MRI, postoperative T1-weighted MRI (n = 73) or CT (n= 188) of the head; each dataset used different structural imaging parameters, as described in the respective source publications (see Tables S1 & S6)
Area of acquisition	Whole brain

Diffusion MRI ☒ Used ☐ Not used

Parameters: -HCP data (see Van Essen et al., 2013, NeuroImage, for details): The gradient table included approximately 90 diffusion weighting directions plus 6 b = 0 acquisitions interpersed throughout each run. Diffusion weigthing consisted of 3 shells of b = 1000, 200, or 3000 s/mm^2 interpersed with an approximately equal numbr of acquisitions on each shell within each run.

-PPMI data (see Marek et al., 2011, Prog. Neurobiol., for details): Acquisition was performed along 64 uniformly distributed directions with b = 1000 s/mm^2 and a single b = 0 image. A single shot echo-planar imaging (EPI) sequence (116 × 116 matrix, 2 mm isotropic resolution, TR/TE = 900/88 ms, two-fold acceleration) was implemented.

-OCD data (see Tyagi et al., 2019, Biol. Psychiatry, for details): Diffusion-weighting was applied with b = 1500 s/mm^2 along 128 directions uniformly distributed on the sphere and seven b = 0s volumes were acquired. For distortion correction a reversed phase encoding direction was applied for all acquisitions (resulting in 270 volumes in total).

Preprocessing

Preprocessing software	MATLAB-based Lead-DBS software, v3.0 (Neudorfer et al., 2023, Neuroimage) was used for preprocessing, including toolbox-internal dependencies on SPM12 and ANTs: https://github.com/netstim/leaddbs

Normalization	Multispectral spatial normalization using the SyN approach included in ANTs (http://stnava.github.io/ANTs/) with the 'Effective: low variance + subcortical refinement' preset in Lead-DBS, v3; normalization warp-fields were further visually inspected and manually optimized using the WarpDrive tool as included in Lead-DBS (Oxenford et al., 2023, MedIA), if needed, with particular attention to the STN as the anatomical structure in focus.
Normalization template	ICBM 2009b NLIN Asymmetric non-linear 2009b MNI152
Noise and artifact removal	Biasfield correction; correction for potential intraoperative brain shift via an automatized subcortical refinement module (as implemented in Lead-DBS, v3)
Volume censoring	Volume censoring is a motion denoising method used for functional MRI (fMRI) images. Since no fMRI data was analyzed in this study, volume censoring was not applied.

Statistical modeling & inference

Model type and settings	Mass-univariate analysis based on an in-sample spatial Spearman's correlation and subsequent validation of explanatory value of sweet spot and sweet streamline profiles for clinical outcome variability in a five-fold cross-validation design across two independent data sets per disease category; specificity analysis to test the predictive utility of disease-wise sweet streamline models for clinical outcome in all remaining three disorders; additional retrospective validation of the capability of streamline models to account for clinical outcome variance in independent (out-of-sample) patient cohorts
Effect(s) tested	Correlation between stimulation site connectivity (in the case of DBS Fiber Filtering) or activated voxels (in the case of DBS Sweet Spot Mapping) and change in pre- to post-treatment severity (or change in severity under postoperative ON vs. OFF DBS conditions in the case of PD) within the respective primary clinical outcome scale: BFMDRS in DYT, UPDRS-III (motor part) in PD, YGTSS in TS, and Y-BOCS in OCD

Specify type of analysis: ☒ Whole brain ☐ ROI-based ☐ Both

Statistic type for inference (See Eklund et al. 2016)	Voxel-wise
Correction	Validation of the generalizability of mass-univariate results in hold-out data using five-fold cross-validation and in six different connectomic resources (in the case of DBS Fiber Filtering results); model validation based on independent (out-of-sample) data of additional patient cohorts

Models & analysis

n/a	Involved in the study
☒	☐ Functional and/or effective connectivity
☒	☐ Graph analysis
☒	☐ Multivariate modeling or predictive analysis
